# Characteristics of molecular markers associated with chloroquine resistance in *Plasmodium vivax* strains from vivax malaria cases in Yunnan Province, China

**DOI:** 10.1186/s12936-023-04616-0

**Published:** 2023-06-11

**Authors:** Hongyun Ding, Ying Dong, Yan Deng, Yanchun Xu, Yan Liu, Jing Wu, Mengni Chen, Canglin Zhang, Weibin Zheng

**Affiliations:** 1 Yunnan Institute of Parasitic Diseases Control, Yunnan Provincial Key Laboratory, Yunnan Centre of Malaria Research, Pu’er, 665000 China; 2 Faculty of Life Science and Technology, Kunming University of Science and Technology, Kunming, 650500 China; 3 Center for Disease Control and Prevention, Baoshan, 678000 China

**Keywords:** Molecular marker, Multidrug resistance 1 gene, *Plasmodium vivax*, Vivax malaria, Strains, Yunnan Province

## Abstract

**Background:**

Chloroquine (CQ) has been the preferred clinical treatment for vivax malaria in Yunnan Province since 1958, with over 300,000 patients. This study aimed to help make trend predictions regarding variations the in anti-malarial drug susceptibility of *Plasmodium vivax* distributed in Yunnan Province and effectively implement monitoring measures on the efficacy of anti-malarial drugs for vivax malaria.

**Methods:**

Blood samples collected from patients with mono-*P. vivax* infections were employed in this study based on the principle of cluster sampling. The whole gene of *P. vivax* multidrug resistance 1 protein gene (pvmdr1) was amplified by nested-PCR techniques and the PCR amplification produce were sequenced by Sanger bidirectional sequencing. The mutant loci and haplotypes of coding DNA sequence (CDS) were identified by comparison with the reference sequence (NC_009915.1) of the *P. vivax* Sal I isolate. Parameters such as Ka/Ks ratio were calculated using MEGA 5.04 software.

**Results:**

A total of 753 blood samples from patients infected with mono-*P. vivax* were collected, of which 624 blood samples yielded the full gene sequence (4392 bp) of the pvmdr1 gene, with 283, 140, 119, and 82 sequences from 2014, 2020, 2021 and 2022, respectively. A total of 52 single nucleotide polymorphic (SNP) loci were detected for the 624 CDSs, of which 92.3% (48/52), 34.6% (18/52), 42.3% (22/52), and 36.5% (19/52) SNPs were detected in 2014, 2020, 2021 and 2022, respectively. All of 624 CDSs were defined for a total of 105 mutant haplotypes, with CDSs of 2014, 2020, 2021, and 2022 containing 88, 15, 21, and 13 haplotypes, respectively. Of the 105 haplotypes, the threefold mutant haplotype (Hap_87) was the starting point for stepwise evolution, and the most drastic tenfold mutations were Hap_14 and Hap_78, and the fivefold, sixfold, sevenfold, and eightfold mutations.

**Conclusions:**

In the majority of vivax malaria cases in Yunnan Province, most of them were infected with strains carrying demonstrating highly mutated in pvmdr1 genes. However, the dominant mutation strains types varied from year to year, which warrants further exploration in order to confirm the correlation between with phenotypic changes in *P. vivax* strains and their susceptibility to anti-malarial drugs such as chloroquine.

**Supplementary Information:**

The online version contains supplementary material available at 10.1186/s12936-023-04616-0.

## Background

Chloroquine (CQ) was first synthesized in 1934 [1], and has been widely used in Yunnan Province since 1960 [2]. In the late 1950s, CQ-resistant *Plasmodium falciparum* was found in Colombia and along the Thai-Cambodian border [3], and also in 1973 [4, 5] and 1974 [6], the emergence of CQ-resistant *P. falciparum* was reported in the Yunnan and Hainan provinces of China. Increasing prevalence of CQ-resistant *P. falciparum* has been continuously observed thereafter [5], and negative effects of drug resistance has also been noted.

Initially, CQ-resistant *P. falciparum* was tested in Yunnan Province using the in vivo test method recommended by the World Health Organization (WHO) [7]. Between 1981 and 1983, the widespread distribution of CQ-resistant *P. falciparum* in the major malaria-endemic areas of Yunnan Province was found by using the in vivo 4-week method [7]. In view of this, artemisinin, pyronaridine, and the compound of piperaquine with sulfadoxine have been used to replace CQ in the treatment of falciparum malaria patients in Yunnan since 1983 [8]. However, the "4-week method" with its low compliance and the test results easily affected by the patients immunity are encountering difficulties in practice when being applied to the large-scale and longitudinal monitoring of malaria CQ sensitivity [8]. Therefore, Liu et al*.* [9] successfully introduced the in vitro microscopic method for testing CQ resistance in *P. falciparum* in China [10], and used WHO standardized CQ applicator plates [10, 11], self-developed CQ applicator plates, and accompanying reagents [8] to investigate the susceptibility of *P. falciparum* to CQ, amodiaquine, piperaquine, and various other anti-malarial drugs in Yunnan and Hainan provinces of China from 1984 to 2002. The monitoring results showed that *P. falciparum* distributed in Yunnan and Hainan provinces were highly resistant to CQ, amodiaquine, and piperaquine, but the susceptibility of *P. falciparum* to CQ was restored after discontinuing or reducing the use of CQ [12].

CQ has been the preferred treatment for clinical episodes of vivax malaria in Yunnan Province since 1958 [2, 13–15], with over 300,000 patients (treated with a total dose of 1200 mg orally over 3 days) in the last four decades alone, according to incomplete statistics[16–21]. However, while the challenge of CQ resistance in *P. falciparum* has been a subject of great concern, evaluation of CQ's efficacy in treating vivax malaria patients has been rarely conducted. From April 2016, when the last indigenous vivax malaria case in China was reported in Yunnan Province [22], to the end of 2022, a cumulative total of 1371 imported cases infected with *Plasmodium vivax* parasites abroad were identified, including cases introduced abroad in Myanmar, Nigeria, the Democratic Republic of the Congo (DRC), Angola, and Cameroon, but predominantly in Southeast Asian countries, particularly Myanmar, which had the highest number of introduced cases [16, 22, 23].

Data show that CQ resistance in *P. vivax* was first identified in Papua New Guinea in 1989 [24], followed by reports of cases infected with CQ resistance *P. vivax* in Indonesia [25], northern Myanmar [26], India [27], and Vietnam [28]. Myanmar, which is located between South and Southeast Asia, is considered a high-risk transmission area for drug-resistant parasites [29]. Zeng et al*.* [30], using an in vitro microscopic method, observed that in the border area between Myanmar and China 4.4% (2/46) of all clinical isolates of *P. vivax* had CQ 50% inhibition concentration (IC50) values of above 220 nM, which exceed the susceptibility threshold by a factor of 1.5. A study conducted on amodiaquine (AQ) and CQ, each in combination with sulfadoxine-pyrimethamine (SP) in Papua New Guinea had a failure rate of more than 10% in the treatment of vivax malaria patients [31]. Ratcliff et al*.* [32] used CQ alone to treat vivax malaria patients in Indonesia and had a failure rate of 15% in the early stages and up to 65% by 28 days, but the possibility that this data was confounded by relapse events has not been excluded, which causes difficulty in assessing the efficacy of anti-malarials for *P. vivax* in vivo [33, 34]. New rounds of infections and recurrent intraerythrocytic infections caused by the activation of *P. vivax* hypnozoite parasites are confounding factors that must be guarded against in the in vivo assessment of anti-malarial drug efficacy in highly endemic areas [35]. Secondly, the lack of in vitro culture methods for *P. vivax* makes it difficult to directly transfer *P. vivax,* and these in vitro testing methods, such as isotopic methods [36] and microfluorimetric methods [37], have always been used for the drug susceptibility assays of *P. falciparum*. Animal model methods, which can compensate for the inability to obtain batches of *P. vivax* for in vitro testing are also impractical due to the difficulty in establishing animal models and the unsustainability in supply of primates [38]. Furthermore, with the continuous effectiveness of malaria control interventions, it has become increasingly challenging to find vivax malaria cases that meet the eligibility criteria for evaluating *P. vivax* drug resistance [39]. Therefore, the WHO proposed in 2018 that the lack of systematic evaluation of anti-malarial efficacy could be compensated by the optional use of molecular marker surveillance [39].

Previous studies have demonstrated that mutations in dihydrofolate reductase gene (*pvdhfr*) and dihydropteroate synthase gene (*pvdhps*) are associated with the development of resistance to SP in *P. vivax* [40, 41], while mutations in multidrug resistance 1 protein gene (*pvmdr1*) of *P. vivax* is one of the markers indicative of those with resistance to CQ [42]. A survey by Zeng et al*.* [30] found that the G698S substitution in the *pvmdr1* of *P. vivax* population distributed in the border area between Myanmar and China was associated with reduced susceptibility to CQ, artesunate and dihydroartemisinin. In Indonesia, Suwanarusk et al*.* [43] found that the geometric mean CQ half-inhibition concentration of 283 nM in Y976F mutant isolates was significantly higher than that of 44.5 nM in wild-type strains. In Thailand, monitoring the distribution and extent of both Y976F and F1076L mutations in the *pvmdr1* has facilitated stage-specific evaluation of changes in the susceptibility of *P. vivax* to anti-malarial drugs [44]. Although there have been instances of CQ treatment failure in vivax malaria cases in Guyana, molecular marker monitoring has not further shown specific polymorphisms in the *pvmdr1*. The country chose not to adjust its current anti-malarial drug policy, indicating that the susceptibility of *P. vivax* to antimalarials remains stable [45]. However, among numerous studies on the mutation polymorphism of *pvmdr1* gene, only a few ones are based on whole gene sequences [46, 47]. The main reason for not always targeting full gene sequences is that the 3′ end of *pvmdr1* gene is not easily sequenced due to the presence of many special structures.

In order to help fully revealing of the *pvmdr1* gene polymorphism degree in *P. vivax* strains infected in vivax malaria cases from Yunnan Province, China, and avoid forming a single-faceted experience of taking the research results around the China-Myanmar border as the currently actual situation in Yunnan Province, and begin carrying out the molecular surveillance plan on anti-malarial drug susceptibility of *P. vivax* in Yunnan Province [39], this study not only explored a realistic first-generation sequencing methods for *pvmdr1* whole gene, one of the molecular markers of CQ resistance, but also finished the longitudinal comparison on the *pvmdr1* characteristics in *P. vivax* strains collected from vivax malaria patients diagnosed in Yunnan Province in 2014 and from 2020 to 2022 following the strategy of segmented sequencing of the *pvmdr1* full gene, and the findings are reported below.

## Methods

### Study subjects and blood samples

A cohort study was conducted using all vivax malaria cases diagnosed in Yunnan Province, that were available in the China Disease Surveillance Information Reporting System (CDSIRS). However, this study only included the cases in 2014, 2020, 2021, and 2022 based on the principle of cluster sampling due to the limited workload of whole gene sequencing for *pvmdr1*. Each case was initially diagnosed by local county-level laboratories in Yunnan province, and then confirmed by the Yunnan Province Malaria Diagnosis Referent Laboratory (YPMDRL) to be mono-*P. vivax* infection through both light microscopy and genetic testing. The genetic test for *P. vivax* was conducted by the YPMDRL. The primer sequences, reaction conditions, and reaction system of the genetic test for *P. vivax* are shown in (Additional file 1). The information on sex, age, initial diagnosis, place of detection, and place of introduction of the vivax malaria cases was obtained from the registration files in CDSIRS. Peripheral venous blood was collected from all vivax malaria cases during acute episodes and dried blood dots (DBD) on filter paper were gathered for in-depth analysis of *Plasmodium* genetic sequencing.

### Confirmation of the malaria infection source

The indigenous infection malaria cases included those who had no history of travel to epidemic areas outside Yunnan Province within 30 days before the onset of malaria; The introduced malaria cases included those who had a history of migration from malaria endemic areas, such as Myanmar and Africa, within 30 days of malaria onset.

### Extraction of *Plasmodium *genomic DNA and PCR amplification of *pvmdr1* gene

Three 5 mm diameter DBDs were used to extract *Plasmodium* genomic DNA according to the instructions given in the QIAgen Mini Kit (DNA Mini Kit, QIAamp, Germany), which were stored at − 20 °C.

A strategy of segmented PCR amplification and sequencing was adopted in order to obtain the whole gene sequence of *pvmdr1*. The chromosomal reference sequence (ID: NC_009915.1) of *P. vivax* Sal I strain was used as a template to design the amplification of fragment 1 (F1, 365277-366271 nt), fragment 2 (F2, 364360-365400 nt), fragment 3 (F3, 363489-364447 nt), fragment 4 (F4, 362559-363620), and fragment 5 (F5, 361387-362957 nt) of the *pvmdr1* gene (Additional file 2). The primers names and thieves’ sequences, target fragment lengths, and reaction conditions for the nested PCR amplification of the five regions are detailed in Table 1. The amplification products of the second round of the five regions were expected to be 995 bp, 1041 bp, 959 bp, 1062 bp, and 1571 bp long, respectively.

**Table 1:** The primers names and thieves sequences, target fragment lengths for the nested PCR amplification the *pvmdr1* gene* in P. vivax* strains

Fragment	Nested PCR	Primer names	Primer sequences (5′ → 3′)	Product length (bp)	Amplification interval
F1	First round	DonMD-1-1F	TAACTCCTCACCGTTTGGGAAT	1245	365,143–366,387
		DonMD-1-1R	TCATTGTTTGGTTGCTGGTTGC		
	Second round	DonMD-1F	GGTGTGTATATCTTGAGTTTGCAT	995	365,277–366,271
		DonMD-1R	CGTGTACTTACTGTACAGCTTT		
F2	First round	DonMD-2-2F	TTTATTACCATATTTACGTACGCAAG	1385	364,189–365,573
		DonMD-2-2R	ATGATGATCGTAATTCTGTTTTCG		
	Second round	DonMD-2F	TAACAACACCATGTCCATCATCG	1041	364,360–365,400
		DonMD-2R	TTAGATGCATTAGAACCCACCAG		
F3	First round	DonMD-3-3F	CAACATCAAGTATAGTTTGTACAGC	1368	363,295–364,662
		DonMD-3-3R	TGAACATCTCTGTTAATATGTGCTG		
	Second round	DonMD-3F	TTAGTGTTTCGAAGAAGGTGCA	959	363,489–364,447
		DonMD-3R	GTAGAGGGAGTACTTATTCGAGT		
F4	First round	DonMD-4-4F	GCAGCATTTATAAGGACTCCG	1387	362,467–363,853
		DonMD-4-4R	CTCATCACGGTAGATTTGCC		
	Second round	DonMD-4F	GCCATTATAGCCCTGAGCATTAT	1062	362,559–363,620
		DonMD-4R	GACGTTTGGTCTGGACAAGATATC		
F5	First round/ Second round	DonMD-5-5F	GAGAAGGCTATTGATTATTCGAAT	1571	361,387–362,957
		DonMD-5-5R	TTAACTATGTTTACTACGGTTAAGGG		

The system used for all 10 PCR reactions was 1.5 μl of DNA template, 14.0 μl of PCR mix for 2 × Taq, and 0.5 μl each of upstream and downstream primers (10 μmol/l), and the volume was made to be 25.0 μl with the addition of ddH_2_O. The conditions for the 10 PCR reactions were the following: pre-denaturation at 95 °C for 4 min, followed by 32 cycles of amplification, with steps including denaturation at 95 °C for 45 s, annealing at 55–61 °C for 45 s, and extension at 72 °C for 90–150 s. The PCR amplification was terminated at 72 °C for 10 min. The end products of the second round of PCR amplification of the five regions were observed by 1.2% agarose gel electrophoresis, and the positive samples were sent to Guangzhou Tianyi Huiyuan Gene Technology Co for Sanger bidirectional sequencing.

### Gene polymorphism analysis

The sequencing results were collated using DNAStar 11.0 and BioEdit 7.2.5 software. Sequences from primer pairs DonMD-1F/DonMD-1R, DonMD-2F/DonMD-2R, DonMD-3F/DonMD-3R, DonMD-4F/DonMD-4R, and DonMD-5F/DonMD-5R amplification products were retrieved in order to obtain the coding DNA sequency (CDS) of 1 aa-250 aa, 251 aa-567 aa, 565 aa-847 aa, 848 aa-1154 aa, and 1153 aa-1464 aa of the *pvmdr1* gene. The overlap region of each CDS sequence was then removed and assembled along the 5′ → 3′ direction in order to obtain the CDS sequence of the *pvmdr1* whole gene (4392 bp), which was compared with the mRNA reference sequence of the *pvmdr1* gene (ID: XM_001613678.1), and when both Query Cover and Identifiers were > 98%, it indicated that the collated *pvmdr1* whole gene CDS sequence was correct.

The CDS sequence alignment file and the deduced amino acid sequence were created using MEGA 5.04 software, and then DnaSP 6.11.01 software was used to identify the haplotypes and single nucleotide polymorphism (SNP) loci of the CDS strand of the *pvmdr1* gene and their mutation types (synonymous/missense). Nucleotide diversity (π), expected heterozygosity (He) and Ka/Ks ratio were calculated, and sequence multiplicity mutations were identified. All base substitutions were confirmed by checking the sequence file ".seq" against the corresponding ".ab1" file; Ka/Ks ratios > 1, = 1, and < 1 indicated positive, neutral and negative selection in the population, respectively. Network 10.0 software was used to create an evolutionary mediated network map of the various haplotypes, where the same locus in the CDS strand of the *pvmdr1* gene was repeated across haplotypes, showing a locus mutation that was defined as a 'reverse mutation'.

### Statistical analysis

The database for polymorphic analysis of the *pvmdr1* gene was created using Excel software, and the differences in SNP and haplotype detection rates between years were evaluated by Chi-square test (χ^2^) at the level of 0.05 in the "Data and descriptive statistics" module of IBM SPSS Statistics 21 software.

## Results

### Sample amplification information and PCR product amplification sequencing

A total of 753 cases infected with mono-*P. vivax* were diagnosed and confirmed in Yunnan Province in 2014, 2020, 2021, and 2022. The patients were predominantly young adult males (84.0%, 632/753), with a smaller number of females (16.0%, 121/753); 81.8% (616/753) of the patients were between 21 and 60 years of age (Table 2). The source of infection was predominantly Myanmar, accounting for 94.0% (708/753) of all cases, and the only indigenous cases of infection in Yunnan Province were reported in 2014 (4.0%, 15/338) (Table 2). The places of initial diagnosis were the following: 450 cases in Dehong (59.8%), 175 cases in Baoshan (23.2%), 41 cases in Lincang (5.4%), 22 cases in Kunming (2.9%), 18 cases in Dali (2.4%), 14 cases in Pu'er (1.9%), 7 cases in Nujiang (0.9%), 7 cases in Xishuangbanna (0.9%), 5 cases in Honghe (0.7%), 3 cases in Lijiang (0.4%), 3 cases in Wenshan (0.4%), 2 cases in Chuxiong (0.3%), 2 cases in Qujing (0.3%), 2 cases in Yuxi (0.3%), 1 case in Zhaotong (0.1%), and 1 case in Diqing (0.1%). The number of cases diagnosed in different years and prefectures can be seen in Additional file 3.

**Table 2:** Information on vivax malaria cases diagnosed in Yunnan Province from January to December 2014 and from January 2020 to December 2022

	Total (n, F%)	2014 (n, F%)	2020 (n, F%)	2021 (n, F%)	2022 (n, F%)
Total	753 (100.0)	379 (50.3)	154 (20.1)	126 (16.7)	94 (12.5)
Gender					
Male	632 (84.0)	341 (90.0)	122 (79.2)	100 (79.4)	69 (73.4)
Female	121 (16.1)	38 (10.0)	32 (20.8)	26 (20.6)	25 (26.6)
Age					
0–20	117 (15.5)	47 (12.4)	25 (16.2)	23 (18.3)	22 (23.4)
21–60	616 (81.8)	329 (86.8)	125 (81.2)	97 (77.0)	65 (69.1)
60–	20 (2.7)	3 (0.8)	4 (2.6)	6 (4.8)	7 (7.4)
Source of infection					
Myanmar	708 (94.0)	338 (89.2)	153 (99.4)	125 (99.2)	92 (97.9)
Africa	13 (1.7)	11 (3.0)	–	1 (0.8)	1 (1.1)
Laos	12 (1.5)	12 (3.2)	–	–	–
Thailand	3 (0.4)	2 (0.5)	–	–	1 (1.1)
Pakistan	2 (0.3)	1 (0.3)	1 (0.6)	–	–
Yunnan	15 (2.0)	15 (4.0)	–	–	–

Out of the 753 vivax malaria cases mentioned above, the *P. vivax* strains from 624 cases were amplified by segmented PCR and the products were sequenced to obtain the *pvmdr1* full gene sequence, with a success rate of 82.9% (624/753). The second round of product electrophoresis for the segmented nested PCR amplification of the *pvmdr1* gene from fragment F1 to fragment F5 is shown in Additional file 4, with target electrophoretic bands at approximately 1000 bp, 1000 bp, 1000 bp, 1100 bp and 1600 bp (Additional file 4).

### Nucleotide diversity of gene sequences

The complete CDSs of 624 *pvmdr1* genes with similarities (Identifiers) and coverage (Query Cover) > 98% regarding the reference sequences had been submitted for GenBank (GenBank ID including: BankIt2643392: OP559204-OP559462 and BankIt2680229: OQ614893-OQ615257. Two times submissions are to be held confidential until June 30 2023 and Oct 1, 2023, respectively). Nucleotide diversity (π) and Ka/Ks ratio were equal to 0.00087 and 3.6012, respectively, while π and Ka/Ks ratio were equal to 0.00096, 0.00078, 0.00075, 0.00076, and 3.6019, 3.6008, 3.6003, and 3.6004, in 2014, 2020, 2021 and 2022, respectively.

There were base pair substitutions at 52 loci comparison of 624 CDSs and referent sequences (ID: XM_001613678.1) (Tables 3, 4; Additional file 5). Of these, non-synonymous and synonymous mutations accounted for 46.2% (24/52) and 53.8% (28/52), respectively. Substitutions in the first, second, and third positions of the triplet codon accounted for 25.0% (13/52), 25.0% (13/52), and 50.0% (26/52), respectively. MAF (Minor allele frequency) locus is c.1587A > G (91.2%, 569/624) (Table 3). There were 19 single variable sites (36.5%, 19/52). In 2014, 2020, 2021, and 2022, the figures were 78.9% (15/19), 15.8% (3/19), and 5.3% (1/19), respectively, and no singleton mutation sites were detected in the 2020 sequence. The detection rates of single occurrence mutations were 73.7% (14/19), 10.5% (2/19), 5.3% (1/19), 5.3% (1/19), and 5.3% (1/19) for the Myanmar, African, Lao, Pakistani, and indigenous Yunnan strains, respectively (Table 4b); 21 new SNPs were detected, all of which occurred in the 2014 sequences. The detection rates of new SNPs were 95.2% (40/42), 2.4% (1/42), and 2.4% (1/42) for the Myanmar strain, the Lao strain, and the indigenous Yunnan strain, respectively (Tables 3, 4).

**Table 3:** SNPs of the *pvmdr1* gene in *P. vivax* strains

OD	TMD	Loci	AL	Coding	AAV	No. of all CDSs(n = 624)	Frequency				
							2014 (n = 283)		2020 (n = 140)	2021 (n = 119)	2022 (n = 82)
							IP (n = 271)	ID (n = 12)	IP (n = 140)	IP (n = 119)	IP (n = 82)
1	Inside	c.23^c^	C>T	CCC/C*T*C	P8L	64 (10.3%)	5 (1.8%)	0	19 (13.6%)	25 (21.0%)	15 (18.3%)
2	Inside	c.96^a^	G>C	GGG/GG*C*	G32G	2 (0.3%)	2 (0.7%)	0	0	0	0
3	Inside	c.132^b^	G>A	AAG/AA*A*	K44K	241 (38.6%)	98 (36.2%)	4 (33.3%)	53 (37.6%)	52 (43.7%)	34 (41.5%)
4	3	c.516	C>T	GGC/GG*T*	G172G	3 (0.5%)	2 (0.7%)	1 (8.3%)	0	0	0
5	Outside	c.930^c^	G>A	TTG/TT*A*	L310L	63 (10.1%)	2 (0.7%)	0	19 (13.6%)	25 (21.0%)	17 (20.7%)
6	Outside	c.936	C>T	AAC/AA*T*	N312N	5 (0.8%)	5 (1.8%)	0	0	0	0
7	Inside	c.1226	C>T	ACG/A*T*G	T409M	31 (5.0%)	11 (4.6%)	1 (8.3%)	8 (5.7%)	5 (4.2%)	6 (7.3%)
8	Inside	c.1477^c,b^	T>C	TTA/*C*TA	L493L	26 (4.7%)	25 (9.2%)	1 (8.3%)	0	0	0
9	Inside	c.1539^cb^	T>A	AGT/AG*A*	S513R	118 (18.9%)	69 (25.5%)	2 (16.7%)	27 (19.3%)	12 (10.1%)	8 (9.8%)
10	Inside	c.1559^b^	G>A	GGT/G*A*T	G520D	32 (5.1%)	21 (7.7%)	1 (8.3%)	7 (5.0%)	2 (1.7%)	1 (1.2%)
11	Inside	c.1587^b^	A>G	ACA/AC*G*	T529T	569 (91.2%)	227 (83.4%)	7 (58.3%)	136 (97.1%)	117 (98.3%)	82 (100%)
12	Inside	c.2092^b^	G>A	GGC/*A*GC	G698S	534 (85.6%)	256 (94.5%)	11 (91.7%)	120 (85.7%)	88 (73.9%)	59 (72.0%)
13	7	c.2499	G>T	ATG/AT*T*	M833I	9 (1.4%)	2 (0.7%)	0	3 (2.1%)	3 (2.5%)	1 (1.2%)
14	7	c.2533^c^	C>T	CTC/*T*TC	L845F	106 (17.0%)	54 (19.2%)	1 (8.3%)	12 (8.6%)	16 (13.4%)	23 (28.0%)
15	Outside	c.2582^c^	C>A	GCG/G*A*G	A861E	84 (13.5%)	37 (13.7%)	3 (25%)	23 (16.4%)	19 (16.0%)	2 (2.4%)
16	Inside	c.2722	A>C	ATG/*C*TG	M908L	624 (100%)	271 (100%)	12 (100%)	140 (100%)	119 (100%)	82 (100%)
17	9	c.2873	C>T	ACG/A*T*G	T958M	624 (100%)	271 (100%)	12 (100%)	140 (100%)	119 (100%)	82 (100%)
18	10	c.2927	A>T	TAC/T*T*C	Y976F	7 (1.1%)	7 (2.6%)	0	0	0	0
19	Inside	c.2990	A>G	AAG/A*G*G	K997R	4 (0.6%)	3 (1.1%)	1 (8.3%)	0	0	0
20	11	c.3201^a^	C>T	CTC/CT*T*	L1067L	2 (0.3%)	2 (0.7%)	0	0	0	0
21	11	c.3210^a^	C>T	TTC/TT*T*	F1070F	3 (0.5%)	3 (1.1%)	0	0	0	0
22	11	c.3226^c,b^	T>C	TTT/*C*TT	F1076L	472 (75.6%)	168 (62.0%)	8 (66.7%)	113 (80.7%)	110 (92.4%)	73 (89.0%)
23	12	c.3358	C>T	CTA/*T*TA	L1120L	8 (1.3%)	7 (2.6%)	0	0	1 (0.8%)	0
24	Inside	c.3634^a^	C>T	CTG/*T*TG	L1212L	7 (1.1%)	7 (2.6%)	0	0	0	0
25	Inside	c.3699^a^	G>A	AGA/A*A*A	E1233E	4 (0.6%)	4 (1.5%)	0	0	0	0
26	Inside	c.3774^a^	C>A	TCC/TC*A*	S1258S	2 (0.3%)	2 (0.7%)	0	0	0	0
27	Inside	c.3776^a^	C>T	TCC/T*T*C	S1259F	2 (0.3%)	2 (0.7%)	0	0	0	0
28	Inside	c.4044	C>T	ACC/AC*T*	I1348I	24 (3.8%)	12 (4.4%)	2 (16.7%)	7 (5.0%)	2 (1.7%)	1 (1.2%)
29	Inside	c.4074^b^	C>T	TCC/TC*T*	S1358S	68 (10.9%)	27 (1.0%)	3 (25%)	21 (15.0%)	7 (5.9%)	10 (12.2%)
30	Inside	c.4179^c,b^	G>C	AAG/AA*C*	K1393N	112 (17.9%)	68 (25.15)	3 (25%)	14 (10.0%)	14 (11.8%)	13 (15.6%)
31	Inside	c.4188	G>A	GAG/GA*A*	E1396E	7 (1.1%)	7 (2.6%)	0	0	0	0
32	Inside	c.4292^a^	A>G	AAC/A*G*C	N1431S	7 (1.1%)	7 (2.6%)	0	0	0	0
33	Inside	c.4349	C>T	TCG/T*T*G	S1450L	96 (15.4%)	48 (17.7%)	2 (16.7%)	23 (16.4%)	16 (13.4%)	7 (8.5%)
34	–	Loci with only 1 mutation (continued in Table 4)				19	15	0	0	3	1

OD, TMD, AL, AAV, IP, ID are Order, Transmembrane domain (Inside and Outside mean the inside and outside the digestive vesicle; the different Arabic numerals mean some transmembrane region), Loci, Alleles, Coding Amino acid variation, Imported, Indigenous, respectively

^a^Newly detected SNP

^b^Indicates multiple mutations at the locus

^c^Indicates that the locus detection is statistically significant between years

**Table 4:** SNPs of *pvmdr1* gene in *P. vivax* strains

Years	Loci with only 1 mutation		AL	Coding	AAV	TMD
	Number	Loci				
2014	1	c.543^a^	C>T	TAC/TA*T*	Y181Y	3
	1	c.553^a^	T>C	TTT/*C*TT	F185L	3
	1	c.744^a^	C>T	ACC/AC*T*	T248T	Inside
	1	c.1197^a^	A>G	GAA/GA*G*	E399E	Inside
	1	c.1309^a^	C>T	CCC/*T*CC	P437S	Inside
	1	c.1329^a^	C>T	ATC/AT*T*	I443I	Inside
	1	c.1659^a^	G>T	TCG/TC*T*	S553S	Inside
	1	c.1953^a^	G>T	GTG/GT*T*	V651V	Inside
	1	c.2016	G>C	AAG/AA*C*	K672N	Inside
	1	c.2288	C>T	GCG/G*T*G	A763V	Inside
	1	c.2356^a,c^	A>G	AGT/*G*GT	S786G	Inside
	1	c.3147^a^	T>C	GCT/GC*C*	A1049A	Inside
	1	c.3803^a^	G>T	AGT/A*T*T	S1268I	Inside
	1	c.3855^a^	C>T	TGC/TG*T*	C1285C	Inside
	1	c.4266^a^	G>A	AGG/AG*A*	R1422R	Inside
2021	1	c.3064	C>T	CTA/*T*TA	L1022L	Inside
	1	c.3832	C>T	CTG/*T*TG	L1278L	Inside
	1	c.4065	A>G	AAA/AA*G*	K1355K	Inside
2022	1	c.2524	T>A	TTT/*A*TT	F842I	7
Total	19					

The special marks and abbreviations in the Table 4 are the same as Table 3

The detection sizes of the 52 SNPs in the 2014, 2020, 2021, and 2022 sequences were 48 (92.3%, 48/52), 18 (34.6%, 18/52), 22 (43.3%, 22/52), and 19 (36.5%, 19/52), respectively (Tables 3, 4).

**Fig. 1:**
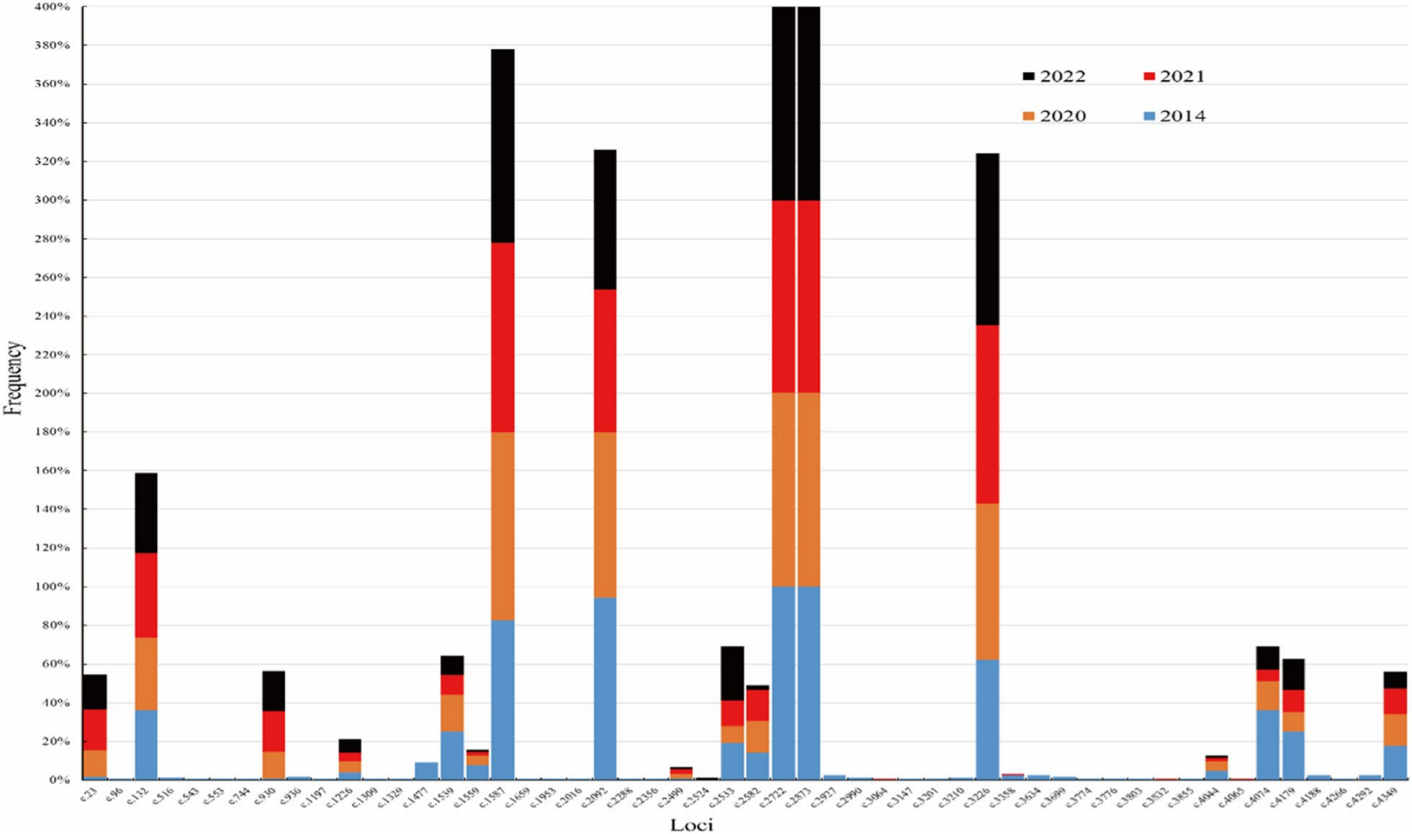
The change of detection frequency of 52 SNPs during in different years

The trends in SNPs detected in different years mainly include the following:

1. Of the 48 SNPs in 2014, 29 of them, including c.96G > C, c.516C>T, c.543C>T, and c.553T>C were individually detected, but only the disappearance of the c.1477T>C loci after 2014 was statistically significant (*χ*^*2*^ = 32.691, *P* < 0.001) (Tables 3, 4; Fig. 1; Additional file 6).
2. The 18 SNPs detected in 2020 were all also detected in the other 3 years, but only 2 SNPs, c.23C>T and c.930G>A, were detected at a lesser rate of 1.8% (5/283) and 0.7% (2/283) in 2014, compared with 13.6% (19/140), 21.0% (25/119), 18.3% (15/82) and 13.6% (19/140), 21.0% (25/119), and 20.7% (17/82) in the other 3 years. These differences were statistically significant (*χ*^*2*^ = 44.531, *P* < 0.001; *χ*^*2*^ = 55.180, *P* < 0.001). In addition, the lower detection rate of c.3226T>C in 2014 of 62.2% (176/283) was comparable to the lower detection rates of 92.4% (110/119) and 89.0% (73/82) in 2021 and 2022, respectively, and this was statistically significant (*χ*^*2*^ = 58.105, *P* < 0.001). In contrast, when comparing the two SNPs with higher detection rates in 2014, c.1539T>A (25.1%, 71/283) and c.4179G>C (25.1%, 71/283) with the detection rates of 10.1% (12/119) in 2021 (*χ*^*2*^ = 17.645, *P* < 0.001) and the detection rates of 10.8% (28/259) during 2020 and 2021 (*χ*^*2*^ = 18.599, *P* < 0.001), respectively, the differences were statistically significant (Table 3). In the fourth case, for the sudden increase and decrease in detection of c.2533 C>T and c.2582C>A in recent years, c.2533 C>T had a lesser detection rate (8.6%, 12/140) in 2020 compared to 28.0% (23/82) in 2022 (*χ*^*2*^ = 14.772, *P* < 0.001), which was statistically significant; c.2582C>A had a higher detection rate of 14.1% to 16.4% from 2014 to 2021 compared to 2.4% (2/82) detection rate in 2022 (*χ*^*2*^ = 10.361, *P* < 0.05), which was statistically significant (Tables 3, 4; Fig. 1; Additional file 6).

### Multiple mutations in *pvmdr1* gene CDSs

The complete CDS of the 624 *pvmdr1* genes were compared with the reference sequence (ID: XM_001613678.1) (haplotype Hap_1), and 105 haplotypes were identified (Hap_2 to Hap_106) (Table 5; Additional file 7), all are mutants of the reference sequence, with a total He equal to 0.9515, while the He was equal to 0.974, 0.886, 0.891, and 0.879 for 2014, 2020, 2021, and 2022, respectively. Of the 105 sample sequence haplotypes, there were always three to seven non-synonymous mutations in multiple mutation loci, with the nonsynonymous mutation c.2927A>T (Y976F) occurring in only four haplotypes (Hap_6, Hap_7, Hap_34 and Hap_60).

**Table 5:** Composition of haplotypes of *pvmdr1* gene CDSs from* P. vivax* strains in 2014 and 2020–2022

OD	Folds	Haplotype example	Fold loci composition of example	Types of the same folds	Total (n = 624)	Different between years			
						2014 (n = 283)	2020 (n = 140)	2021 (n = 119)	2022 (n = 82)
1	3	Hap_87, et al.	G698S/M908L/T958M, et al.	1	1 (0.2%)	1 (0.4%)	0	0	0
2	4	Hap_8, et al.	G698S/M908L/T958M/S1358S, et al.	5	27 (2.7%)	26 (9.2%)	1 (0.7%)	0	0
3	5	Hap_40, et al.	T529T/ G698S /M908L/T958M /F1076L, et al.	13	122 (19.6%)	45 (15.9%)	33 (23.6%)	24 (20.2%)	19 (23.2%)
4	6	Hap_91, et al.	P8L/L310L/T529T/M908L/T958M/F1076L, et al.	32	187 (30.0%)	79 (28.0%)	47 (33.6%)	38 (31.9%)	24 (29.3%)
5	7	Hap_24, et al.	K44K/T529T/G698S/M908L/T958M/F1076L/S1450L, et al.	33	227 (36.4%)	85 (30.0%)	52 (37.1%)	54 (45.4%)	36 (43.9%)
6	8	Hap_5, et al.	L493L/T529T/G698S/L845F/M908L/T958M/F1076L/E1233E, et al.	12	18 (2.9%)	16 (5.7%)	0	0	2 (2.4%)
7	9	Hap_3, et al.	K44K/T529T/G698S/L845F/M908L/T958M/F1076L/L1120L/S1450L, et al.	7	18 (2.9%)	17 (6.0%)	0	1 (0.8%)	0
8	10	Hap_14, et al.	K44K/G520D/T529T/G698S/A861E/M908L/T958M/F1076L/I1348I/S1450L, et al.	2	24 (3.8%)	14 (4.9%)	7 (5.0%)	2 (1.7%)	1 (1.2%)

ID is order

The detection sizes of the 105 haplotypes in the 2014, 2020, 2021, and 2022 sequences were 88 (83.8%, 88/105), 15 (14.3%, 15/105), 21 (20.0%, 21/105), and 13 (12.4%, 13/105), respectively (Additional files 7, 8). In addition, of the 105 haplotypes, the most moderate multiple mutation was threefold (Hap_87) and the most drastic multiple mutation was tenfold (Hap_14 and Hap_78) (Table 5; Additional file 7), and fivefold, sixfold, sevenfold and eightfold loci mutation haplotypes were predominant, and all these together accounted for 88.8% (554/624); threefold, fourfold, ninefold, and tenfold loci mutation haplotypes accounted for 0.2% (1/624), 2.7% (27/624), 2.9% (18/624), and 3.8% (24/624), respectively.

Furthermore, there was variance in detected haplotypes between different years. For example, the threefold loci mutation haplotype was detected in only one sample in 2014.

**Fig. 2:**
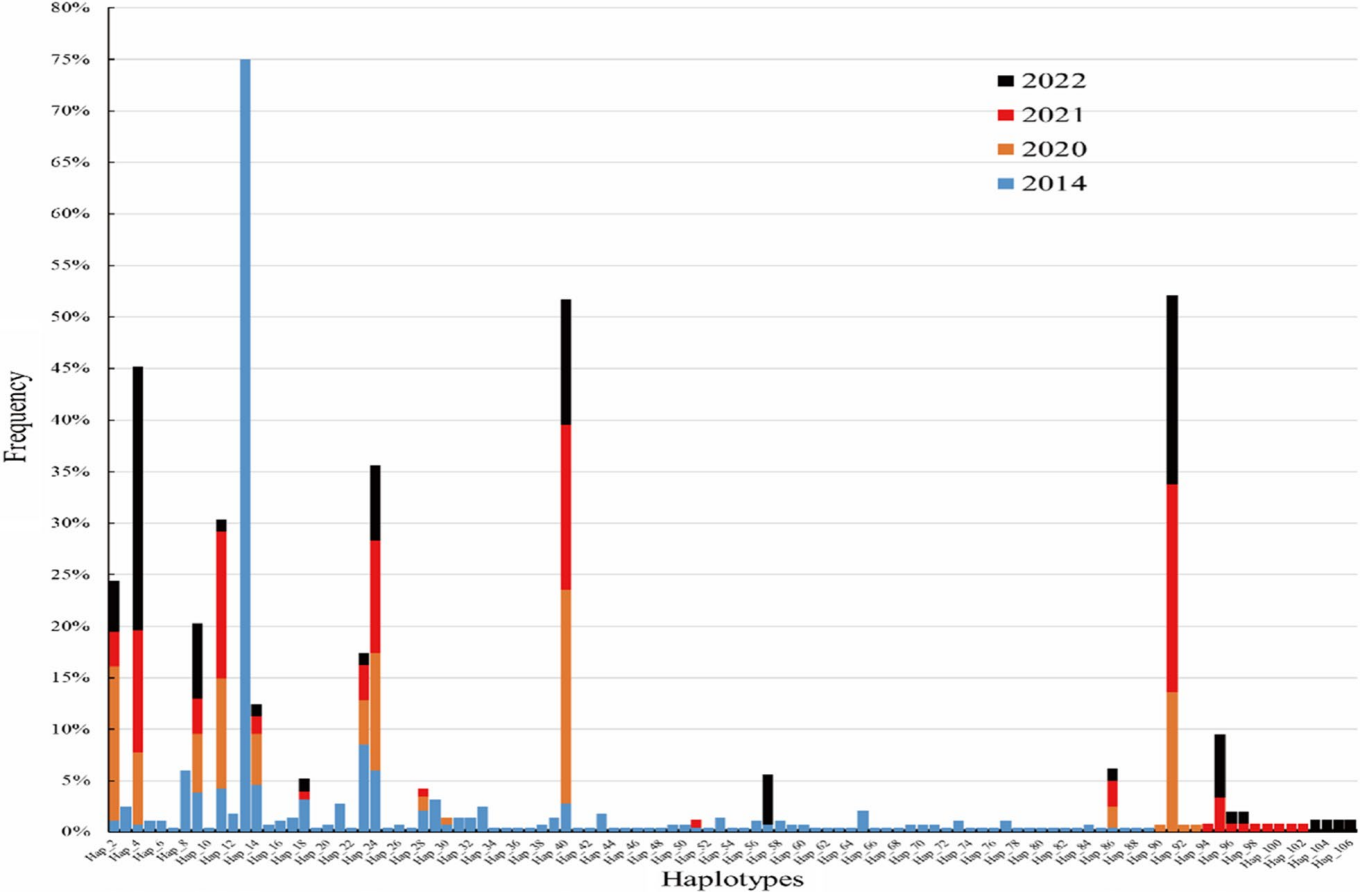
The change of detection frequency of 105 haplotypes during in different years

The trends in multiplex detections include the following:

1. a statistically significant difference between the 9.2% (26/283) detection rate of the fourfold mutant haplotype in 2014 and its decrease to 0.7% (1/140) in 2020, with no further detections in 2021 or 2022 (*χ*^*2*^ = 29.654, *P* < 0.001) (Table 5; Fig. 2; Additional file 8).
2. The increase in the detection of sevenfold loci mutant haplotypes between 2014 (30.0%, 85/283) and 2021 (45.4%, 54/119) was statistically significant (*χ*^*2*^ = 8.718, *P* < 0.05) (Table 5; Fig. 2; Additional file 8).
3. The detection rate of 5.7% (16/283) of the eightfold loci mutant haplotype in 2014 was statistically significant (*χ*^*2*^ = 15.089, *P* < 0.05) compared to the results from 2020 and 2021, where it was not detected (Table 5; Fig. 2; Additional file 8).
4. The detection rate of 6.0% (17/283) for the ninefold loci mutant haplotype in 2014 was statistically significant (*χ*^*2*^ = 8.762,* P* < 0.05) compared to results from 2020, wherein it was not detected (Table 5; Fig. 2; Additional file 8).

### Evolutionary analysis of multiple mutations in* pvmdr1* gene CDSs

**Fig. 3:**
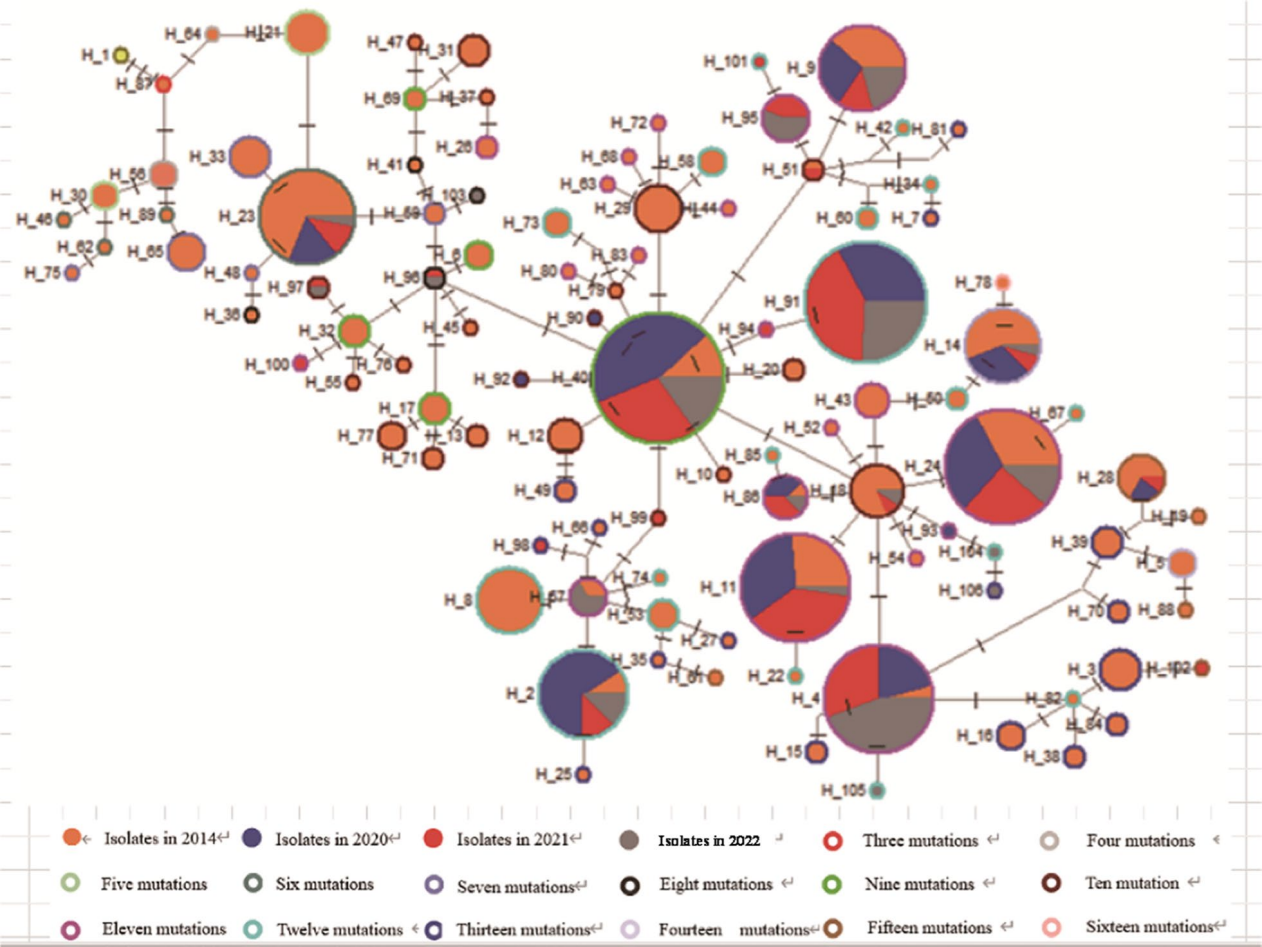
The medium network diagram for 105 haplotypes of *pvmdr1* CDS chain

The haplotype medium network diagram shows that the 105 sample sequence haplotypes starting with the reference sequence (ID: XM_001613678.1) (haplotype Hap_1), evolved via the most moderate threefold loci mutation (Hap_87, "G698S/M908L/T958M"), and further evolved stepwise along two branches of the fourfold mutation (Hap_56) and (Hap_64) (Fig. 3).

The evolution continued along the direction of Hap_56, after accumulating 11–12 loci mutations, to the most distal haplotypes Hap_78, Hap_19, Hap_28, Hap_61, Hap_88, and Hap_102. During the evolutionary path from Hap_64 to Hap_96 which involved undergoing four sequential loci mutations, 80.0% (12/15) of the haplotypes consisted only of sequences from 2014. Seven haplotypes, including Hap_64, were not affected by reverse mutations at nine loci (c.132, c.1477, c.1539, c.1559, c.1587, c.2092, c.3226, c.4074, c.4179), and the network evolution showed mutational multiplicity consistent with the mutational multiplicity identified by the sequences (Fig. 3; Additional file 7). The sequences from 2020 to 2022 show a relatively high concentration of these haplotypes that continue to evolve 1 to 5 steps from Hap_96, including Hap_40, Hap_91, Hap_2, Hap_11, Hap_86, Hap_14, Hap_24, and Hap_4, to the near end of the evolutionary direction of Hap_64, again showing a predominance of sequences from 2014, with 12 of the 14 haplotypes after Hap_4 all consisting entirely of 2014 sequences (Fig. 3).

In addition, out of the 105 haplotypes examined, only 15 demonstrated consistent results of multi-locus joint mutations between the network evolution analysis and sequence alignment (Additional file 7), while the remaining 90 haplotypes (87.5%, 90/105) showed a higher degree of multiplicity in the network evolutionary analysis compared to the sequence comparison owing to reverse mutations at c.132, c.1477, c.1539, c.1559, c.1587, c.2092, c.3226, c.4074, and c.4179, usually with 2–4 times reverse mutations (Additional file 9). Of the 90 haplotypes, the proportions of haplotypes with more than twofold, fourfold, sixfold, eightfold, and tenfold loci mutations in network evolution analysis were 6.7% (6/90), 50% (45/ 90), 36.7% (33/90), 5.6% (5/90), and 1.1% (1/90), respectively (Additional file 9).

## Discussion

The *pvmdr1* gene is located on chromosome 10, far from the telomere, in approximately 1/4 (366095/1419739) of *P. vivax*, which starts at the base of the gene (5'-ATG), consists of a complete open reading frame that can encode the 1464 amino acids of *Pv*MDR1, which belong to the membrane structural protein with 12 transmembrane domains located at the *P. vivax* digestive vesicles [48, 49]. The sequences length of the *pvmdr1* gene obtained in this study all showed highly conservative with the fixed a 1464 aa amino acid chain, which may be related to the fact that the *pvmdr1* gene is located far from the telomeres of chromosome 10 and is less likely to undergo fragment deletion or insertion due to genetic recombination [50, 51]. The success rate of 82.9% (624/753) obtained the *pvmdr1* full gene sequence also indicates that the experimental method for fragment PCR amplification and sequencing of *pvmdr1* gene has good stability in this study.

A total of 52 SNPs were detected in 624 *pvmdr1* gene sequences in this study, except for the 31 SNPs reported before, including c.23C>T (P8L), c.132G>A (K44K), and c.516C>T (G172G), etc., [43, 46, 47, 52], there were 21 other newly detected SNPs (Tables 3, 4), which may be due to the large sample size of this study and the analysis of the *pvmdr1* whole gene sequence. The concentration of newly detected SNPs and low-frequency SNPs in 2014 may be due to the fact that the study sample in that year included a richer population of *P. vivax* than in subsequent years and even included all the indigenous Yunnan strains (Tables 3, 4). But, based on that fewer new SNPs and fewer low-frequency SNPs were detected in the indigenous Yunnan strains than in other populations, it may suggest that the *P. vivax* population should be less intensely screened than the others populations.

Of the 18 SNPs that were detected in all years, eight loci mutations were frequently detected in Burmese strains, including c.1539T>A (S513R), c.2092G>A (G698S), c.2533C>T (L845F), c.2582C>A (A861E), c.2822A>C (M908L), c.2873C>T (T958M), c.3226T>C (F1076L), and c.4179G>C (K1393N) [46]. Of which, c.2822A>C ( M908L) and c.2873C>T (T958M) reached 100% detection, which was consistent with previous results of nearly 100% of these isolates found in the Myanmar Laza city [53], the China-Myanmar border [46, 51, 54], the Thai-Myanmar border [55, 56], and the Thai-Cambodia-Thailand-Lao border [47], which is also consistent with previous studies that have shown that the *pvmdr1* gene sequences all came from *P. vivax* populations in Southeast Asia, and *P. vivax* strains of the present study were mainly composed of Burmese strains, accounting for 94.0% (708/753). Meanwhile, although the Ka/Ks ratios in this study were much greater than 1, in view of no positive selection pattern of low-frequency mutation surges in the mediator network map, it may still be attributed to the combination of neutral selection and drug pressure screening that the c.2822A>C (M908L) and c.2873C>T (T958M) mutations were detected in all samples strains [46, 57]. Another mutation, c.3226T>C (F1076L), also produced by a combination of neutral selection and drug pressure [58], showed an increase in detection from 2014 to 2022 (Table 3; Fig. 1), approaching the previous detection rates of 75.7% (143/189) to 85% (97/113) of the Myanmar population [55, 59], but not reaching the level of the Ethiopian populations with 100% (55/55 and 28/28) detection [42, 60], which suggests that the Myanmar population is stably screened from both the neutral selection and drug pressure, and that the screening for the c.3226T>C (F1076L) mutation is still incomplete.

In contrast to the gradual increase in the detection of the c.3226T>C (F1076L) mutation, the 29 SNPs represented by the c.2927A > T (Y976F) mutation were only detected in the early 2014 sequence (Tables 3, 4), and the c.2927A>T (Y976F) mutation was not detected again after 2020, which is consistent with the conclusions drawn by several authors on *P. vivax*. The c.2927A>T (Y976F) mutation was detected at a rate of 30.8% (4/13) in 2008 [45], decreasing to 3.3% [45] to 7.1% [46] in 2012, and was not detected again after 2015 [45, 46]. So far, the factors that have caused the reduction and disappearance of the c.2927A>T (Y976F) mutation is still unclear. However, given that the c.2927A>T (Y976F) mutation is considered to be associated with the failure of CQ monotherapy for vivax malaria patients [35], it may be prudent to consider that CQ is still a reasonable option for treatment of the *P. vivax* population of Myanmar, as the c.2927A>T (Y976F) mutation is not detected in this specific population.

In this study, the He obtained from 624 *pvmdr1* gene sequences was 0.9515, within the interval of (0.869–0.983 vs. 0.879–0.974) [46] calculated by other scholars for *P. vivax* strains in the China–Myanmar border region. However, a total of 105 haplotypes were more than the 75 [46] species that had been previously identified in the China–Myanmar border region by other scholars in 2015, and the 10 [61] to 27 [33] species found in the South-North Amazon Basin, the North Coast of Peru, and India. In terms of haplotype species, except for the seven haplotypes including threefold mutations (G698S/M908L/T958M) [51, 62], fourfold mutations (G698S/M908L/T958M/F1076L) [30, 51], fivefold mutations (G698S/L845F/M908L/T958M/F1076L) [30, 51] and (G698S/M908L/T958M/Y976F/F1076L) [51], sixfold mutations (K44K/G698S/L845F/M908L/T958M/F1076L) and (T529T/G698S/L845F/M908L/T958M/F1076L) [63], and eightfold mutation (L493L/T529T/G698S/L845F/M908L/T958M/F1076L/E1233E) [63]. the remaining 98 haplotypes had been not previously reported; furthermore, unprecedented ninefold and tenfold mutant haplotypes were also detected. These differences may be due to the fact that more and longer *pvmdr1* sequences were analysed in this study.

Although 105 haplotypes were identified in this study, there was a trend of gradual decrease in the number of haplotypes detected annually, from 88 in 2014 to 15 in 2020, then 21 in 2021, and most recently only 13 in 2022, with low frequency haplotypes being more common only in the early years, particularly 2014 (Table 5; Fig. 3; Additional file 7). In contrast, the mutability of haplotypes shifted towards increasingly complex types, with threefold mutant haplotypes and fourfold mutant haplotypes mostly detected in 2014 and, in recent years, an increase in haplotypes with 10–13-fold loci mutations, such as Hap_40, Hap_91, Hap_2 (Fig. 3). This concentration of joint mutations in a small number of types has facilitated the generation of relatively uniform management strategies, but new adaptive strategies are necessary in order to deal with the problem of multiple mutations.

It is important to mention that in this study, an unexpected occurrence was observed in the description of the *pvmdr1* whole gene sequence, such that the fold of multiple loci mutations obtained from the sequence alignment was inconsistent with those identified by the network evolutionary analysis, where only 15 haplotypes (14.3%, 15/105) remained consistent with the multi-locus joint mutations identified by both methods, whereas the remaining haplotypes (85.7%, 90/105) always experienced more multiple mutations in the network evolutionary analysis (Additional file 7). The reason is that reverse mutations occurring 2–4 times at nine loci, including c.132, c.1477, c.1539, c.1559, c.1587, c.2092, c.3226, c.4074 and c.4179, could not be identified in sequence alignment, which makes it more difficult to accurately describe the sequence diversity of the *pvmdr1* gene. However, this study can provide readers with a better understanding of the polymorphism of the *pvmdr1* gene, and thus is valuable to the public.

In this study, 12 SNPs were found during the TMD of *Pv*MDR1, with only one deleterious mutation c.2533C > T (L845F), and 37 SNPs were found within the digestive vesicle (Additional file 10), with one deleterious mutation c.4179G>C (K1393N) identified in 2.7% (1/37) of samples. PROVEAN and SIFT analyses suggest that these deleterious mutations may lead to altered amino acid charge and hydrophobicity, resulting in a lack of protein structural integrity [46]. Additionally, high-frequency mutations were observed, including c.1539T>A (S513R) (18.9%, 118/624) and c.4349C>T (S1450L) (15.4%, 96/624) within the digestive vesicle and c.3226T>C (F1076L) (75.6%, 472/624) at TMD domain (Additional file 10), which could also affect protein function based on previous research [47, 64].

In this study, the prevalent trend of the molecular markers associated with drug resistance in *P. vivax* strains infected with vivax malaria cases in Yunnan Province are systematically revealed, and a set of *pvmdr1* full gene sequencing of *P. vivax* strains from the Myanmar population were obtained in batch, which will provide valuable information and enrich the GenBank data. However, there are some limitations to this study. Firstly, the copy number of *pvmdr1* genes was not assessed. Secondly, due to limitation on the length of the article, the others molecular markers for drug resistance monitoring other than *pvmdr1* were not included in the analyses. Future research should conduct a study on the association between resistance molecular markers and anti-malarial drug susceptibility phenotypes, as well as improving the monitoring data on molecular markers for drug resistance of *P. vivax*.

## Conclusion

Most *P. vivax* strains of vivax malaria infections in Yunnan Province had been highly mutated in *pvmdr1* gene, with variations in predominant mutation type from year to year. In recent years, more of the five to sevenfold mutation haplotypes have been detected, but with fewer deleterious mutation loci. It is worth further exploring the dynamics of *pvmdr1* mutation polymorphism accumulation and reasonably verifying the correlation between the special mutation of *pvmdr1* gene and the phenotypic changes in the susceptibility of anti-malarial drugs for treating vivax malaria, such as CQ.

## Supplementary Information

**Additional file 1: ** Confirmation of malaria case infected with mono-*Plasmodium vivax* in Yunnan Province.

**Additional file 2: **Using NC_009915.1 reference sequence as the template of *pvmdr1 *gene for designment the different primers.

**Additional file 3: ** The distribution of vivax malaria cases diagnosed in different years and prefectures.

**Additional file 4:** Electrophoresis of PCR amplification products of *pvmdr1* gene in *P. vivax *from vivax malaria cases in Yunnan Province.

**Additional file 5: ** Identify true base substitutions.

**Additional file 6:** **Fig. S1** The subfigure of SNPs composition at 2014, **Fig. S2** The subfigure of SNPs composition at 2020, **Fig. S3** The subfigure of SNPs composition at 2021 and **Fig. S4** Subfigure of SNPs composition at 2022, respectively.

**Additional file 7.** The composition of 105 haplotypes identified from 624 pvmdr1 gene sequences.

**Additional file 8: Fig.S1** The subfigure of haplotypes composition at 2014, **Fig. S2 **The subfigure of haplotypes composition at 2020, **Fig. S3** The subfigure of haplotypes composition at 2021, **Fig. S4** The subfigure of haplotypes composition at 2022, respectively.

**Additional file 9.** The difference of multiplicity degree in 90 haplotypes identified by between gene sequence alignment and network evolutionary analysis.

**Additional file 10.** The predicted 3D structural diagram of P. vivax multidrug resistance protein 1 (PvMDR1).

## Data Availability

Not applicable.

## Abbreviations

CQ: Chloroquine
WHO: World Health Organization
DRC: Democratic Republic of the Congo
*P. falciparum*: *Plasmodium falciparum*
*P. vivax*: *Plasmodium vivax*
IC50: 50% Inhibition concentration
AQ: Amodiaquine
SP: Sulfadoxine-pyrimethamine
*pvdhfr*: *Plasmodium vivax* Dihydrofolate reductase gene
*pvdhps*: *Plasmodium vivax* Dihydropteroate synthase gene
*pvmdr1*: *Plasmodium vivax* Multidrug resistance 1 protein gene
YPMDRL: Yunnan Province Malaria Diagnosis Referent Laboratory
CDSIRS: China Disease Surveillance Information Reporting System
DBD: Dried blood dots
CDS: Coding DNA sequences
SNP: Single nucleotide polymorphism
π: Nucleotide diversity
He: Heterozygosity
*Pv*MDR1: *Plasmodium vivax* Multidrug resistance protein 1
χ^2^: Chi-square test
MAF: Minor allele frequency
OD: Order
TMD: Transmembrane domain
AL: Alleles
AAV: Coding amino acid variation
IP: Imported
ID: Indigenous
PROVEAN: Protein variation effect analyzer
SIFT: Sorting intolerant from tolerant
