# Supplementary material for: Characteristics of molecular markers associated with chloroquine resistance in Plasmodium vivax strains from vivax malaria cases in Yunnan Province, China
Source: Malar J. 2023 Jun 11;22:181. doi: 10.1186/s12936-023-04616-0 (PMC10257827; doi:10.1186/s12936-023-04616-0)
Supplement: Supplementary file 1 — Additional file 1: Confirmation of malaria case infected with mono-Plasmodium vivax in Yunnan Province. [file 12936_2023_4616_MOESM1_ESM.doc]

**Additional file 1**

**Confirmation of malaria case infected with mono-*Plasmodium vivax* in Yunnan Province**

**Method**

When the febrile patients were found to infect *Plasmodium* by microscope examination in county-level laboratory, these objects were initially diagnosed as malaria cases, their blood samples would be sent to the Yunnan Province Malaria Diagnostic Reference Laboratory (YPMDRL) for re-testing again both by microscopic examination and by genetic testing. The method of genetic testing was introduced by Snounou et al ^[1]^. The details of nested PCR testing for differentiating between various *Plasmodium* species were in Table 1 as following:

**Results**

All of 753 vivax malaria cases were re-tested by YPMDRL using both microscopic examination and nest-PCR, the *Plasmodium* pictures and electrophoresis diagram of PCR products from every cases blood samples was shown for the examples in Fig. 1 and Fig. 2. Finally, they were identified as mono-*P. vivax* infection.

**References**

1. Snounou G, Viriyakosol S, Zhu XP, Jarra W, Pinheiro L, do Rosario VE, et al. High sensitivity of detection of human malaria parasites by the use of nested polymerase chain reaction. Mol Biochem Parasitol. 1993;61:315–320.

| **Table S1 The details of nested PCR testing for differentiating between various *Plasmodium* species** | | | | | | |
| --- | --- | --- | --- | --- | --- | --- |
| Nested PCR | Specificity of primers | Primer name^△^ | Primer sequence^△^ | Expected PCR product (bp) | Reaction conditions | Reaction systems |
| First round | Genus | rPLU5 | 5’-CCTGTTGTTGCCTTAAACTTC-3； | 1200 | 94 ° C for 3min;94° C for 30s, 58° C for 30s, 72° C for 60s, 34 cycles; 72° C for 5min. | 25 ul reaction volume including 2.6 μl template, 14.0 μl 2 × PCR Mix hybrid system (Containing Taq enzyme), 0.7 μl upstream primer (20umol / L) and 0.7 μl downstream primers (20umol / L) |
|  |  | rPLU6 | 5’-TTAAAATTGTTGCAGTTAAAACG-3’ |  |  |  |
| Second round | *P.falciparum* | rFAL1 | 5’-TTAAACTGGTTTGGGAAAACCAAATATATT-3’ | 205 | 94 ° C for 3min;94° C for 30s, 60° C for 30s, 72° C for 60s, 34 cycles; 72° C for 5min |  |
|  |  | rFAL2 | 5’-ACACAATGAACTCAATCATGACTACCCGTC-3’ |  |  |  |
|  | *P.vivax* | rVIV1 | 5’-CGCTTCTAGCTTAATCCACATAACTGATAC-3’ | 120 |  |  |
|  |  | rVIV2 | 5’-ACTTCCAAGCCGAAGCAAAGAAAGTCCTTA-3’ |  |  |  |
|  | *P.malariae* | rMAL1 | 5’-ATAACATAGTTGTACGTTAAGAATAACCGC-3’ | 141 |  |  |
|  |  | rMAL2 | 5’-AAAATTCCCATGCATAAAAAATTATACAAA-3’ |  |  |  |
|  | *P.ovale* | rOVA1 | 5’-ATCTCTTTTGCTATTTTTTAGTATTGGAGA-3’ | 800 |  |  |
|  |  | rOVA2 | 5’-GGAAAGGACACATTAATTGTATCCTAGTG-3’ |  |  |  |
| ^△^:The primers’ name and sequence were cited from References Snounou G, Viriyakosol S, Zhu XP, Jarra W, Pinheiro L, do Rosario VE, et al. High sensitivity of detection of human malaria parasites by the use of nested polymerase chain reaction. Mol Biochem Parasitol. 1993;61:315-320. | | | | | | |


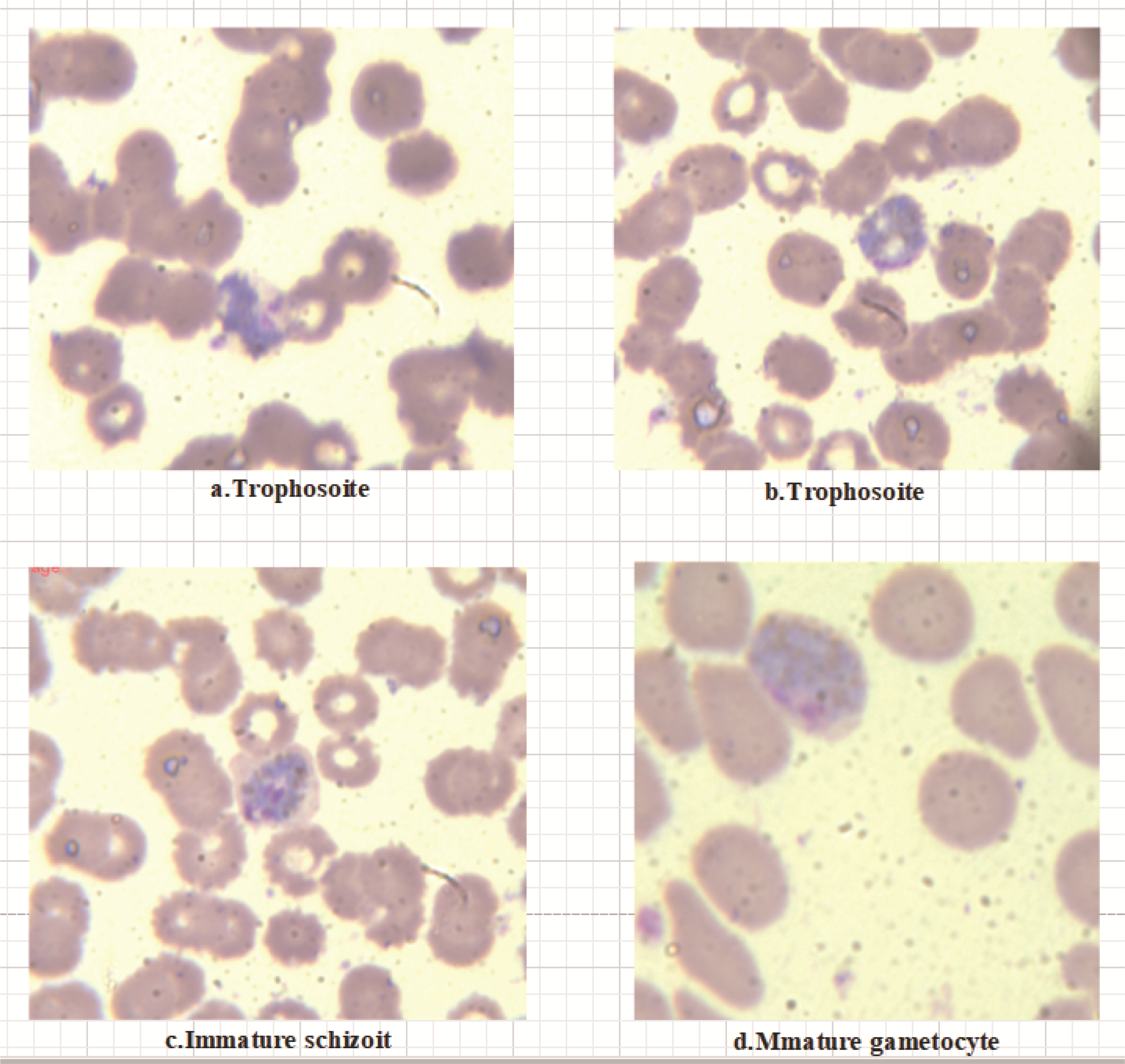


**Fig. S1 The pictures of *P. vivax* by microspopic examination**


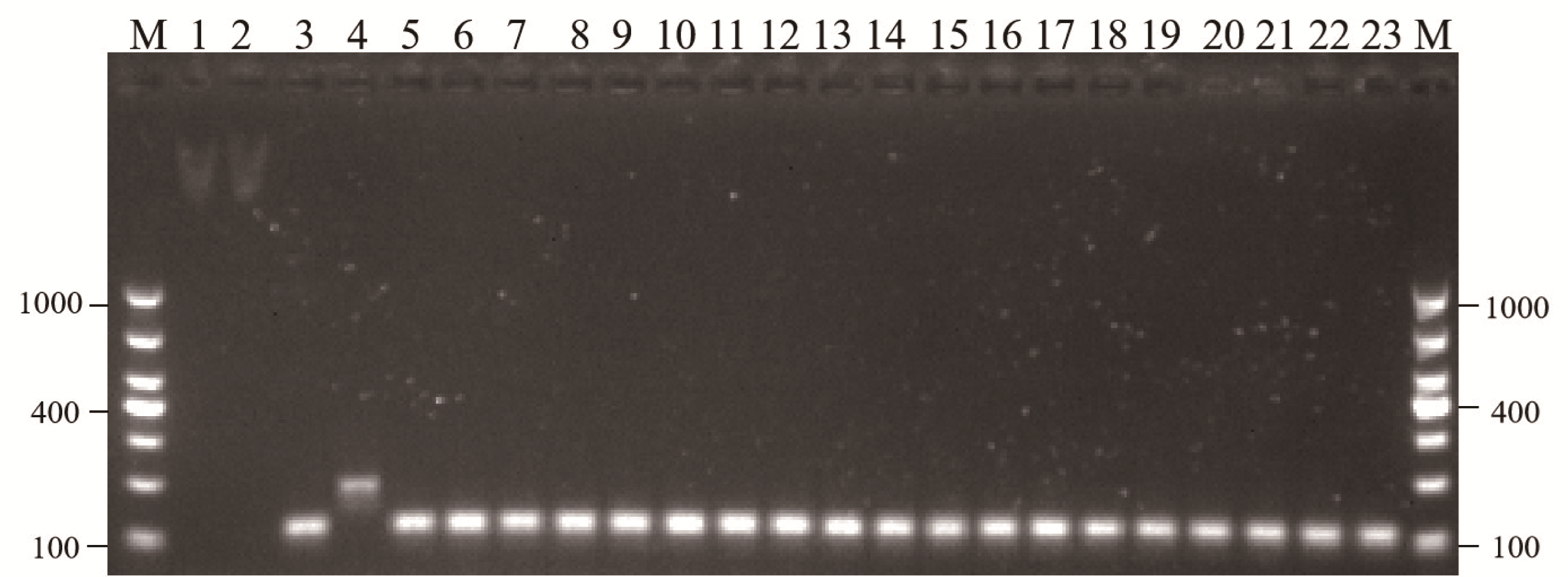


(1) M: DNA marker; (2) 1: Negative control of the primary PCR; 2: Negative control of the second round-PCR; 3: *P. vivax* positive control; 4: *P. falciparum* positive control; 5, 6, 7, 8, 9, 10, 11, 12, 13, 14, 15, 16, 17, 18, 19, 20, 21, 22, 23: Plasmodium vivax infection sample .

**Fig. S2 Electrophoresis of PCR amplification products of 18 ssRNA gene in *P. vivax***
