# Supplementary material for: Characteristics of molecular markers associated with chloroquine resistance in Plasmodium vivax strains from vivax malaria cases in Yunnan Province, China
Source: Malar J. 2023 Jun 11;22:181. doi: 10.1186/s12936-023-04616-0 (PMC10257827; doi:10.1186/s12936-023-04616-0)
Supplement: Supplementary file 2 — Additional file 2: Using NC_009915.1 reference sequence as the template of pvmdr1 gene for designment the different primers. [file 12936_2023_4616_MOESM2_ESM.doc]

**Additional file 2**

**Using NC_009915.1 reference sequence as the template of *pvmdr1* gene for designment the different primers**


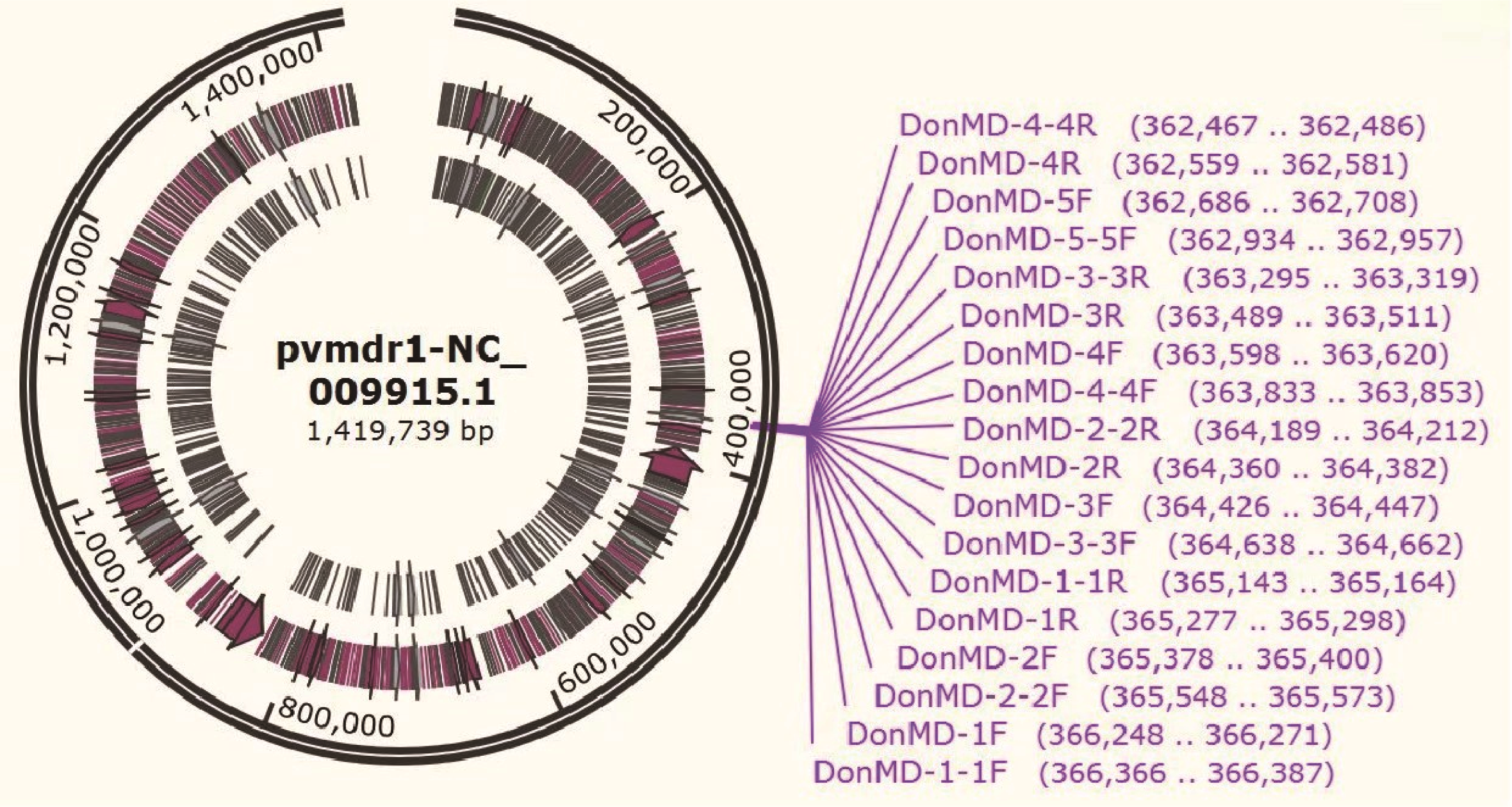


**Fig. S1 The regions and primer names of PCR amplification for *pvmdr1* gene in *P. vivax***
