# Supplementary material for: Characteristics of molecular markers associated with chloroquine resistance in Plasmodium vivax strains from vivax malaria cases in Yunnan Province, China
Source: Malar J. 2023 Jun 11;22:181. doi: 10.1186/s12936-023-04616-0 (PMC10257827; doi:10.1186/s12936-023-04616-0)
Supplement: Supplementary file 3 — Additional file 3: The distribution of vivax malaria cases diagnosed in different years and prefectures. [file 12936_2023_4616_MOESM3_ESM.docx]

**Additional file 3**

**The distribution of vivax malaria cases diagnosed in different years and prefectures**

| **Table 1 The prefecture distribution of 753 vivax malaria cases diagnosed in Yunnan Province from January to December 2014 and from January 2020 to December 2022** | | | | | |
| --- | --- | --- | --- | --- | --- |
| **Prefectures of diagnosis** | **Total**  **(n, F%)** | **2014**  **(n, F%)** | **2020**  **(n, F%)** | **2021**  **(n, F%)** | **2022**  **(n, F%)** |
| **Total** | 753(100%) | 379(50.3%) | 154(20.5%) | 126(16.7%) | 94(12.5%) |
| **Dehong** | 450(59.8%) | 177(46.7%) | 107(69.5%) | 89(70.6%) | 77(81.9%) |
| **Baoshan** | 175(23.2%) | 130(34.3%) | 23(14.9%) | 16(12.7%) | 6(6.4%) |
| **Lincang** | 41(5.4%) | 10(2.6%) | 14(9.1%) | 11(8.7%) | 6(6.4%) |
| **Kunming** | 22(2.9%) | 10(2.6%) | 6(3.9%) | 4(3.1%) | 2(2.1%) |
| **Dali** | 18(2.4%) | 14(3.7%) | 0 | 3(2.4%) | 1(1.1%) |
| **Pu'er** | 14(1.9%) | 14(3.7%) | 0 | 0 | 0 |
| **Nujiang** | 7(0.9%) | 7(1.8%) | 0 | 0 | 0 |
| **Xishuanbanna** | 7(0.9%) | 4(1.1%) | 0 | 1(0.8%) | 2(2.1%) |
| **Honghe** | 5(0.7%) | 1(0.3%) | 2(1.3%) | 2(1.6%) | 0 |
| **Lijiang** | 3(0.4%) | 3(0.8%) | 0 | 0 | 0 |
| **Wenshan** | 3(0.4%) | 3(0.8%) | 0 | 0 | 0 |
| **Qujing** | 2(0.3%) | 1(0.3%) | 1(0.6%) | 0 | 0 |
| **Yuxi** | 2(0.3%) | 2(0.5%) | 0 | 0 | 0 |
| **Chuxiong** | 2(0.3%) | 1(0.3%) | 1(0.6%) | 0 | 0 |
| **Zhaotong** | 1(0.1%) | 1(0.3%) | 0 | 0 | 0 |
| **Diqing** | 1(0.1%) | 1(0.3%) | 0 | 0 | 0 |
