# Supplementary material for: Characteristics of molecular markers associated with chloroquine resistance in Plasmodium vivax strains from vivax malaria cases in Yunnan Province, China
Source: Malar J. 2023 Jun 11;22:181. doi: 10.1186/s12936-023-04616-0 (PMC10257827; doi:10.1186/s12936-023-04616-0)
Supplement: Supplementary file 4 — Additional file 4: Electrophoresis of PCR amplification products of pvmdr1 gene in P. vivax from vivax malaria cases in Yunnan Province. [file 12936_2023_4616_MOESM4_ESM.docx]

**Additional file 4**

**Electrophoresis of PCR amplification products of *pvmdr1* gene in *P. vivax* from vivax malaria cases in Yunnan Province**

**Fig. S1 Electrophoresis of PCR amplification products of *pvmdr1* gene in *P. vivax* strains collected from vivax malaria cases reported by Yunnan Province** (1) M: DNA marker; (2) 1, 6, 11, 16, 20: Blank control of the first round PCR; 2, 7, 12, 17, 21 : Blank control of the second round PCR; 3-5: Amplification products of second round PCR for fragment F1; 8-10: Amplification products of second round PCR for fragment F2; 13-15: Amplification products of second round PCR for fragment F3; 18-19: Amplification products of second round PCR for fragment F4; 22-23: Amplification products of second round PCR for fragment F5.
