# Supplementary material for: Characteristics of molecular markers associated with chloroquine resistance in Plasmodium vivax strains from vivax malaria cases in Yunnan Province, China
Source: Malar J. 2023 Jun 11;22:181. doi: 10.1186/s12936-023-04616-0 (PMC10257827; doi:10.1186/s12936-023-04616-0)

**Additional file 5**

**Identify true base substitutions**

Confirmation of the base substitute from wildtype to mutant type at 52 SNPs of *pvmdr1* gene in *P. vivax* strains by checking sequencing peaks. All checking as following:

f


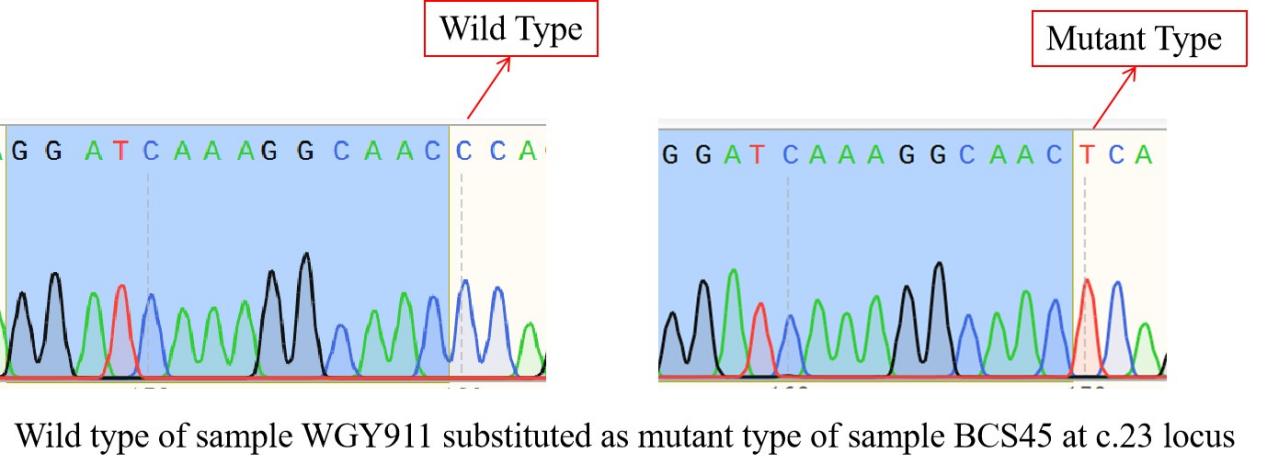


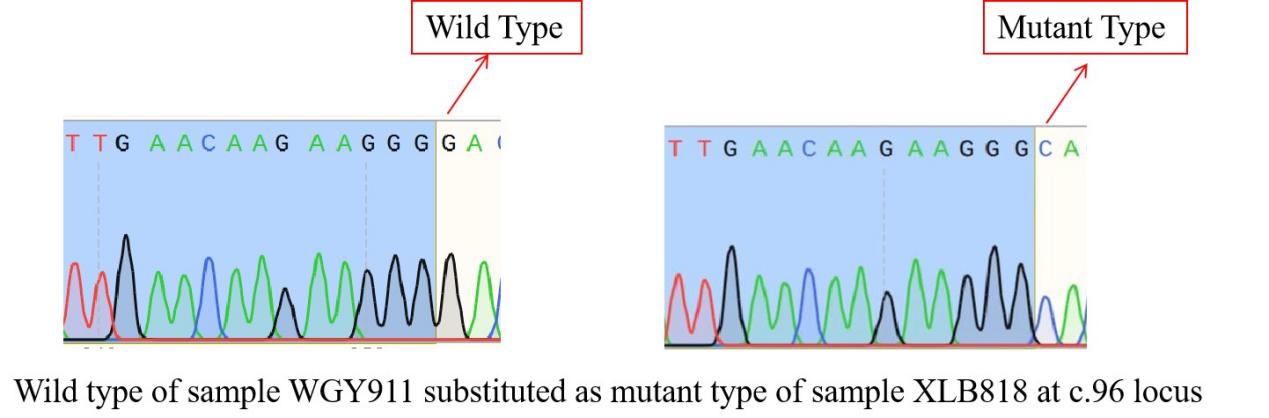


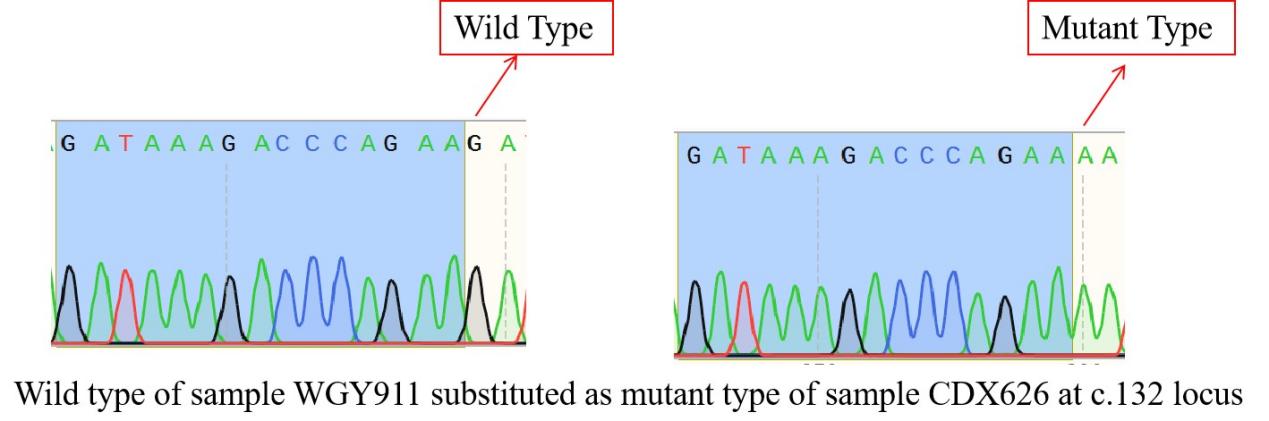


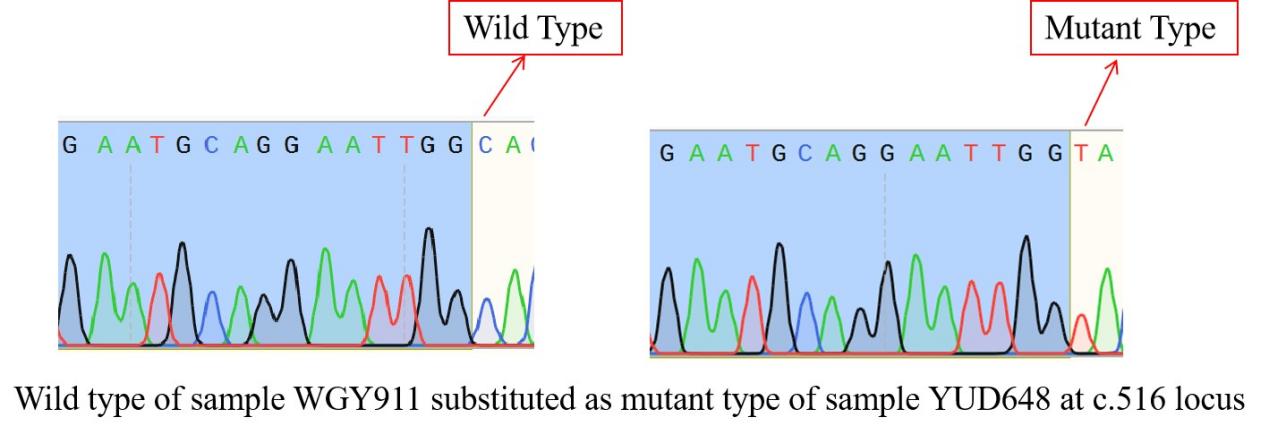


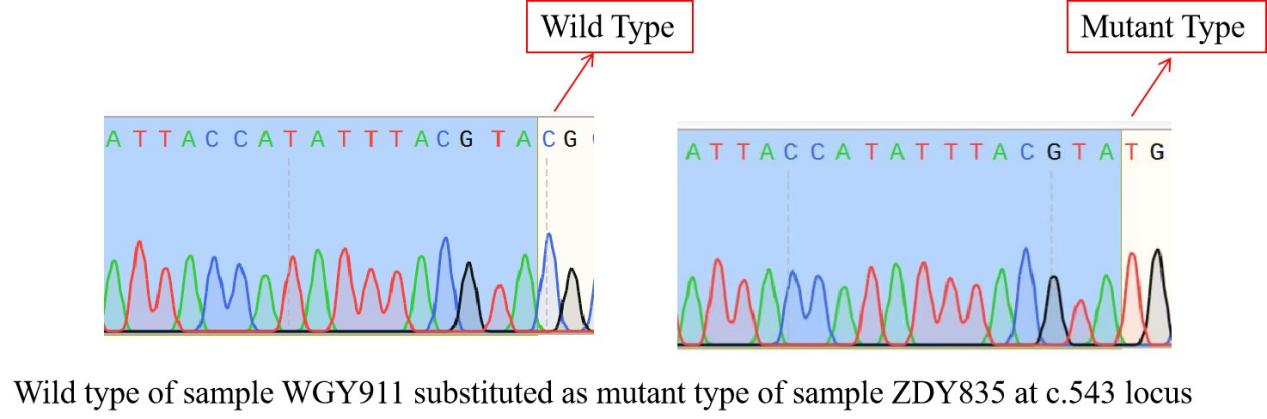


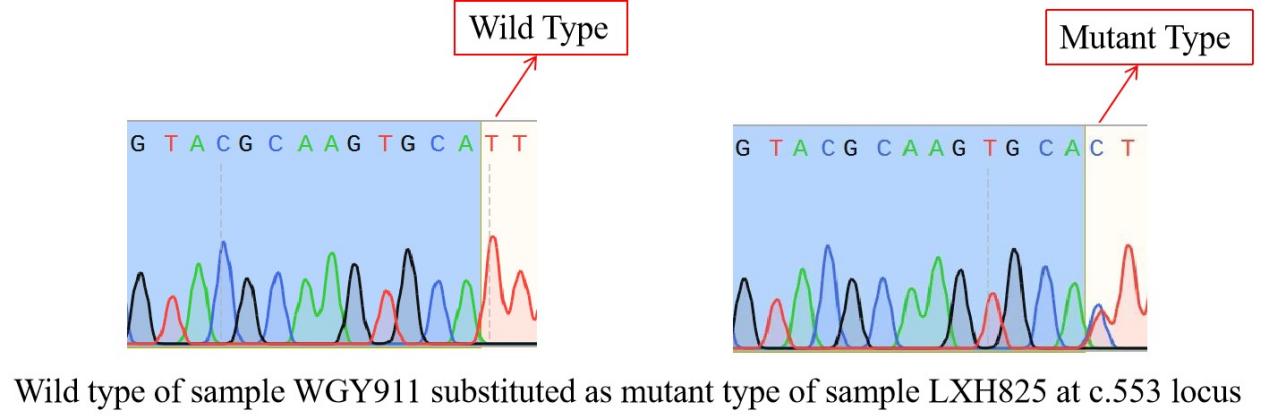


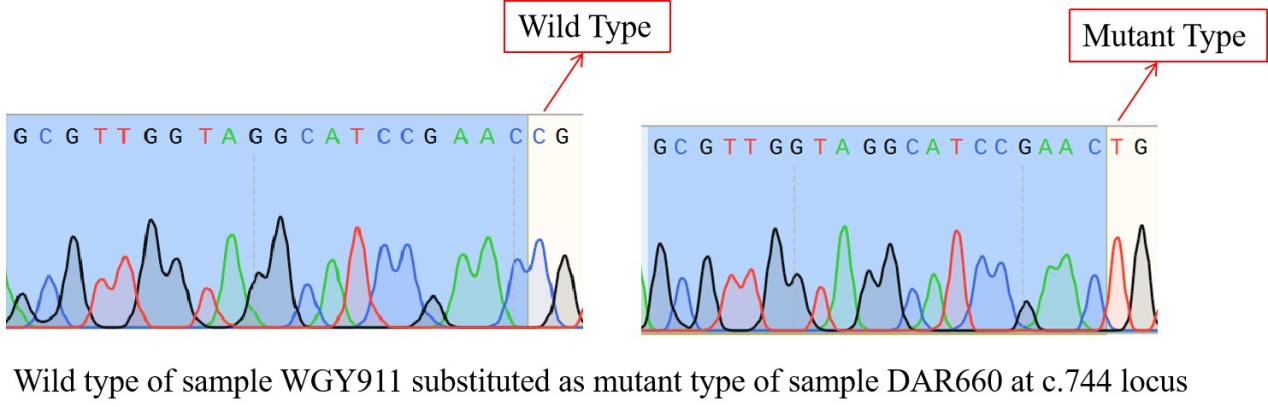


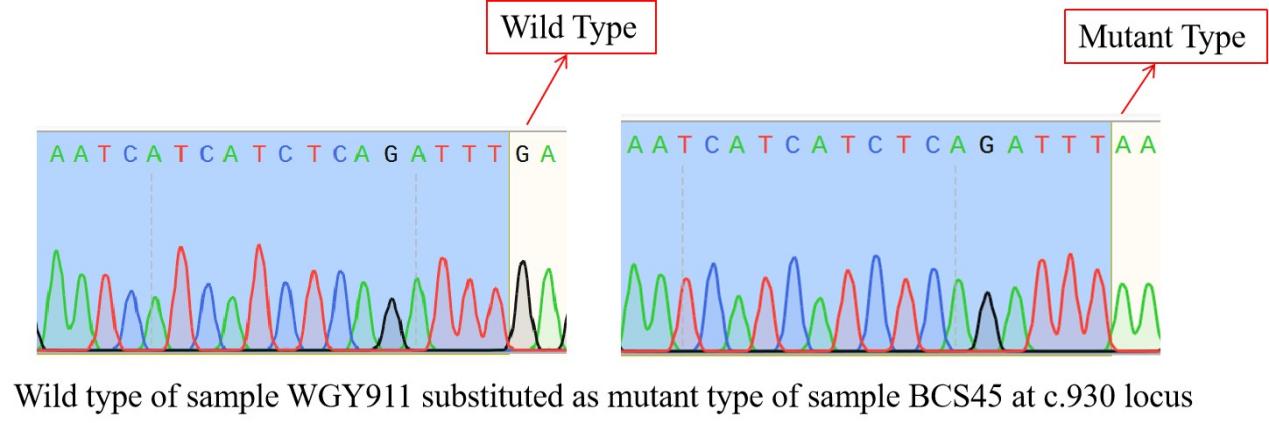

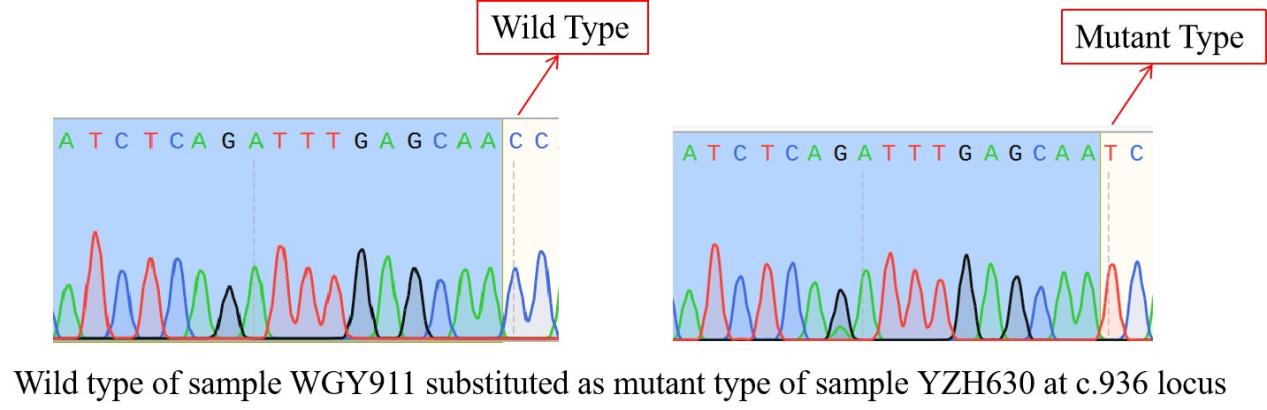


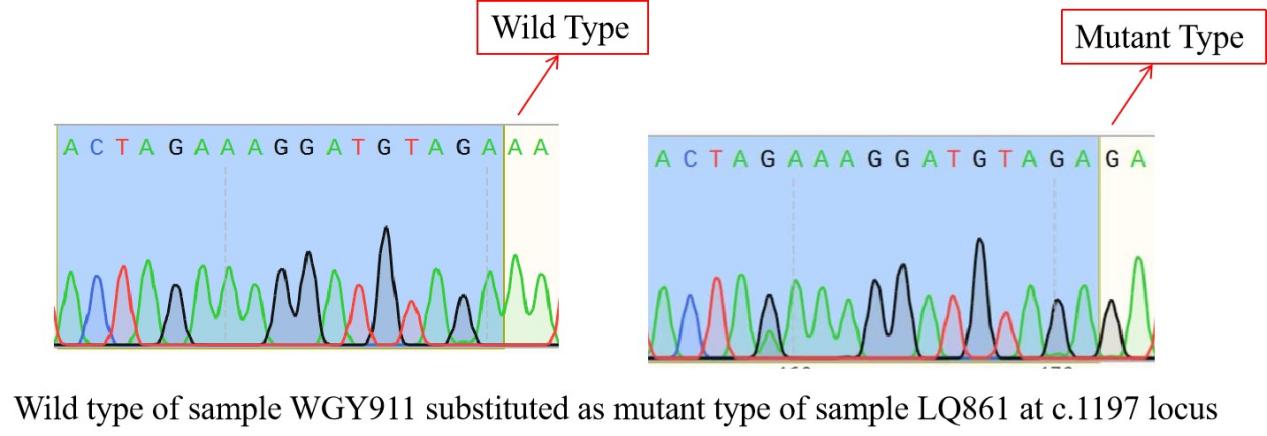


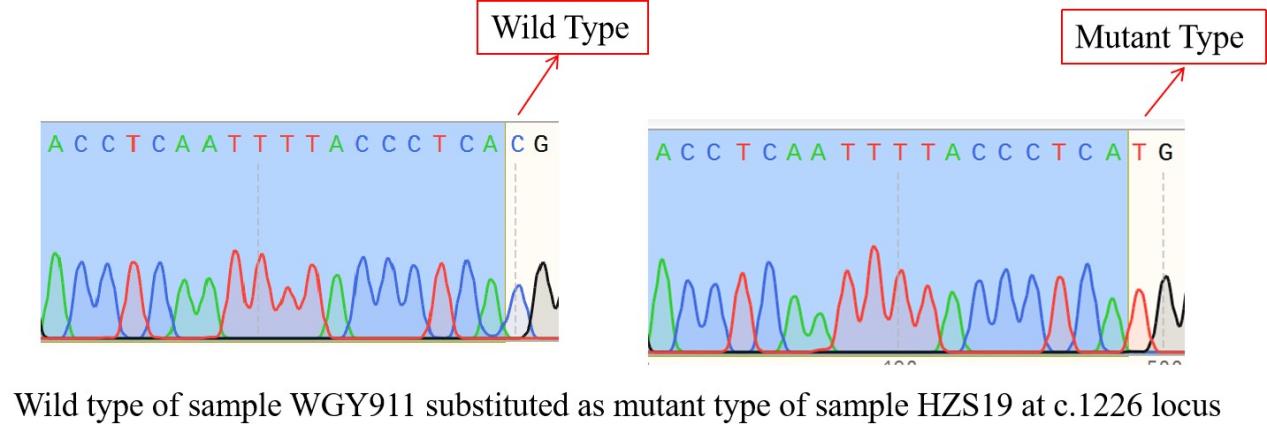


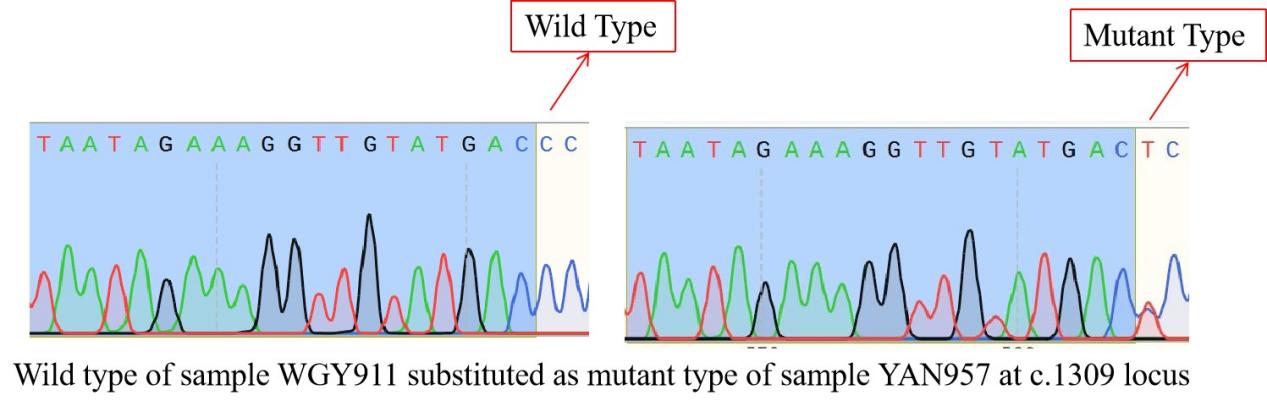

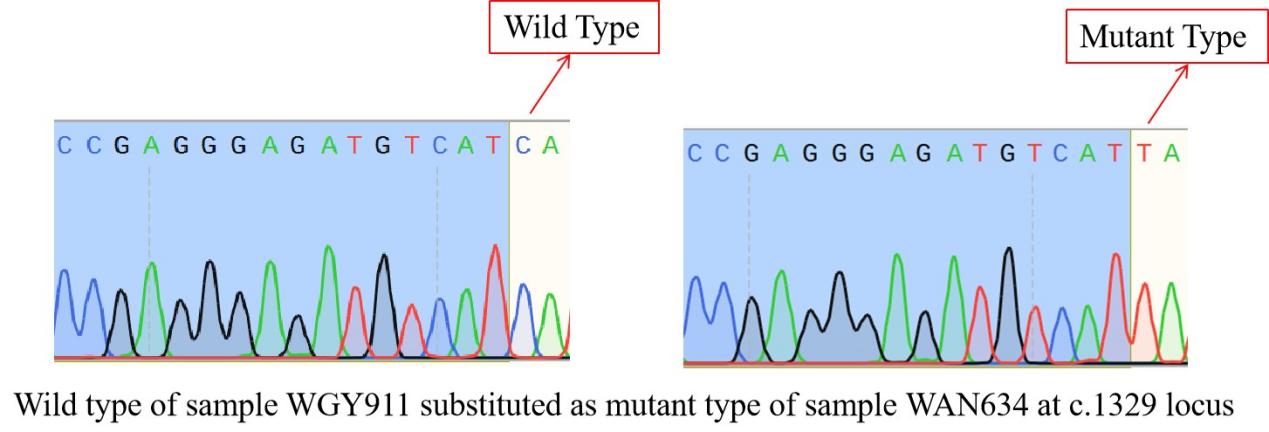


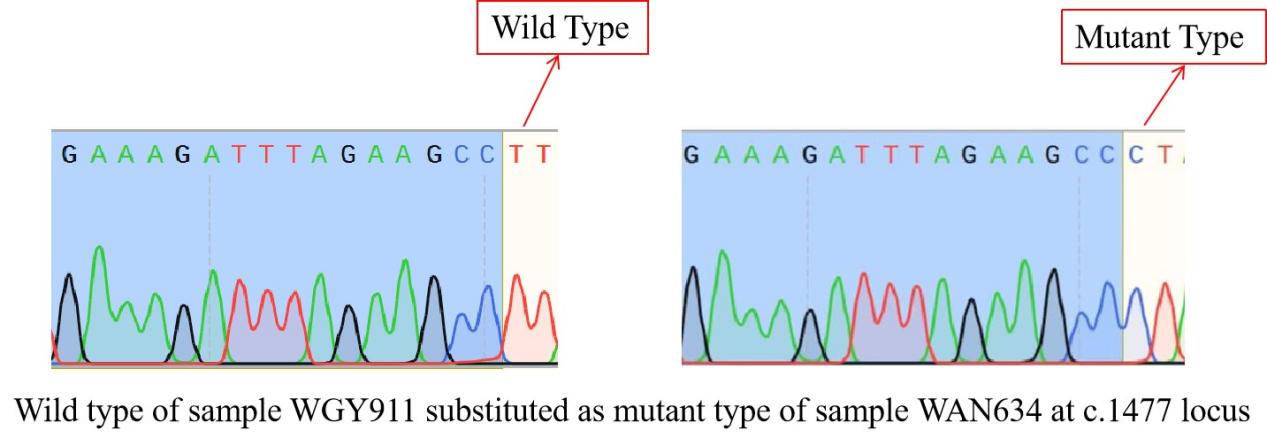


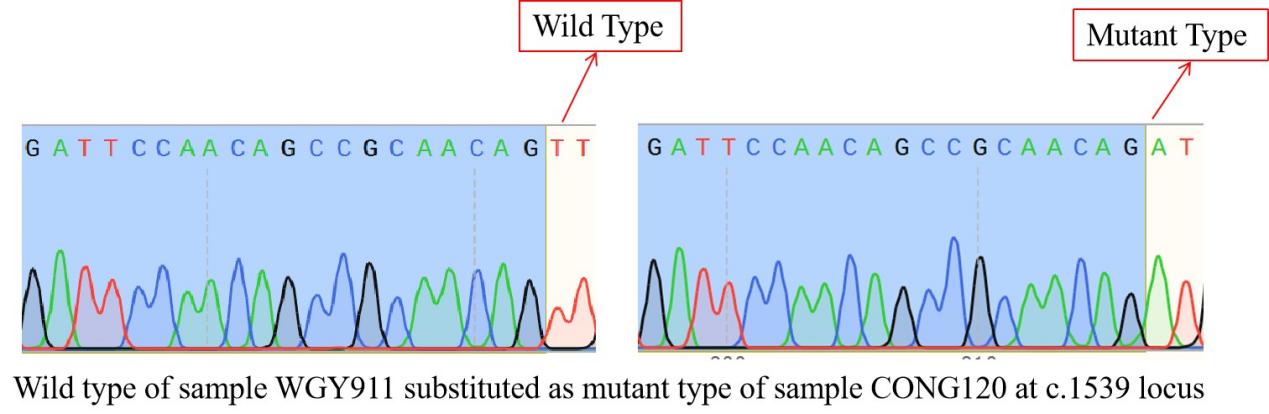


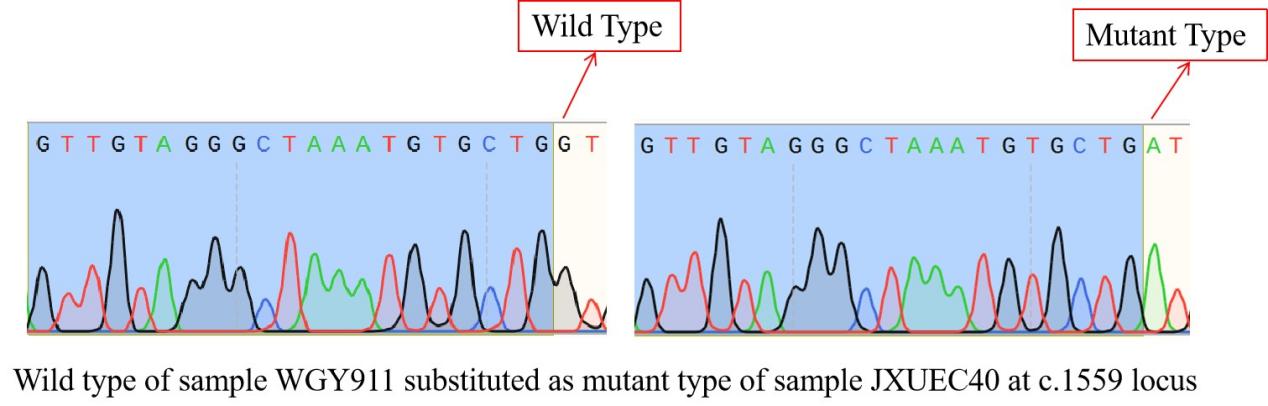

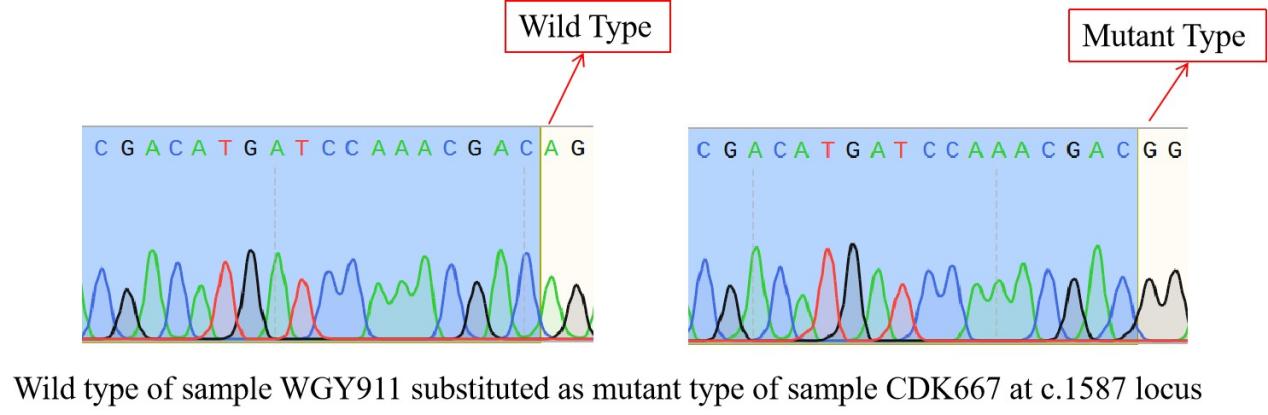


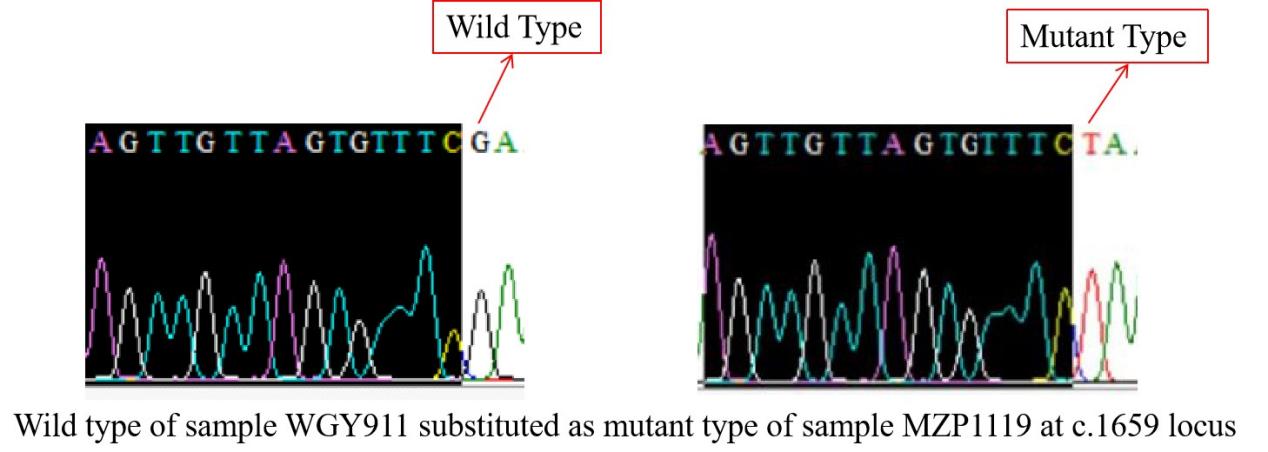


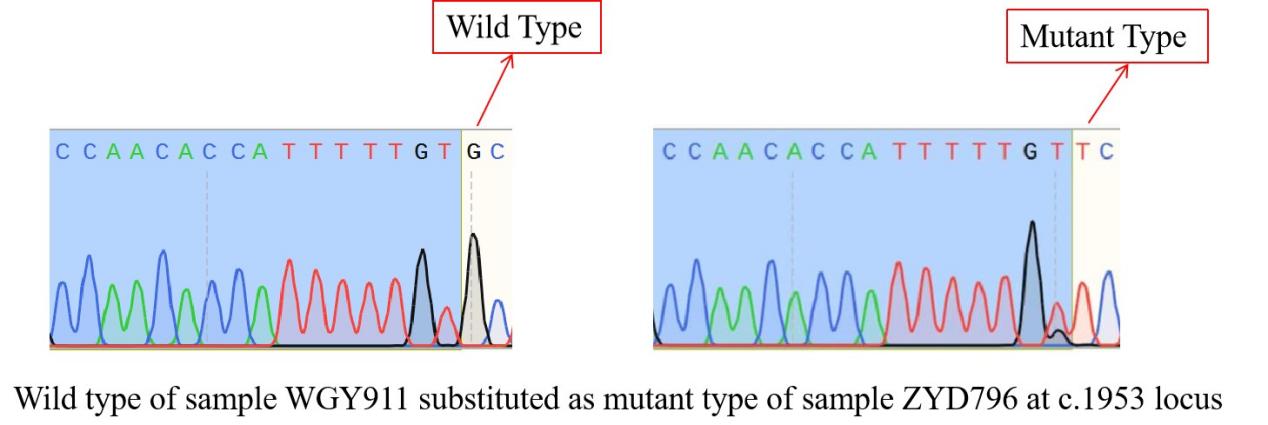


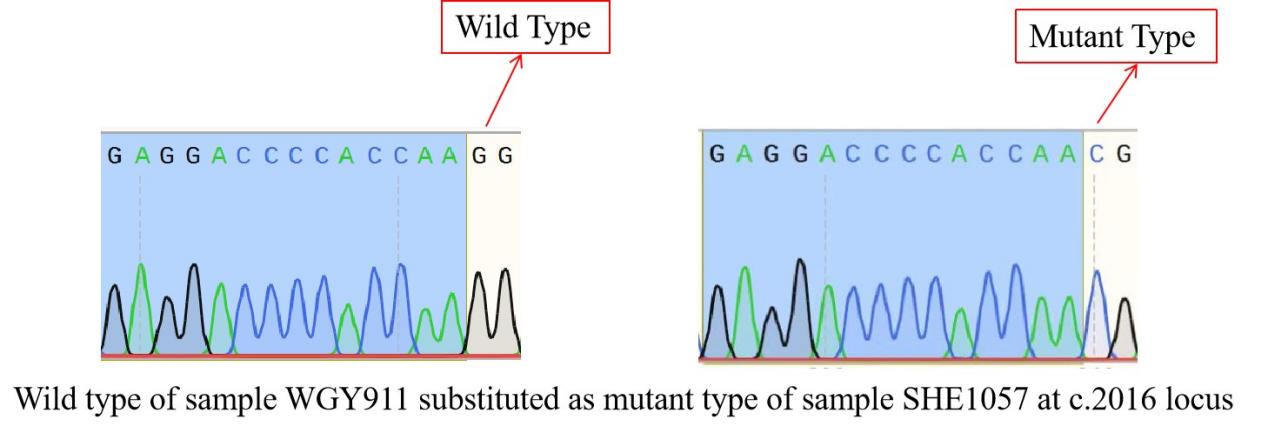

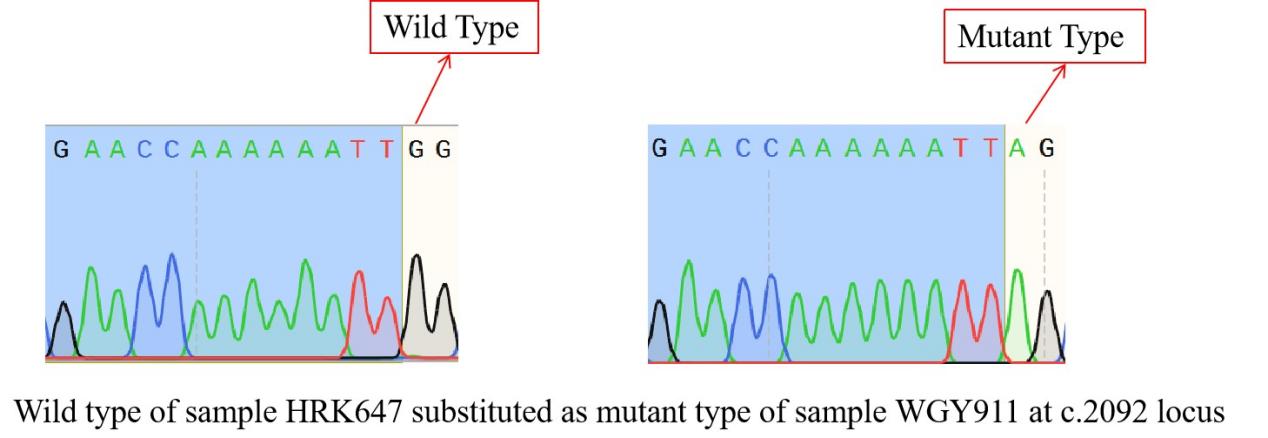


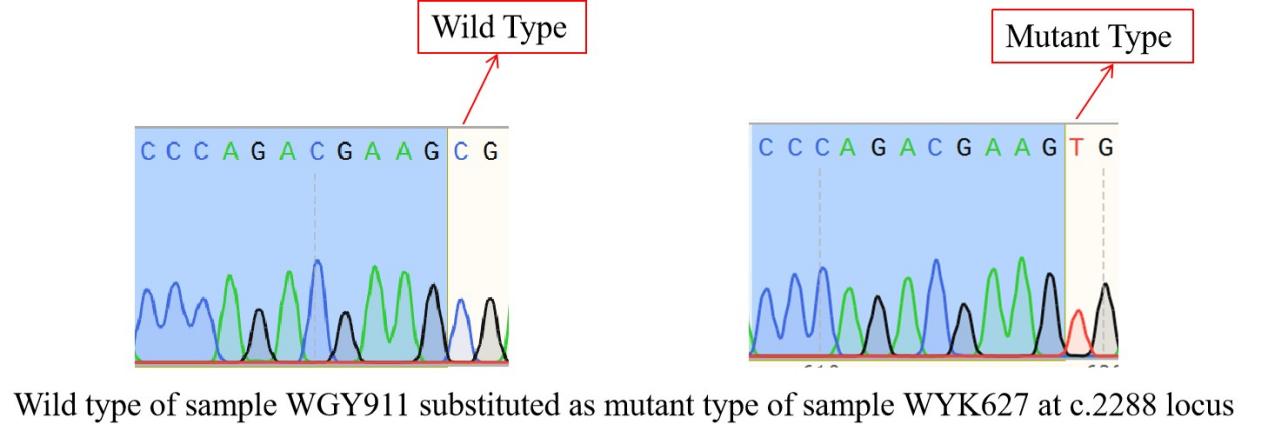


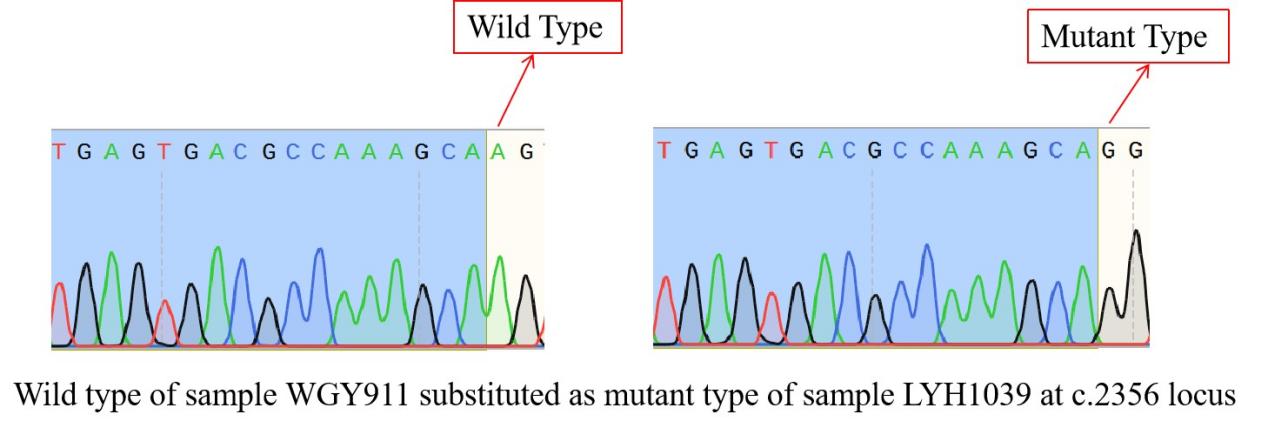


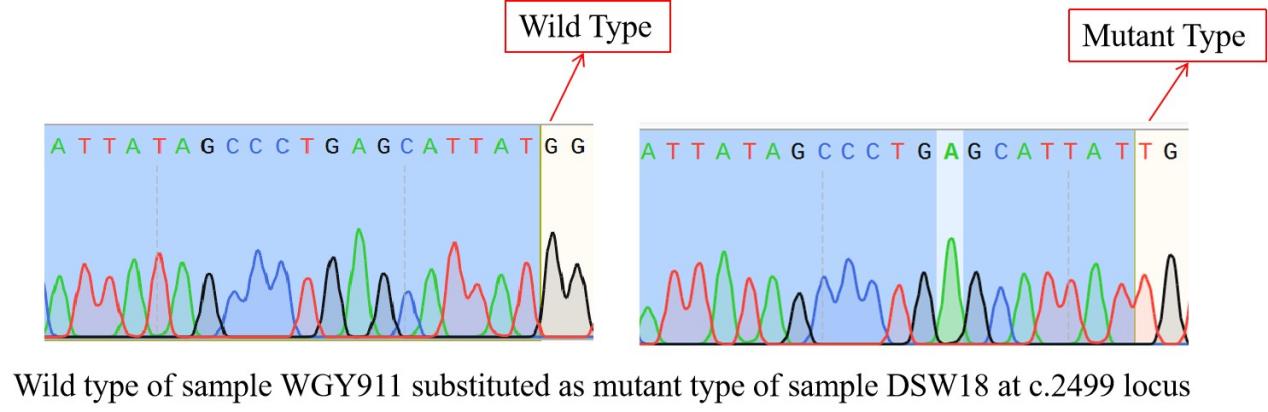

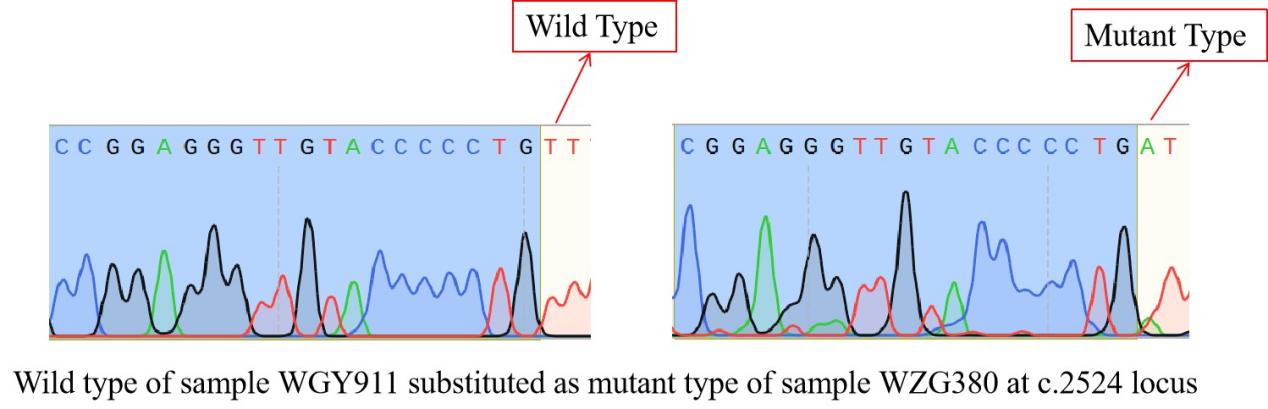


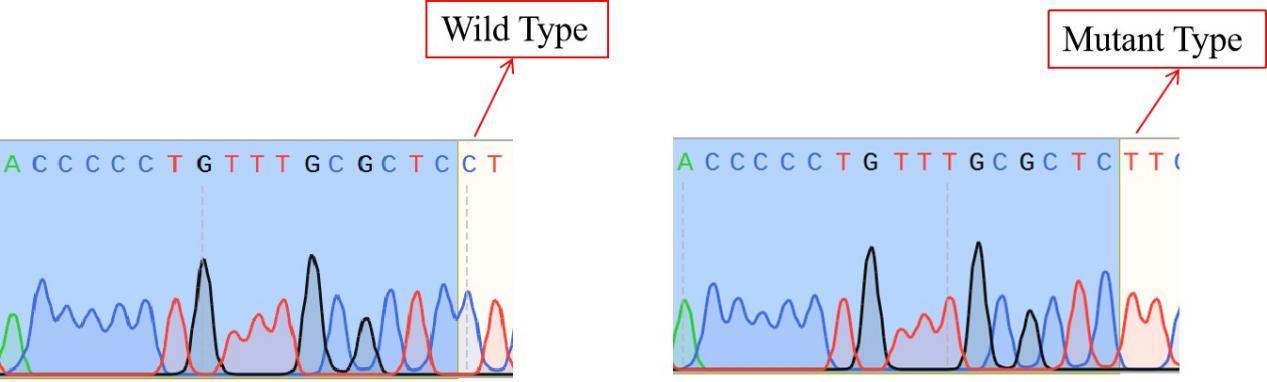


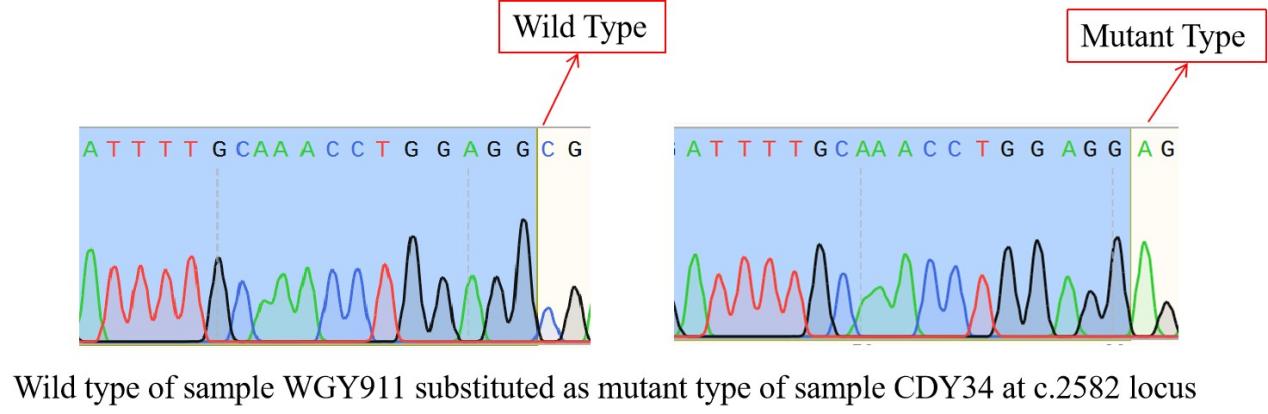


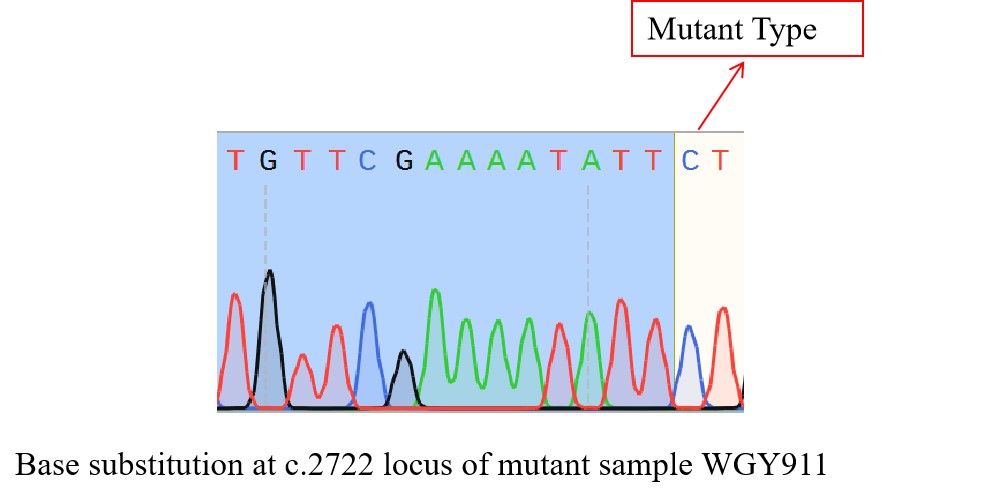


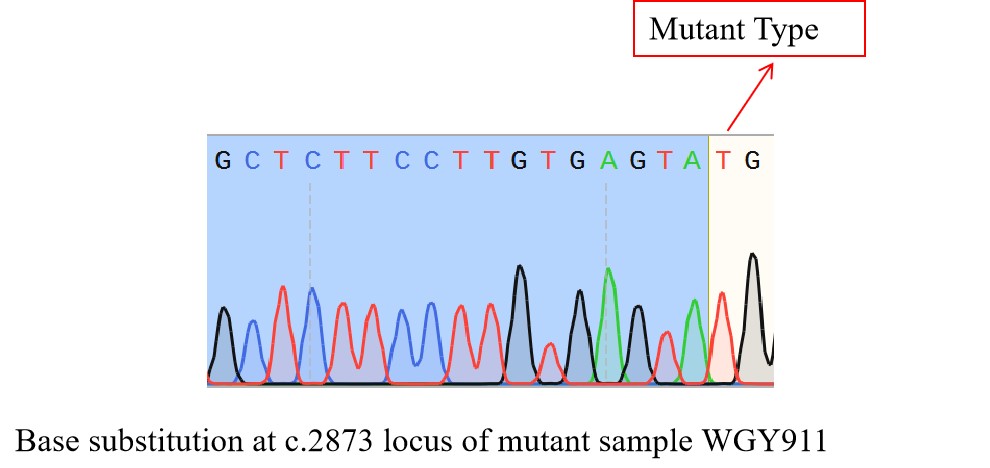


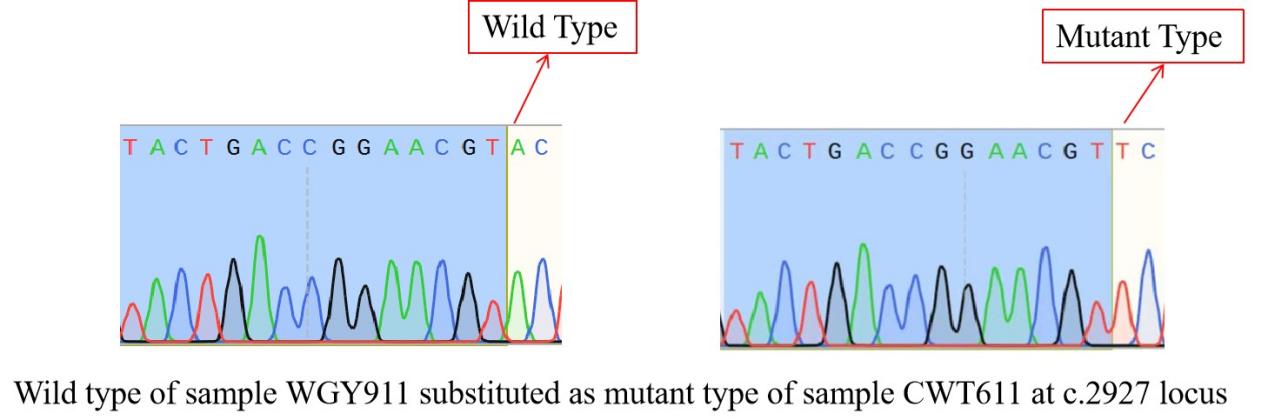


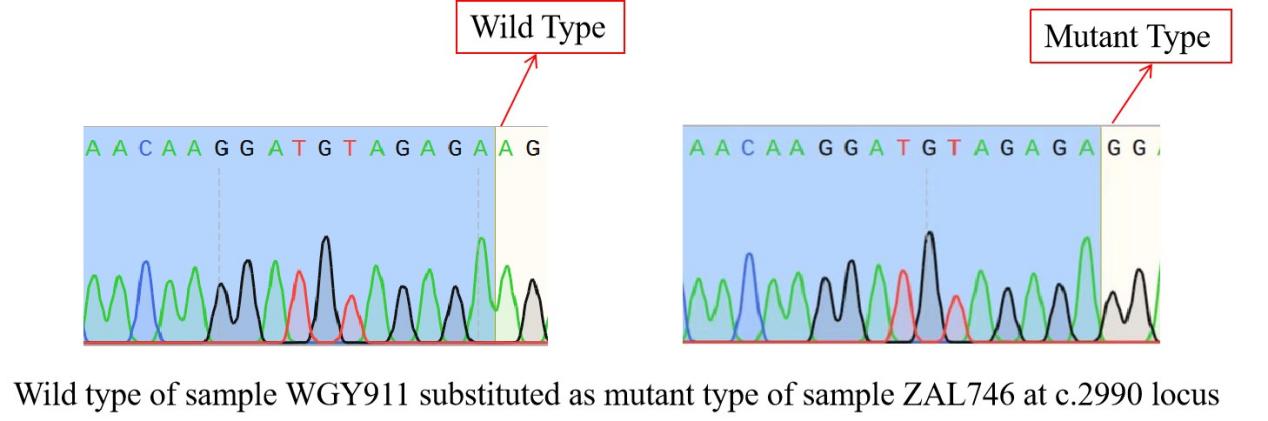


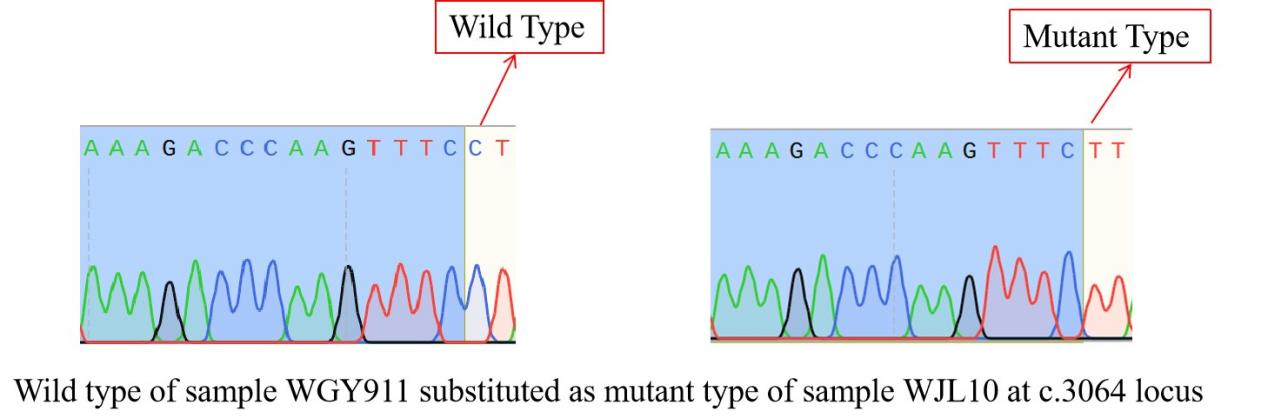

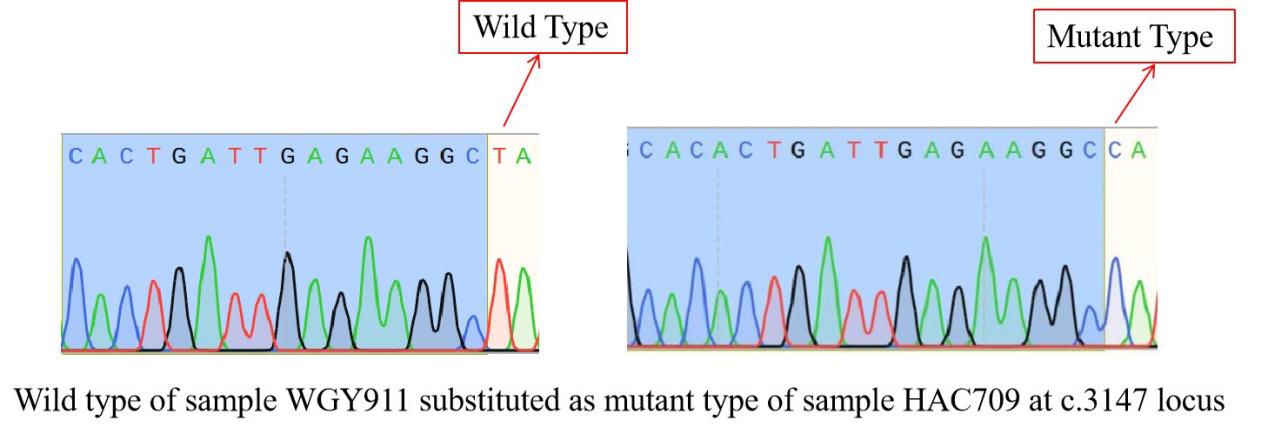


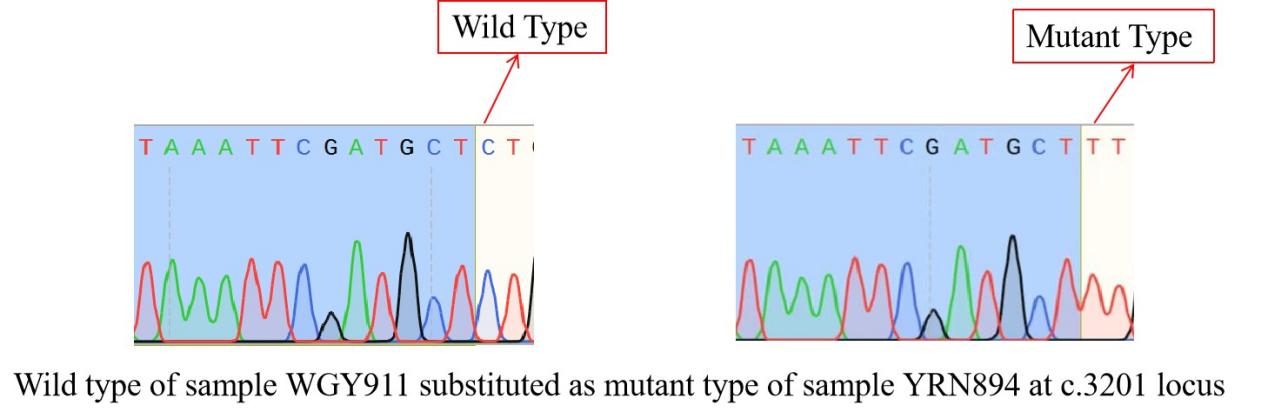


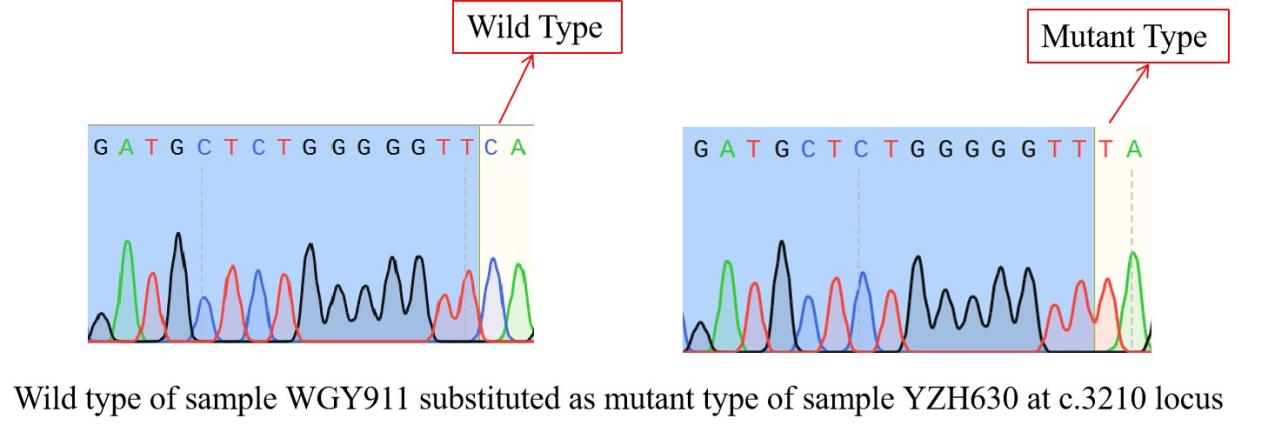


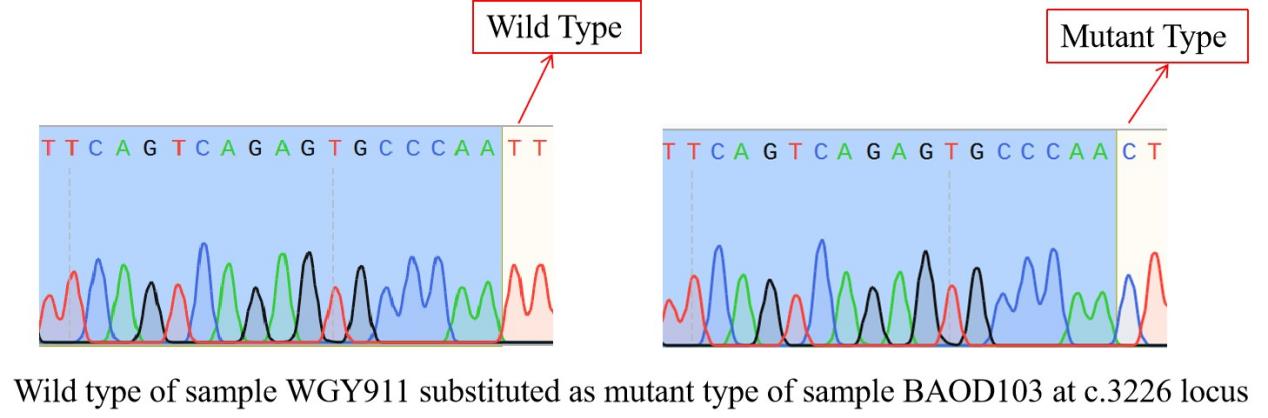

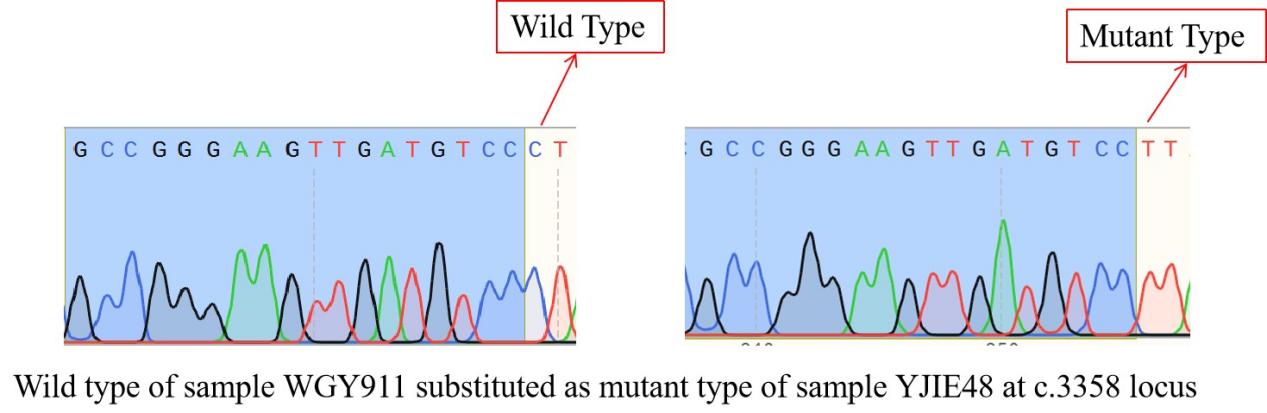


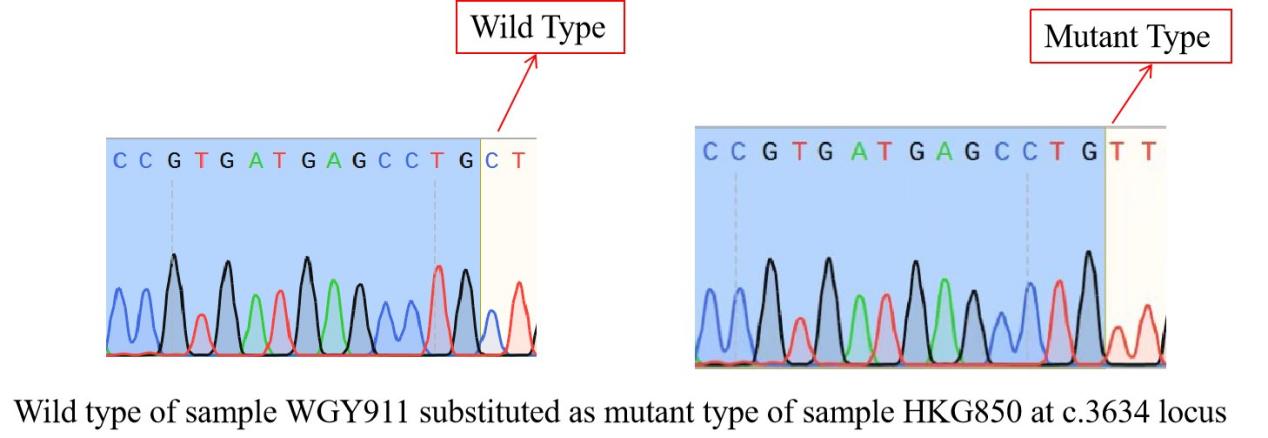


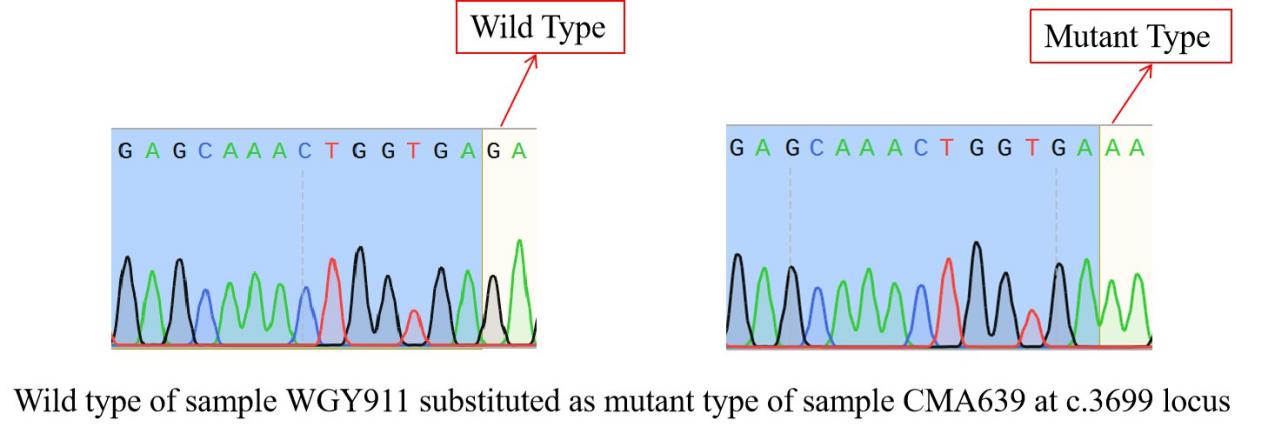


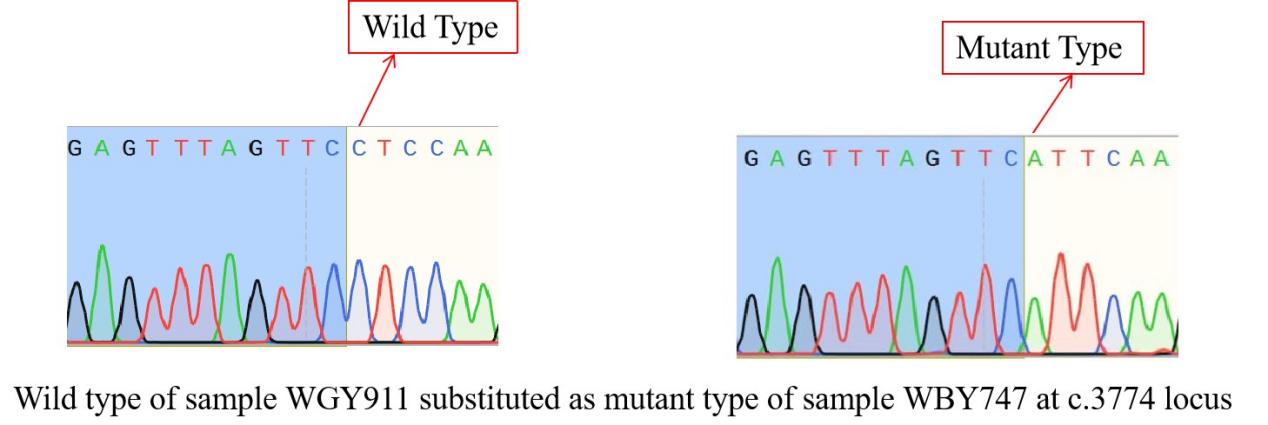

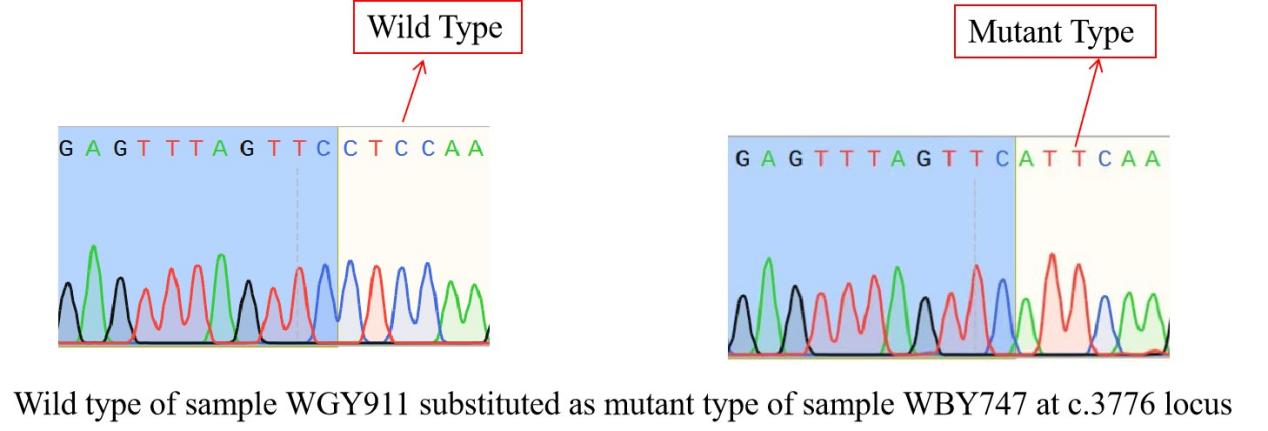


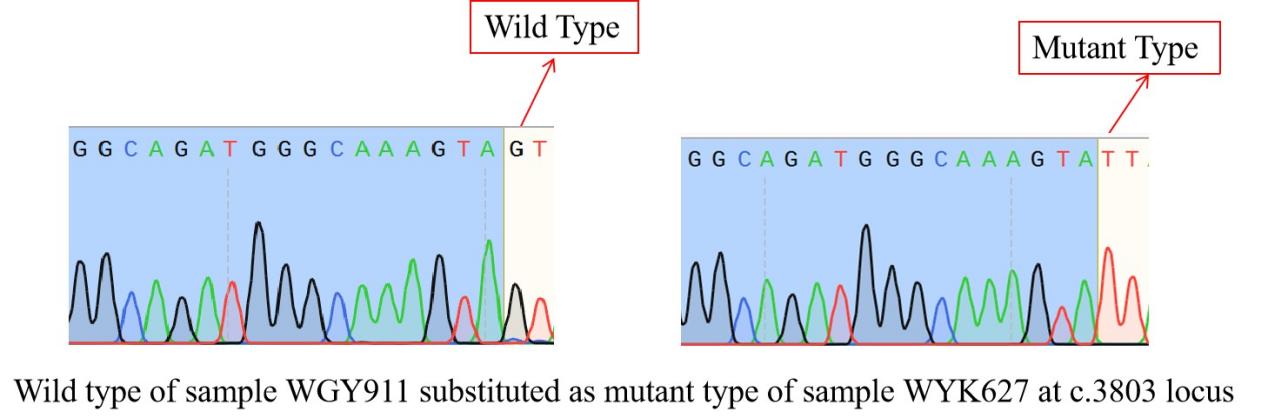


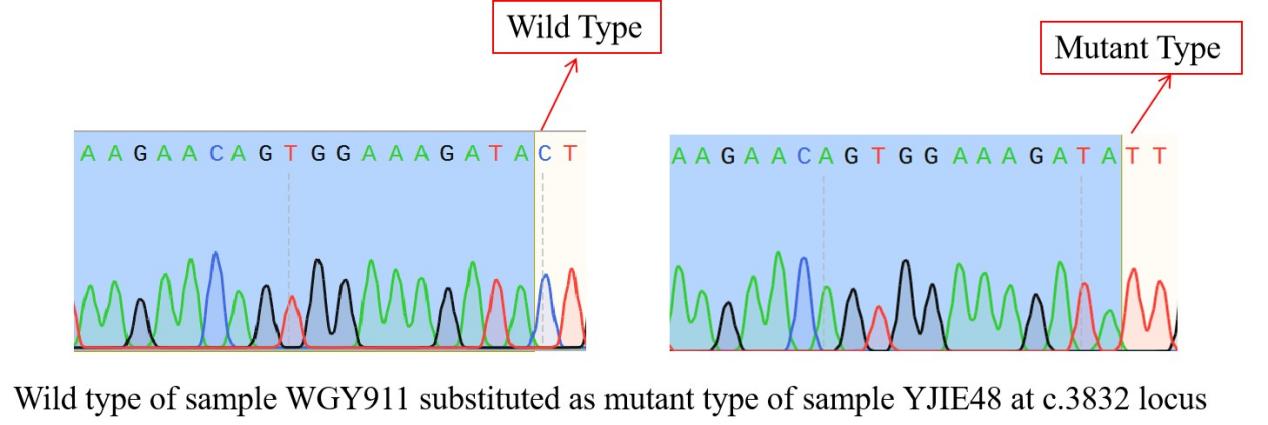


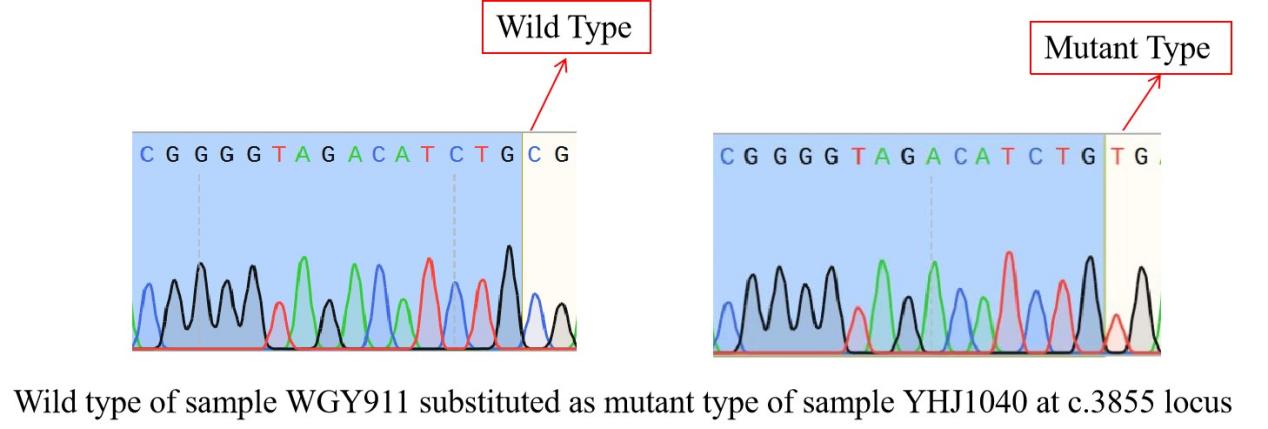

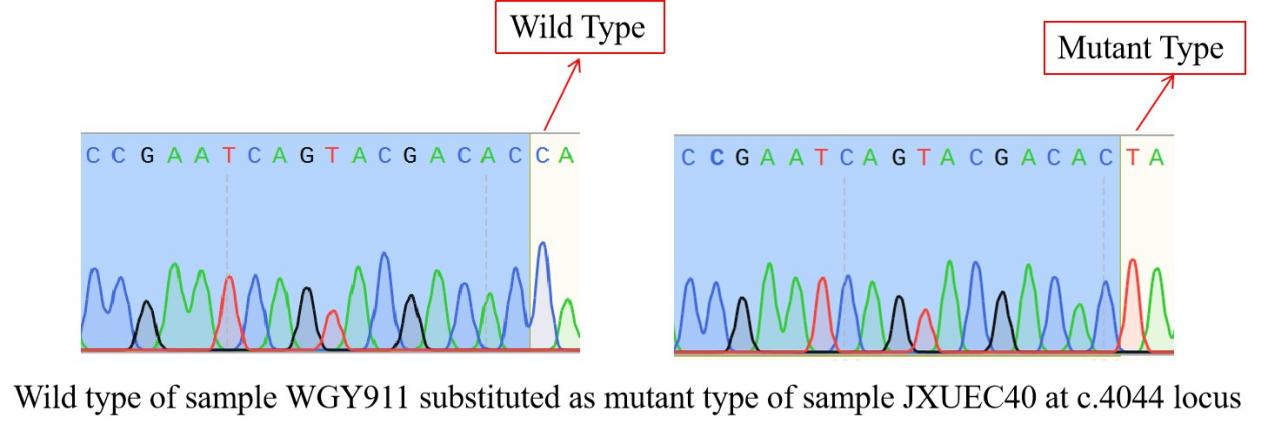


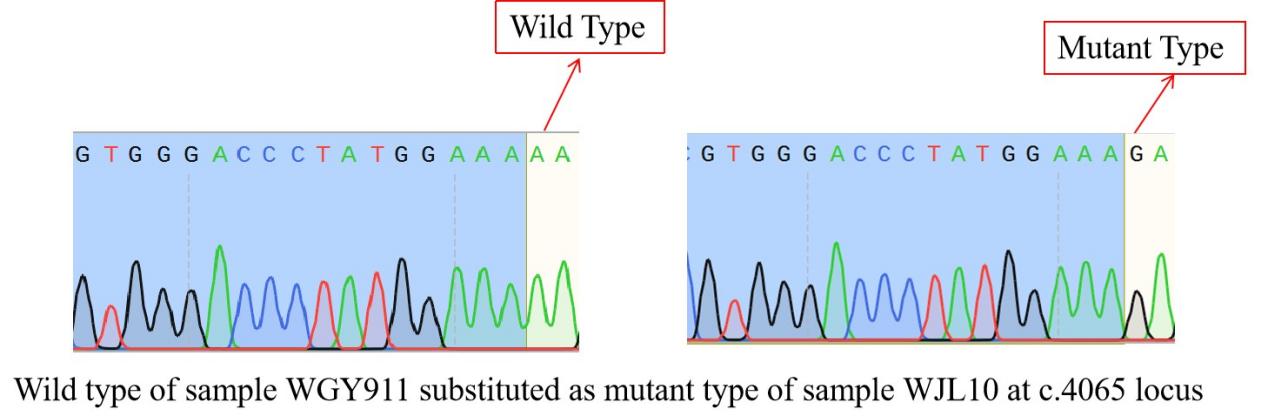


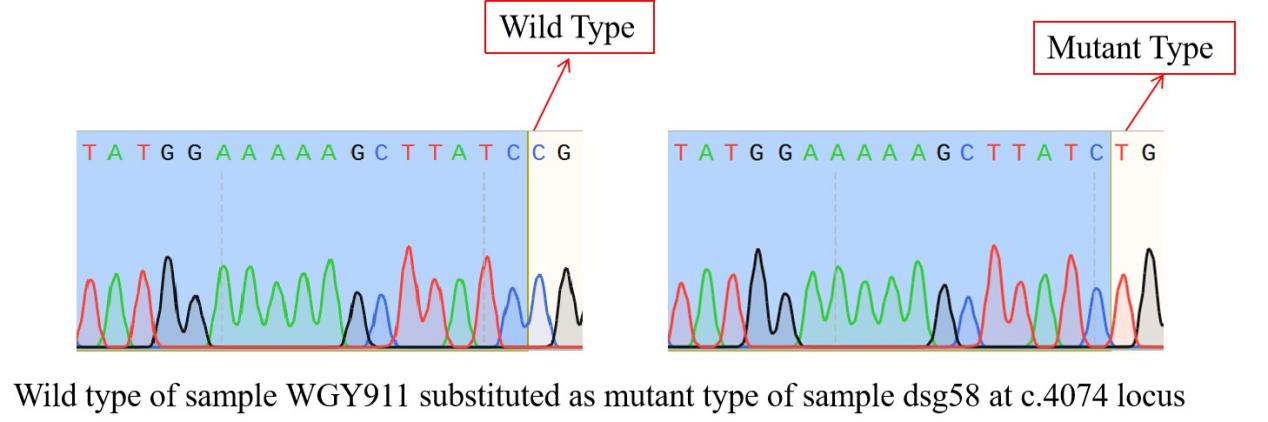


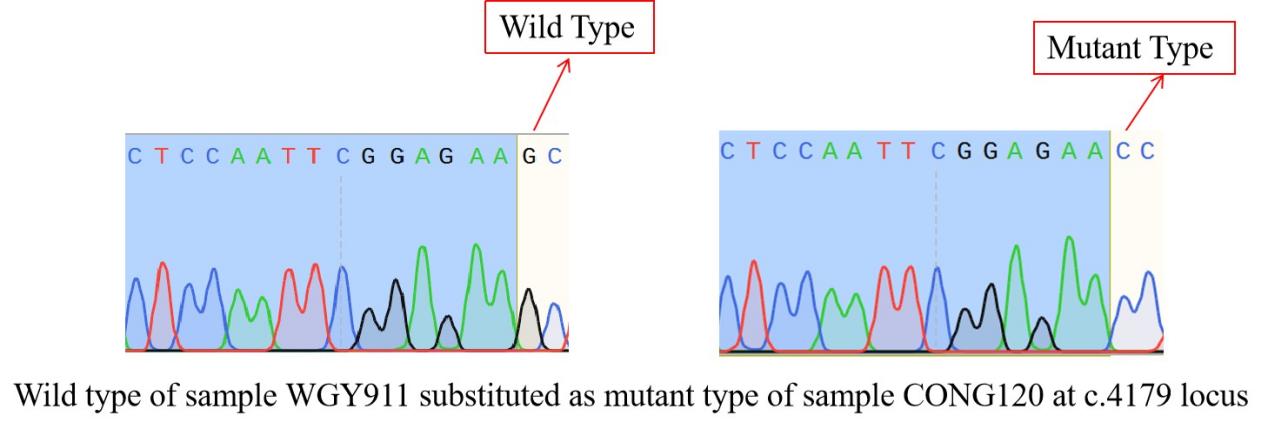

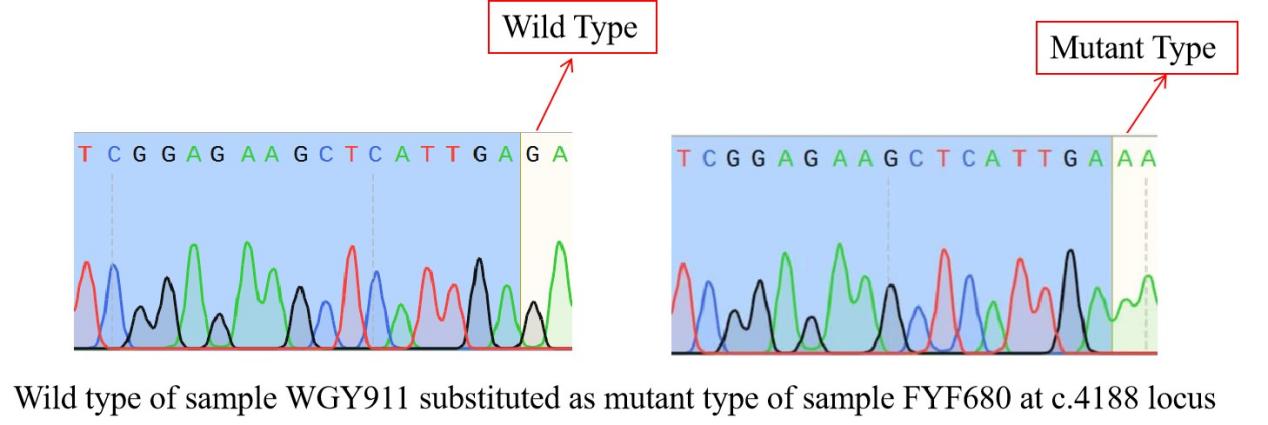


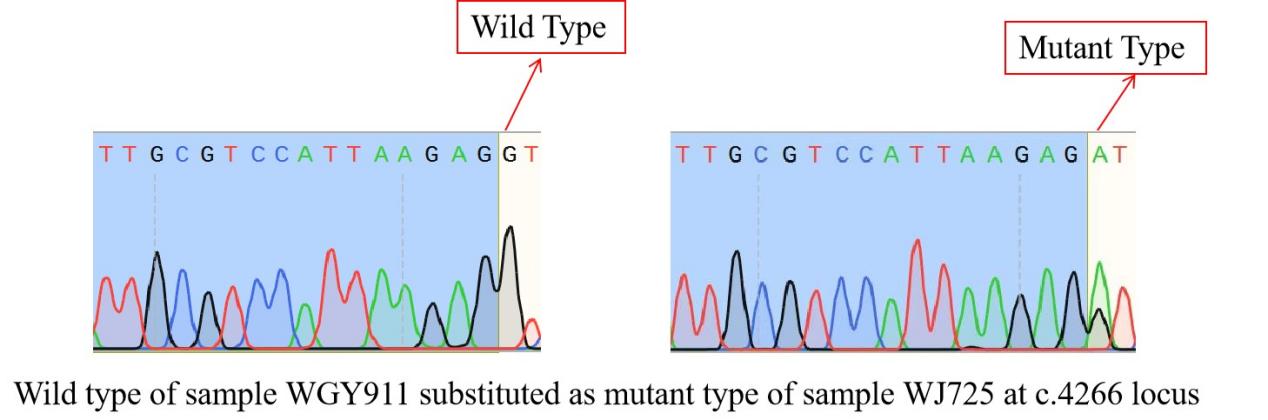


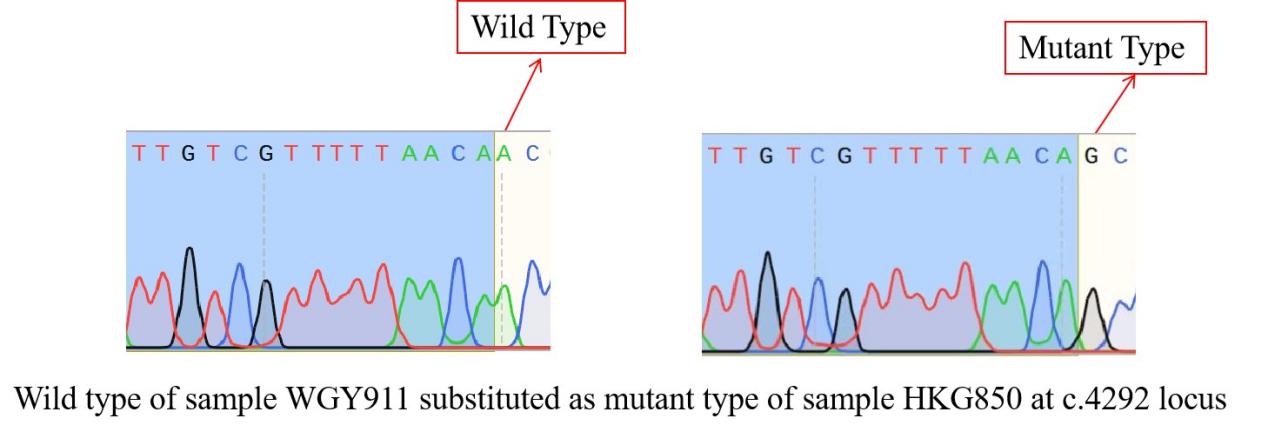


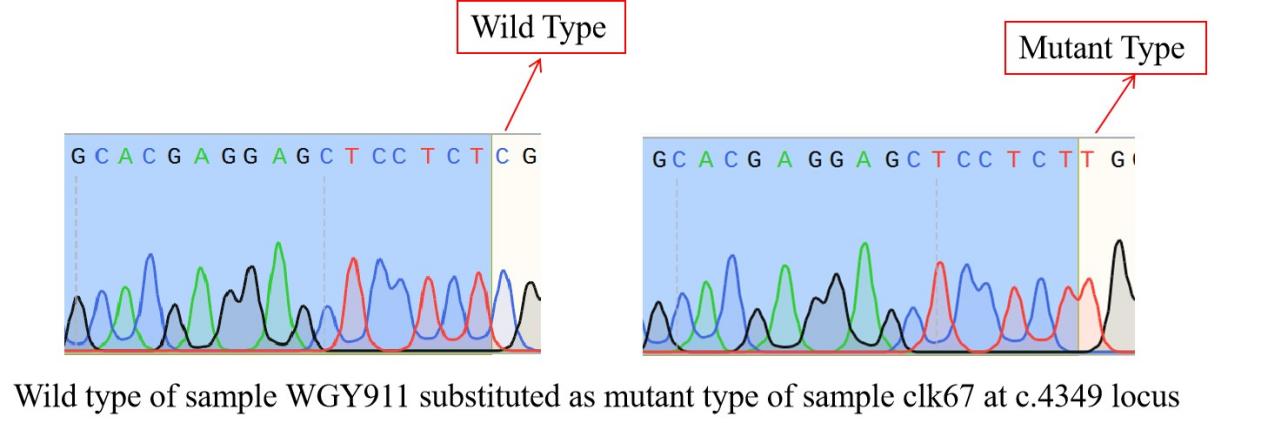

Supplement: Supplementary file 5 — Additional file 5: Identify true base substitutions. [file 12936_2023_4616_MOESM5_ESM.docx]
