# Supplementary material for: Characteristics of molecular markers associated with chloroquine resistance in Plasmodium vivax strains from vivax malaria cases in Yunnan Province, China
Source: Malar J. 2023 Jun 11;22:181. doi: 10.1186/s12936-023-04616-0 (PMC10257827; doi:10.1186/s12936-023-04616-0)
Supplement: Supplementary file 7 — Additional file 7. The composition of 105 haplotypes identified from 624 pvmdr1 gene sequences. [file 12936_2023_4616_MOESM7_ESM.docx]

**Additional file 7**

**The composition of 105 haplotypes identified from 624 *pvmdr1* gene sequences**

| **Table1 The composition of multiple mutation loci in 105 haplotypes identified from 624 *pvmdr1* CDSs in *P. vivax* strains** | | | | | | | | |
| --- | --- | --- | --- | --- | --- | --- | --- | --- |
|  |  | **Multiple mutants of different degrees** | | **Frequency** | | | | |
| **Order** | **Haplotype** | **Types** | **Multiplicity** | **No. of all CDSs**  **（n=624）** | **2014（n=283）** | **2020（n=140）** | **2021（n=119）** | **2022（n=82）** |
| 1 | Hap_87 | G698S/M908L/T958M | 3 | 1(0.2%) | 1(0.4%) | 0 | 0 | 0 |
| 2 | Hap_8 | G698S/M908L/T958M/S1358S | 4 | 17(2.7%) | 17(6.0%) | 0 | 0 | 0 |
| 3 | Hap_12 | T529T/G698S/M908L/T958M | 4 | 5(0.8%) | 5(1.8%) | 0 | 0 | 0 |
| 4 | Hap_56 | K44K/G698S/M908L/T958M | 4 | 3(0.5%) | 3(1.1%) | 0 | 0 | 0 |
| 5 | Hap_64 | G698S/M908L/T958M/K1393N | 4 | 1(0.2%) | 1(0.4%) | 0 | 0 | 0 |
| 6 | Hap_90 | G698S/M908L/T958M/F1076L | 4 | 1(0.2%) | 0 | 1(0.7%) | 0 | 0 |
| 7 | Hap_7 | G698S/M908L/T958M/Y976F/F1076L | 5 | 1(0.2%) | 1(0.4%) | 0 | 0 | 0 |
| 8 | Hap_17 | S513R/T529T/G698S/M908L/T958M | 5 | 4(0.6%) | 4(1.4%) | 0 | 0 | 0 |
| 9 | Hap_21 | T529T/G698S/M908L/T958M/K1393N | 5 | 8(1.3%) | 8(2.8%) | 0 | 0 | 0 |
| 10 | Hap_26 | S513R/T529T /A861E /M908L /T958M | 5 | 2(0.3%) | 2(0.7%) | 0 | 0 | 0 |
| 11 | Hap_28 | G698S/ L845F /M908L /T958M/ F1076L | 5 | 9(1.4%) | 6(2.1%) | 2(1.4%) | 1(0.8%) | 0 |
| 12 | Hap_30 | K44K /G698S/ M908L/ T958M /F1076L | 5 | 3(0.5%) | 2(0.7%) | 1(0.7%) | 0 | 0 |
| 13 | Hap_32 | S513R/ T529T /M908L/ T958M/ F1076L | 5 | 4(0.6%) | 4(1.4%) | 0 | 0 | 0 |
| 14 | Hap_33 | S513R/ T529T /M908L/ T958M /K1393N | 5 | 7(1.1%) | 7(2.5%) | 0 | 0 | 0 |
| 15 | Hap_35 | L493L/T529T /G698S/ M908L/ T958M | 5 | 1(0.2%) | 1(0.3%) | 0 | 0 | 0 |
| 16 | Hap_40 | T529T/ G698S /M908L/ T958M /F1076L | 5 | 66(10.6%) | 8(2.8%) | 29(20.7%) | 19(16.0%) | 10(12.2%) |
| 17 | Hap_57 | T529T/ G698S /M908L/T958M/S1358S | 5 | 6(1.0%) | 2(0.7%) | 0 | 0 | 4(4.9%) |
| 18 | Hap_93 | K44K/T529T/M908L/T958M/F1076L | 5 | 1(0.2%) | 0 | 1(0.7%) | 0 | 0 |
| 19 | Hap_95 | T529T/M908L/T958M/F1076L/K1393N | 5 | 9(1.4%) | 0 | 0 | 4(3.4%) | 5(6.1%) |
| 20 | Hap_2 | S513R/T529T/G698S/M908L/T958M/S1358S | 6 | 32(5.1%) | 3(1.1%) | 21(15.0%) | 4(3.4%) | 4(4.9%) |
| 21 | Hap_10 | T248T/T529T/G698S/M908L/T958M/F1076L | 6 | 1(0.2%) | 1(0.4%) | 0 | 0 | 0 |
| 22 | Hap_13 | L310L/S513R/T529T/G698S/M908L/T958M | 6 | 2(0.3%) | 2(0.7%) | 0 | 0 | 0 |
| 23 | Hap_18 | K44K/T529T/G698S/M908L/T958M/F1076L | 6 | 11(1.8%) | 9(3.2%) | 0 | 1(0.8%) | 1(1.2%) |
| 24 | Hap_20 | L493L/T529T/G698S/M908L/T958M/F1076L | 6 | 2(0.3%) | 2(0.7%) | 0 | 0 | 0 |
| 25 | Hap_23 | S513R/T529T/G698S/M908L/T958M/K1393N | 6 | 35(5.6%) | 24(8.5%) | 6(4.3%) | 4(3.4%) | 1(1.2%) |
| 26 | Hap_29 | T529T/G698S/L845F/M908L/T958M /F1076L | 6 | 9(1.4%) | 9(3.2%) | 0 | 0 | 0 |
| 27 | Hap_31 | T529T/G698S/A861E/M908L/T958M/K1393N | 6 | 4(0.6%) | 4(1.4%) | 0 | 0 | 0 |
| 28 | Hap_34 | G698S/M908L/T958M/Y976F/F1076L/K1393N | 6 | 1(0.2%) | 1(0.4%) | 0 | 0 | 0 |
| 29 | Hap_36 | K44K/S513R/G698S/ M908L /T958M /K1393N | 6 | 1(0.2%) | 1(0.4%) | 0 | 0 | 0 |
| 30 | Hap_37 | S513R/T529T/A861E/M908L/ T958M /K1393N | 6 | 1(0.2%) | 1(0.4%) | 0 | 0 | 0 |
| 31 | Hap_46 | K44K/G698S/M908L/T958M /F1076L /E1396E | 6 | 1(0.2%) | 1(0.4%) | 0 | 0 | 0 |
| 32 | Hap_47 | S513R/T529T /G698S/ A861E /M908L/ T958M | 6 | 1(0.2%) | 1(0.4%) | 0 | 0 | 0 |
| 33 | Hap_51 | T529T/G698S/M908L/T958M/F1076L/K1393N | 6 | 2(0.3%) | 1(0.4%) | 0 | 1(0.8%) | 0 |
| 34 | Hap_53 | L493L/T529T/G698S/M908L/T958M/S1358S | 6 | 4(0.6%) | 4(1.4%) | 0 | 0 | 0 |
| 35 | Hap_55 | G172G/S513R/T529T/M908L/T958M/F1076L | 6 | 1(0.2%) | 1(0.4%) | 0 | 0 | 0 |
| 36 | Hap_62 | K44K/G698S/L845F/M908L/T958M/F1076L | 6 | 1(0.2%) | 1(0.4%) | 0 | 0 | 0 |
| 37 | Hap_71 | S513R/T529T/G698S/M908L/T958M/L1067L | 6 | 2(0.3%) | 2(0.7%) | 0 | 0 | 0 |
| 38 | Hap_73 | L493L/G698S/M908L/T958M/F1076L/E1396E | 6 | 3(0.5%) | 3(1.1%) | 0 | 0 | 0 |
| 39 | Hap_74 | T409M/T529T/G698S/M908L/T958M/S1358S | 6 | 1(0.2%) | 1(0.4%) | 0 | 0 | 0 |
| 40 | Hap_76 | S513R/T529T/K672N/M908L/T958M/F1076L | 6 | 1(0.2%) | 1(0.4%) | 0 | 0 | 0 |
| 41 | Hap_77 | S513R/T529T/G698S/M908L/T958M/K997R | 6 | 3(0.5%) | 3(1.1%) | 0 | 0 | 0 |
| 42 | Hap_79 | T529T/G698S/M908L/T958M/F1076L/E1396E | 6 | 1(0.2%) | 1(0.4%) | 0 | 0 | 0 |
| 43 | Hap_85 | K44K/G698S/M833I/M908L/T958M/F1076L | 6 | 1(0.2%) | 1(0.4%) | 0 | 0 | 0 |
| 44 | Hap_89 | K44K/G698S/M908L/T958M/L1212L/N1431S | 6 | 1(0.2%) | 1(0.4%) | 0 | 0 | 0 |
| 45 | Hap_91 | P8L/L310L/T529T/M908L/T958M/F1076L | 6 | 58(9.3%) | 0 | 19(13.6%) | 24(20.2%) | 15(18.3%) |
| 46 | Hap_92 | T529T/G698S/A861E/M908L/T958M/F1076L | 6 | 1(0.2%) | 0 | 1(0.7%) | 0 | 0 |
| 47 | Hap_96 | S513R/T529T/G698S/M908L/T958M/F1076L | 6 | 2(0.3%) | 0 | 0 | 1(0.8%) | 1(1.2%) |
| 48 | Hap_97 | S513R/T529T/M908L/T958M/F1076L/S1358S | 6 | 2(0.3%) | 0 | 0 | 1(0.8%) | 1(1.2%) |
| 49 | Hap_99 | T529T/G698S/M908L/T958M/F1076L/S1358S | 6 | 1(0.2%) | 0 | 0 | 1(0.8%) | 0 |
| 50 | Hap_101 | T409M/T529T/M908L/T958M/F1076L/K1393N | 6 | 1(0.2%) | 0 | 0 | 1(0.8%) | 0 |
| 51 | Hap_104 | K44K/L310L/T529T/M908L/T958M/F1076L | 6 | 1(0.2%) | 0 | 0 | 0 | 1(1.2%) |
| 52 | Hap_4 | K44K/T529T/G698S/L845F/M908L/T958M/F1076L | 7 | 47(7.5%) | 2(0.7%) | 10(7.1%) | 14(11.8%) | 21(25.6%) |
| 53 | Hap_6 | S513R/T529T/G698S/M908L/T958M/Y976F/F1076L | 7 | 3(0.5%) | 3(1.1%) | 0 | 0 | 0 |
| 54 | Hap_9 | T409M/T529T/G698S/M908L/T958M/F1076L/K1393N | 7 | 29(4.7%) | 11(3.9%) | 8(5.7%) | 4(3.4%) | 6(7.3%) |
| 55 | Hap_11 | K44K/T529T/G698S/A861E/M908L/T958M/F1076L | 7 | 45(7.2%) | 12(4.2%) | 15(10.7%) | 17(14.3%) | 1(1.2%) |
| 56 | Hap_19 | L493L/G698S/L845F/M908L/T958M/A1049A/F1076L | 7 | 1(0.2%) | 1(0.4%) | 0 | 0 | 0 |
| 57 | Hap_24 | K44K/T529T/G698S/M908L/T958M/ F1076L/ S1450L | 7 | 52(8.3%) | 17(6.0%) | 16(11.4%) | 13(10.9%) | 6(7.3%) |
| 58 | Hap_25 | S513R/T529T/G698S/M908L/T958M /S1358S /S1450L | 7 | 1(0.2%) | 1(0.4%) | 0 | 0 | 0 |
| 59 | Hap_27 | L493L/T529T/ G698S /L845F /M908L /T958M/ S1358S | 7 | 1(0.2%) | 1(0.4%) | 0 | 0 | 0 |
| 60 | Hap_39 | L493L/T529T/G698S/L845F /M908L /T958M /F1076L | 7 | 4(0.6%) | 4(1.4%) | 0 | 0 | 0 |
| 61 | Hap_43 | K44K/G520D/T529T /G698S/M908L /T958M/ F1076L | 7 | 5(0.8%) | 5(1.8%) | 0 | 0 | 0 |
| 62 | Hap_44 | T529T/G698S/L845F/M908L/T958M/F1076L/R1422R | 7 | 1(0.2%) | 1(0.4%) | 0 | 0 | 0 |
| 63 | Hap_48 | K44K/S513R/T529T/G698S/M908L/T958M/K1393N | 7 | 1(0.2%) | 1(0.4%) | 0 | 0 | 0 |
| 64 | Hap_49 | G172G/T529T/G698S/M908L/T958M/S1258S/ S1259F | 7 | 2(0.3%) | 2(0.7%) | 0 | 0 | 0 |
| 65 | Hap_52 | K44K/L493L/T529T/G698S/M908L/T958M/F1076L | 7 | 1(0.2%) | 1(0.4%) | 0 | 0 | 0 |
| 66 | Hap_54 | K44K/N312N/T529T/G698S/M908L/T958M/F1076L | 7 | 1(0.2%) | 1(0.4%) | 0 | 0 | 0 |
| 67 | Hap_59 | S513R/T529T/G698S/M908L/T958M/F1076L/K1393N | 7 | 2(0.3%) | 2(0.7%) | 0 | 0 | 0 |
| 68 | Hap_61 | N312N/L493L/T529T/G698S/M908L/T958M/K997R | 7 | 1(0.2%) | 1(0.4%) | 0 | 0 | 0 |
| 69 | Hap_63 | T529T/V651V/G698S/L845F/M908L/T958M/F1076L | 7 | 1(0.2%) | 1(0.4%) | 0 | 0 | 0 |
| 70 | Hap_65 | K44K/G698S/M908L/T958M/F1076L/L1212L/N1431S | 7 | 6(1.0%) | 6(2.1%) | 0 | 0 | 0 |
| 71 | Hap_66 | K44K/E399E/T529T/G698S/M908L/T958M/S1358S | 7 | 1(0.2%) | 1(0.4%) | 0 | 0 | 0 |
| 72 | Hap_68 | T529T/G698S/L845F/M908L/T958M/F1076L/K1393N | 7 | 1(0.2%) | 1(0.4%) | 0 | 0 | 0 |
| 73 | Hap_69 | S513R/T529T/G698S/A861E/M908L/T958M/K1393N | 7 | 2(0.3%) | 2(0.7%) | 0 | 0 | 0 |
| 74 | Hap_72 | Y181Y/T529T/G698S/L845F/M908L/T958M/F1076L | 7 | 1(0.2%) | 1(0.4%) | 0 | 0 | 0 |
| 75 | Hap_75 | K44K/S553S/G698S/L845F/M908L/T958M/F1076L | 7 | 1(0.2%) | 1(0.4%) | 0 | 0 | 0 |
| 76 | Hap_80 | S513R/T529T/G698S/M908L/T958M/F1076L/E1396E | 7 | 1(0.2%) | 1(0.4%) | 0 | 0 | 0 |
| 77 | Hap_81 | L493L/G698S/S786G/M908L/T958M/F1076L/K1393N | 7 | 1(0.2%) | 1(0.4%) | 0 | 0 | 0 |
| 78 | Hap_83 | T529T/G698S/M908L/T958M/F1076L/C1285C/E1396E | 7 | 1(0.2%) | 1(0.4%) | 0 | 0 | 0 |
| 79 | Hap_84 | T529T/G698S/L845F/M908L/T958M/F1076L/S1450L | 7 | 2(0.3%) | 2(0.7%) | 0 | 0 | 0 |
| 80 | Hap_86 | K44K/T529T/G698S/M833I/M908L/T958M/F1076L | 7 | 8(1.3%) | 1(0.4%) | 3(2.1%) | 3(2.5%) | 1(1.2%) |
| 81 | Hap_94 | P8L/L310L/T529T/G698S/M908L/T958M/F1076L | 7 | 1(0.2%) | 0 | 0 | 1(0.8%) | 0 |
| 82 | Hap_98 | K44K/S513R/T529T/G698S/M908L/T958M/S1358S | 7 | 1(0.2%) | 0 | 0 | 1(0.8%) | 0 |
| 83 | Hap_100 | S513R/T529T/M908L/T958M/L1022L/F1076L/K1355K | 7 | 1(0.2%) | 0 | 0 | 1(0.8%) | 0 |
| 84 | Hap_106 | K44K/L310L/T529T/L845F/M908L/T958M/F1076L | 7 | 1(0.2%) | 0 | 0 | 0 | 1(1.2%) |
| 85 | Hap_5 | L493L/T529T/G698S/L845F/M908L/T958M/F1076L/  E1233E | 8 | 3(0.5%) | 3(1.1%) | 0 | 0 | 0 |
| 86 | Hap_22 | K44K/S513R/T529T/G698S/A861E/M908L/T958M/  F1076L | 8 | 1(0.2%) | 1(0.4%) | 0 | 0 | 0 |
| 87 | Hap_41 | S513R/T529T/G698S/A861E/M908L/T958M/F1076L/ K1393N | 8 | 1(0.2%) | 1(0.4%) | 0 | 0 | 0 |
| 88 | Hap_42 | I443I /L493L/ T529T /G698S/ M908L/ T958M/ F1076L/ K1393N | 8 | 1(0.2%) | 1(0.4%) | 0 | 0 | 0 |
| 89 | Hap_45 | S513R/T529T/G698S/A763V/M908L/T958M/F1076L/  S1268I | 8 | 1(0.2%) | 1(0.4%) | 0 | 0 | 0 |
| 90 | Hap_50 | K44K/G520D/T529T/ G698S /M908L /T958M/ F1076L/ S1450L | 8 | 2(0.3%) | 2(0.7%) | 0 | 0 | 0 |
| 91 | Hap_58 | N312N/T529T/G698S/L845F/M908L/T958M/F1070F/  F1076L | 8 | 3(0.5%) | 3(1.1%) | 0 | 0 | 0 |
| 92 | Hap_60 | T529T/G698S/A861E/M908L/T958M/Y976F/F1076L/  K1393N | 8 | 2(0.3%) | 2(0.7%) | 0 | 0 | 0 |
| 93 | Hap_67 | K44K/F185L/T529T/G698S/M908L/T958M/F1076L/  S1450L | 8 | 1(0.2%) | 1(0.4%) | 0 | 0 | 0 |
| 94 | Hap_82 | K44K/T529T/G698S/L845F/M908L/T958M/F1076L/  S1450L | 8 | 1(0.2%) | 1(0.4%) | 0 | 0 | 0 |
| 95 | Hap_103 | S513R/T529T/G698S/M908L/T958M/F1076L/S1358S/  K1393N | 8 | 1(0.2%) | 0 | 0 | 0 | 1(1.2%) |
| 96 | Hap_105 | K44K/T529T/G698S/F842I/L845F/M908L/T958M/  F1076L | 8 | 1(0.2%) | 0 | 0 | 0 | 1(1.2%) |
| 97 | Hap_3 | K44K/T529T/G698S/L845F/M908L/T958M/F1076L/  L1120L/S1450L | 9 | 7(1.1%) | 7(2.5%) | 0 | 0 | 0 |
| 98 | Hap_15 | P8L/K44K/G520D/T529T/G698S/L845F/M908L/  T958M/F1076L | 9 | 2(0.3%) | 2(0.7%) | 0 | 0 | 0 |
| 99 | Hap_16 | P8L/K44K/T529T/G698S/L845F/M908L/T958M/  F1076L/S1450L | 9 | 3(0.5%) | 3(1.1%) | 0 | 0 | 0 |
| 100 | Hap_38 | K44K /S513R /T529T /G698S /L845F/ M908L/ T958M/ F1076L/ S1450L | 9 | 2(0.3%) | 2(0.7%) | 0 | 0 | 0 |
| 101 | Hap_70 | G32C/K44K/L493L/T529T/G698S/L845F/M908L/  T958M/F1076L | 9 | 2(0.3%) | 2(0.7%) | 0 | 0 | 0 |
| 102 | Hap_88 | P437S/L493L/T529T/G698S/L845F/M908L/T958M/  F1076L/E1233E | 9 | 1(0.2%) | 1(0.4%) | 0 | 0 | 0 |
| 103 | Hap_102 | K44K/G698S/L845F/M908L/T958M/F1076L/L1120L/  L1278L/S1450L | 9 | 1(0.2%) | 0 | 0 | 1(0.8%) | 0 |
| 104 | Hap_14 | K44K/G520D/T529T/G698S/A861E/M908L/T958M/  F1076L/T1348T/S1450L | 10 | 23(3.7%) | 13(4.6%) | 7(5.0%) | 2(1.7%) | 1(1.2%) |
| 105 | Hap_78 | K44K/T529T/G698S/A861E/M908L/T958M/F1076L/  T1348T/K1393N/S1450L | 10 | 1(0.2%) | 1(0.4%) | 0 | 0 | 0 |
