# Supplementary material for: Characteristics of molecular markers associated with chloroquine resistance in Plasmodium vivax strains from vivax malaria cases in Yunnan Province, China
Source: Malar J. 2023 Jun 11;22:181. doi: 10.1186/s12936-023-04616-0 (PMC10257827; doi:10.1186/s12936-023-04616-0)
Supplement: Supplementary file 8 — Additional file 8: Fig.S1 The subfigure of haplotypes composition at 2014, Fig. S2 The subfigure of haplotypes composition at 2020, Fig. S3 The subfigure of haplotypes composition at 2021, Fig. S4 The subfigure of haplotypes composition at 2022, respectively. [file 12936_2023_4616_MOESM8_ESM.docx]

**Additional file 8**

**Four subfigures of 105 haplotypes change at 2014, 2020, 2021 and 2022**


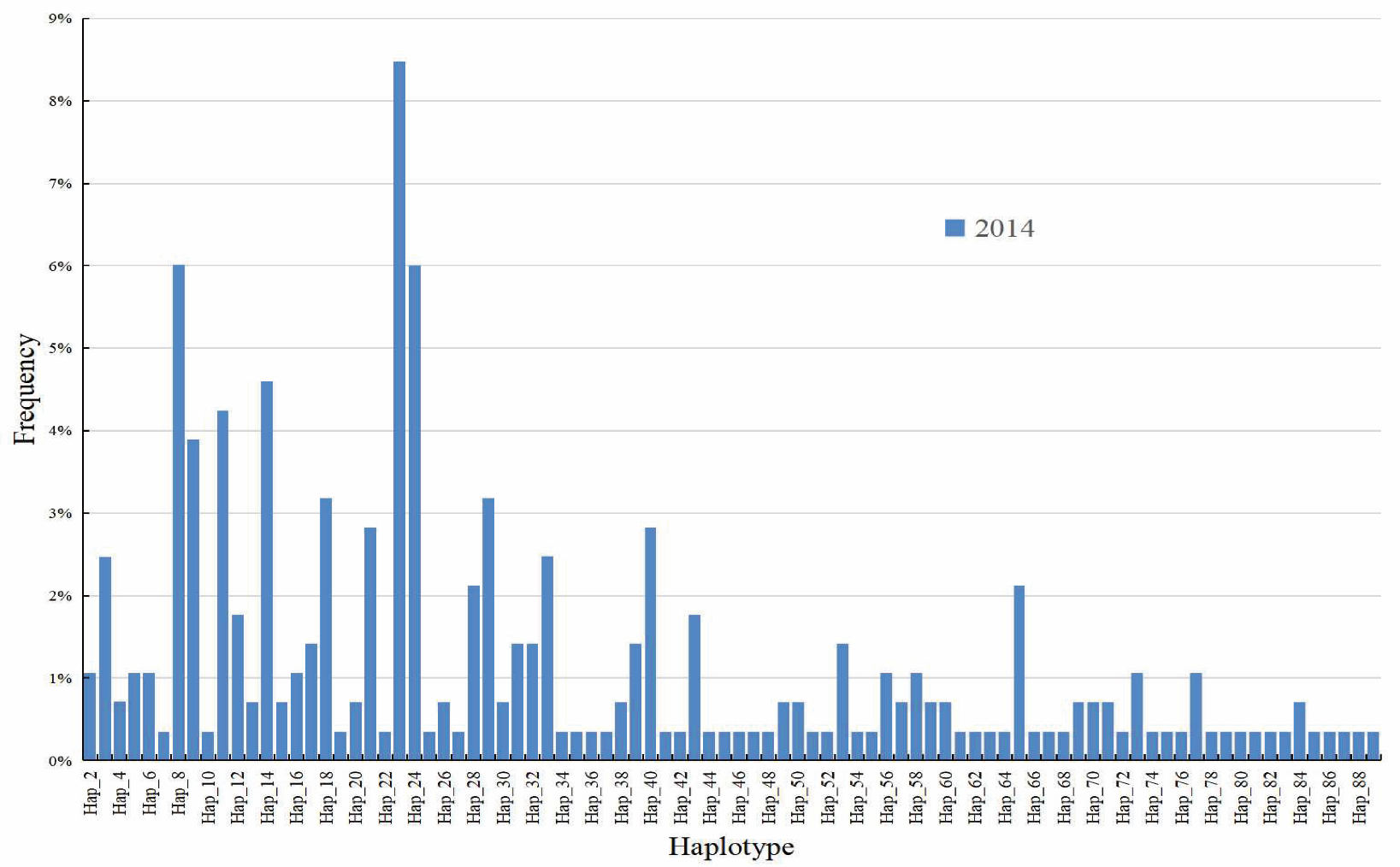


**Fig. S1** **The subfigure of** **haplotypes composition at 2014**


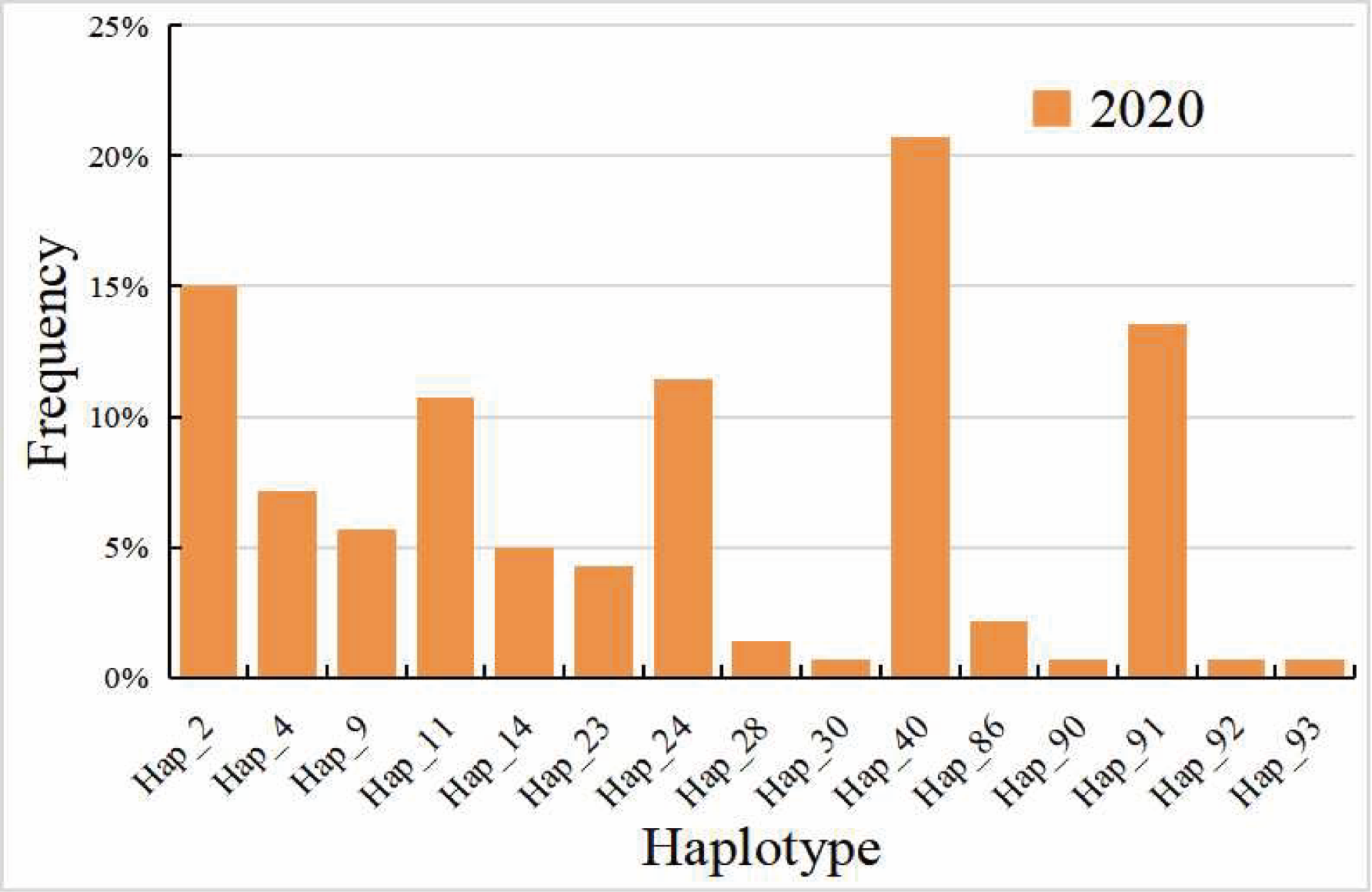


**Fig. S2** **The subfigure of haplotypes composition at 2020**


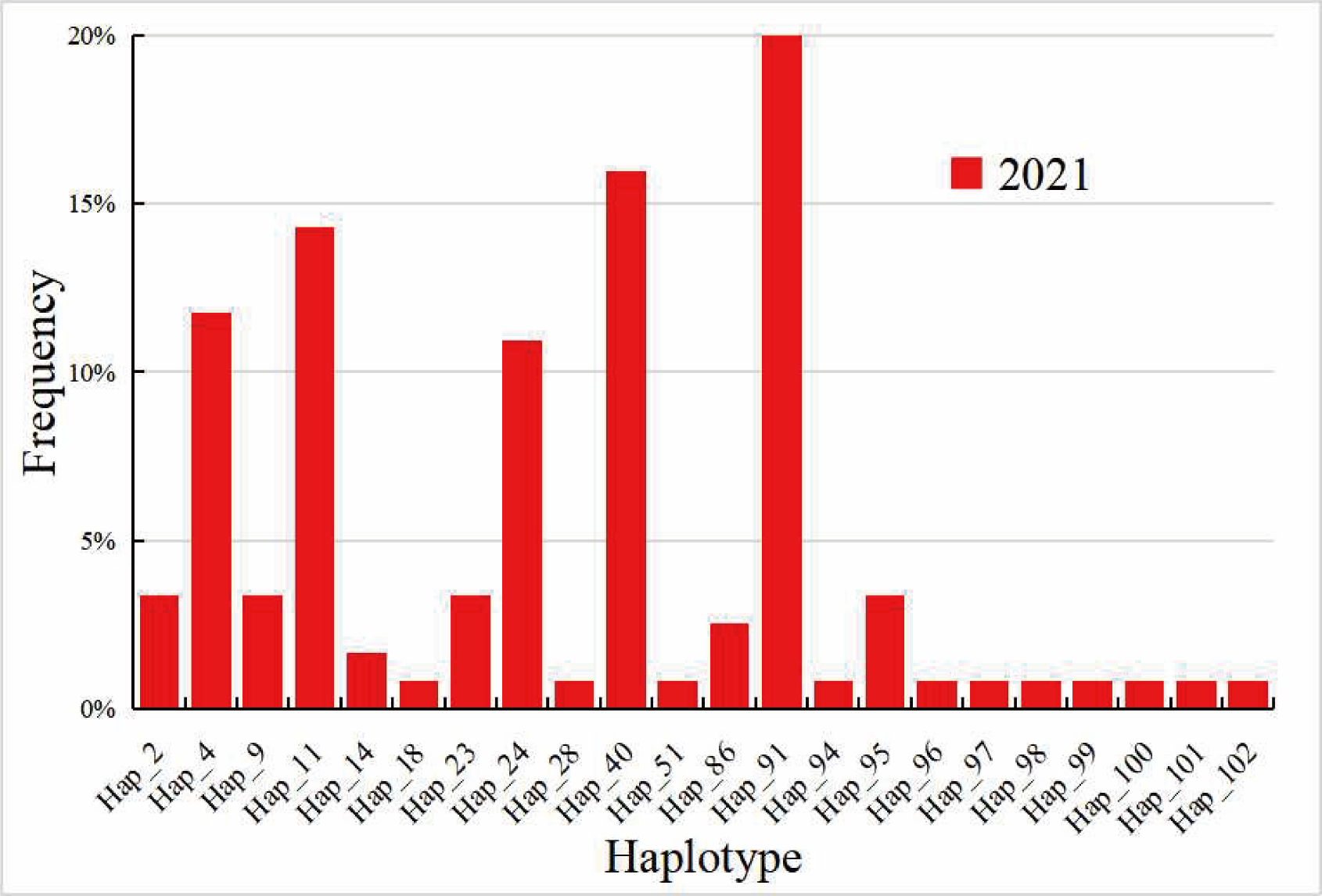


**Fig. S3 The subfigure of haplotypes composition at 2021**


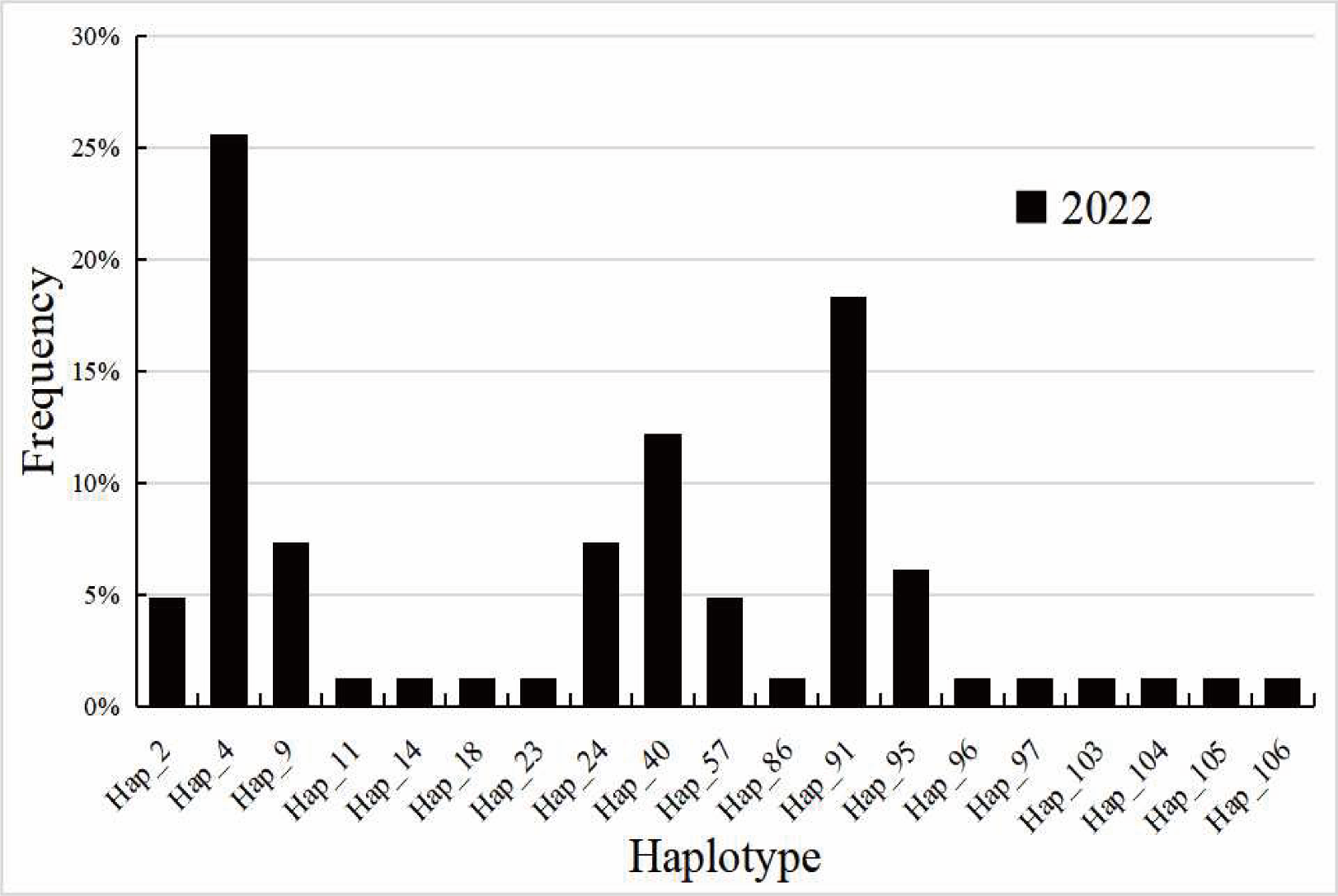


**Fig. S4 The subfigure of haplotypes composition at 2022**
