# Supplementary material for: Characteristics of molecular markers associated with chloroquine resistance in Plasmodium vivax strains from vivax malaria cases in Yunnan Province, China
Source: Malar J. 2023 Jun 11;22:181. doi: 10.1186/s12936-023-04616-0 (PMC10257827; doi:10.1186/s12936-023-04616-0)
Supplement: Supplementary file 9 — Additional file 9. The difference of multiplicity degree in 90 haplotypes identified by between gene sequence alignment and network evolutionary analysis. [file 12936_2023_4616_MOESM9_ESM.docx]

**Additional file 9**

**The difference of multiplicity degree in 90 haplotypes identified by between gene sequence alignment and network evolutionary analysis**

| **Table 1 The difference multiplicities between the network diagram analysis and the sequence alignment on 90 special haplotypes in this study** | | | | | | | | | | | | | | | |
| --- | --- | --- | --- | --- | --- | --- | --- | --- | --- | --- | --- | --- | --- | --- | --- |
| **Order** | **Haplotype** | **Network diagram multiplicity** | **Sequence alignment** **multiplicity** | **Difference multiplicity** | **Number of change sites** | **c.132** | **c.1477** | **c.1539** | **c.1559** | **c.1587** | **c.2092** | **c.3226** | **c.4074** | **c.4179** |  |
| 1 | Hap_6 | 9 | 7 | 2 | 1 | 1 | 1 | 1 | 1 | 1 | 1 | 1 | 1 | 2^*^ |  |
| 2 | Hap_33 | 7 | 5 | 2 |  | 1 | 1 | 1 | 1 | 1 | 2^*^ | 1 | 1 | 1 |  |
| 3 | Hap_36 | 8 | 6 | 2 |  | 1 | 1 | 1 | 1 | 2^*^ | 1 | 1 | 1 | 1 |  |
| 4 | Hap_45 | 10 | 8 | 2 |  | 1 | 1 | 1 | 1 | 1 | 1 | 1 | 1 | 2^*^ |  |
| 5 | Hap_69 | 9 | 7 | 2 |  | 1 | 1 | 1 | 1 | 1 | 1 | 2^*^ | 1 | 1 |  |
| 6 | Hap_96 | 8 | 6 | 2 |  | 1 | 1 | 1 | 1 | 1 | 1 | 1 | 1 | 2^*^ |  |
| 7 | Hap_3 | 13 | 9 | 4 | 2 | 1 | 1 | 2^*^ | 1 | 1 | 1 | 1 | 1 | 2^*^ |  |
| 8 | Hap_4 | 11 | 7 | 4 |  | 1 | 1 | 2^*^ | 1 | 1 | 1 | 1 | 1 | 2^*^ |  |
| 9 | Hap_9 | 11 | 7 | 4 |  | 1 | 1 | 2^*^ | 1 | 1 | 1 | 1 | 1 | 3^#^ |  |
| 10 | Hap_10 | 10 | 6 | 4 |  | 1 | 1 | 2^*^ | 1 | 1 | 1 | 1 | 1 | 2^*^ |  |
| 11 | Hap_11 | 11 | 7 | 4 |  | 1 | 1 | 2^*^ | 1 | 1 | 1 | 1 | 1 | 2^*^ |  |
| 12 | Hap_13 | 10 | 6 | 4 |  | 1 | 1 | 1 | 1 | 1 | 1 | 2^*^ | 1 | 2^*^ |  |
| 13 | Hap_14 | 14 | 10 | 4 |  | 1 | 1 | 2^*^ | 1 | 1 | 1 | 1 | 1 | 2^*^ |  |
| 14 | Hap_15 | 13 | 9 | 4 |  | 1 | 1 | 2^*^ | 1 | 1 | 1 | 1 | 1 | 2^*^ |  |
| 15 | Hap_16 | 13 | 9 | 4 |  | 1 | 1 | 2^*^ | 1 | 1 | 1 | 1 | 1 | 2^*^ |  |
| 16 | Hap_17 | 9 | 5 | 4 |  | 1 | 1 | 1 | 1 | 1 | 1 | 2^*^ | 1 | 2^*^ |  |
| 17 | Hap_18 | 10 | 6 | 4 |  | 1 | 1 | 2^*^ | 1 | 1 | 1 | 1 | 1 | 2^*^ |  |
| 18 | Hap_20 | 10 | 6 | 4 |  | 1 | 1 | 2^*^ | 1 | 1 | 1 | 1 | 1 | 2^*^ |  |
| 19 | Hap_22 | 12 | 8 | 4 |  | 1 | 1 | 2^*^ | 1 | 1 | 1 | 1 | 1 | 2^*^ |  |
| 20 | Hap_24 | 11 | 7 | 4 |  | 1 | 1 | 2^*^ | 1 | 1 | 1 | 1 | 1 | 2^*^ |  |
| 21 | Hap_29 | 10 | 6 | 4 |  | 1 | 1 | 2^*^ | 1 | 1 | 1 | 1 | 1 | 2^*^ |  |
| 22 | Hap_31 | 10 | 6 | 4 |  | 1 | 1 | 2^*^ | 1 | 1 | 1 | 2^*^ | 1 |  |  |
| 23 | Hap_32 | 9 | 5 | 4 |  | 1 | 1 | 1 | 1 | 1 | 2^*^ | 1 | 1 | 2^*^ |  |
| 24 | Hap_37 | 10 | 6 | 4 |  | 1 | 1 | 1 | 1 | 1 | 2^*^ | 2^*^ | 1 |  |  |
| 25 | Hap_38 | 13 | 9 | 4 |  | 1 | 1 | 3^#^ | 1 | 1 | 1 | 1 | 1 | 2^*^ |  |
| 26 | Hap_40 | 9 | 5 | 4 |  | 1 | 1 | 2^*^ | 1 | 1 | 1 | 1 | 1 | 2^*^ |  |
| 27 | Hap_42 | 12 | 8 | 4 |  | 1 | 1 | 2^*^ | 1 | 1 | 1 | 1 | 1 | 3^#^ |  |
| 28 | Hap_43 | 11 | 7 | 4 |  | 1 | 1 | 2^*^ | 1 | 1 | 1 | 1 | 1 | 2^*^ |  |
| 29 | Hap_44 | 11 | 7 | 4 |  | 1 | 1 | 2^*^ | 1 | 1 | 1 | 1 | 1 | 2^*^ |  |
| 30 | Hap_47 | 10 | 6 | 4 |  | 1 | 1 | 1 | 1 | 1 | 1 | 2^*^ | 1 | 2^*^ |  |
| 31 | Hap_50 | 12 | 8 | 4 |  | 1 | 1 | 2^*^ | 1 | 1 | 1 | 1 | 1 | 2^*^ |  |
| 32 | Hap_51 | 10 | 6 | 4 |  | 1 | 1 | 2^*^ | 1 | 1 | 1 | 1 | 1 | 3^#^ |  |
| 33 | Hap_52 | 11 | 7 | 4 |  | 1 | 1 | 2^*^ | 1 | 1 | 1 | 1 | 1 | 2^*^ |  |
| 34 | Hap_54 | 11 | 7 | 4 |  | 1 | 1 | 2^*^ | 1 | 1 | 1 | 1 | 1 | 2^*^ |  |
| 35 | Hap_55 | 10 | 6 | 4 |  | 1 | 1 | 1 | 1 | 1 | 2^*^ | 1 | 1 | 2^*^ |  |
| 36 | Hap_58 | 12 | 8 | 4 |  | 1 | 1 | 2^*^ | 1 | 1 | 1 | 1 | 1 | 2^*^ |  |
| 37 | Hap_60 | 12 | 8 | 4 |  | 1 | 1 | 2^*^ | 1 | 1 | 1 | 1 | 1 | 3^#^ |  |
| 38 | Hap_63 | 11 | 7 | 4 |  | 1 | 1 | 2^*^ | 1 | 1 | 1 | 1 | 1 | 2^*^ |  |
| 39 | Hap_67 | 12 | 8 | 4 |  | 1 | 1 | 2^*^ | 1 | 1 | 1 | 1 | 1 | 2^*^ |  |
| 40 | Hap_68 | 11 | 7 | 4 |  | 1 | 1 | 2^*^ | 1 | 1 | 1 | 1 | 1 | 2^*^ |  |
| 41 | Hap_70 | 13 | 9 | 4 |  | 1 | 1 | 2^*^ | 1 | 1 | 1 | 1 | 1 | 2^*^ |  |
| 42 | Hap_71 | 10 | 6 | 4 |  | 1 | 1 | 1 | 1 | 1 | 1 | 2^*^ | 1 | 2^*^ |  |
| 43 | Hap_72 | 11 | 7 | 4 |  | 1 | 1 | 2^*^ | 1 | 1 | 1 | 1 | 1 | 2^*^ |  |
| 44 | Hap_76 | 10 | 6 | 4 |  | 1 | 1 | 1 | 1 | 1 | 2^*^ | 1 | 1 | 2^*^ |  |
| 45 | Hap_77 | 10 | 6 | 4 |  | 1 | 1 | 1 | 1 | 1 | 1 | 2^*^ | 1 | 2^*^ |  |
| 46 | Hap_79 | 10 | 6 | 4 |  | 1 | 1 | 2^*^ | 1 | 1 | 1 | 1 | 1 | 2^*^ |  |
| 47 | Hap_80 | 11 | 7 | 4 |  | 1 | 1 | 2^*^ | 1 | 1 | 1 | 1 | 1 | 2^*^ |  |
| 48 | Hap_82 | 12 | 8 | 4 |  | 1 | 1 | 2^*^ | 1 | 1 | 1 | 1 | 1 | 2^*^ |  |
| 49 | Hap_83 | 11 | 7 | 4 |  | 1 | 1 | 2^*^ | 1 | 1 | 1 | 1 | 1 | 2^*^ |  |
| 50 | Hap_86 | 11 | 7 | 4 |  | 1 | 1 | 2^*^ | 1 | 1 | 1 | 1 | 1 | 2^*^ |  |
| 51 | Hap_92 | 10 | 6 | 4 |  | 1 | 1 | 2^*^ | 1 | 1 | 1 | 1 | 1 | 2^*^ |  |
| 52 | Hap_94 | 11 | 7 | 4 |  | 1 | 1 | 2^*^ | 1 | 1 | 1 | 1 | 1 | 2^*^ |  |
| 53 | Hap_97 | 10 | 6 | 4 |  | 1 | 1 | 1 | 1 | 1 | 2^*^ | 1 | 1 | 2^*^ |  |
| 54 | Hap_99 | 10 | 6 | 4 |  | 1 | 1 | 2^*^ | 1 | 1 | 1 | 1 | 1 | 2^*^ |  |
| 55 | Hap_100 | 11 | 7 | 4 |  | 1 | 1 | 1 | 1 | 1 | 2^*^ | 1 | 1 | 2^*^ |  |
| 56 | Hap_105 | 12 | 8 | 4 |  | 1 | 1 | 2^*^ | 1 | 1 | 1 | 1 | 1 | 2^*^ |  |
| 57 | Hap_2 | 12 | 6 | 6 | 3 | 1 | 1 | 2^*^ | 1 | 1 | 1 | 2^*^ | 1 | 2^*^ |  |
| 58 | Hap_5 | 14 | 8 | 6 |  | 2^*^ | 1 | 2^*^ | 1 | 1 | 1 | 1 | 1 | 2^*^ |  |
| 59 | Hap_7 | 13 | 5 | 8 |  | 1 | 1 | 2^*^ | 1 | 2^*^ | 1 | 1 | 1 | 4^*^ |  |
| 60 | Hap_12 | 10 | 4 | 6 |  | 1 | 1 | 2^*^ | 1 | 1 | 1 | 2^*^ | 1 | 2^*^ |  |
| 61 | Hap_25 | 13 | 7 | 6 |  | 1 | 1 | 2^*^ | 1 | 1 | 1 | 2^*^ | 1 | 2^*^ |  |
| 62 | Hap_26 | 11 | 5 | 6 |  | 1 | 1 | 1 | 1 | 1 | 2^*^ | 2^*^ | 1 | 2^*^ |  |
| 63 | Hap_27 | 13 | 7 | 6 |  | 1 | 1 | 2^*^ | 1 | 1 | 1 | 2^*^ | 1 | 2^*^ |  |
| 64 | Hap_34 | 12 | 6 | 6 |  | 1 | 1 | 2^*^ | 1 | 2^*^ | 1 | 1 | 1 | 3^#^ |  |
| 65 | Hap_39 | 13 | 7 | 6 |  | 2^*^ | 1 | 2^*^ | 1 | 1 | 1 | 1 | 1 | 2^*^ |  |
| 66 | Hap_49 | 13 | 7 | 6 |  | 1 | 1 | 2^*^ | 1 | 1 | 1 | 2^*^ | 1 | 2^*^ |  |
| 67 | Hap_53 | 12 | 6 | 6 |  | 1 | 1 | 2^*^ | 1 | 1 | 1 | 2^*^ | 1 | 2^*^ |  |
| 68 | Hap_57 | 11 | 5 | 6 |  | 1 | 1 | 2^*^ | 1 | 1 | 1 | 2^*^ | 1 | 2^*^ |  |
| 69 | Hap_66 | 13 | 7 | 6 |  | 1 | 1 | 2^*^ | 1 | 1 | 1 | 2^*^ | 1 | 2^*^ |  |
| 70 | Hap_73 | 12 | 6 | 6 |  | 1 | 1 | 2^*^ | 1 | 2^*^ | 1 | 1 | 1 | 2^*^ |  |
| 71 | Hap_74 | 12 | 6 | 6 |  | 1 | 1 | 2^*^ | 1 | 1 | 1 | 2^*^ | 1 | 2^*^ |  |
| 72 | Hap_78 | 16 | 10 | 6 |  | 1 | 1 | 2^*^ | 2^*^ | 1 | 1 | 1 | 1 | 3^#^ |  |
| 73 | Hap_81 | 11 | 4 | 6 |  | 1 | 1 | 2^*^ | 1 | 2^*^ | 1 | 1 | 1 | 3^#^ |  |
| 74 | Hap_84 | 13 | 7 | 6 |  | 2^*^ | 1 | 2^*^ | 1 | 1 | 1 | 1 | 1 | 2^*^ |  |
| 75 | Hap_85 | 12 | 6 | 6 |  | 1 | 1 | 2^*^ | 1 | 2^*^ | 1 | 1 | 1 | 2^*^ |  |
| 76 | Hap_88 | 15 | 9 | 6 |  | 2^*^ | 1 | 2^*^ | 1 | 1 | 1 | 1 | 1 | 2^*^ |  |
| 77 | Hap_90 | 10 | 4 | 6 |  | 1 | 1 | 2^*^ | 1 | 2^*^ | 1 | 1 | 1 | 2^*^ |  |
| 78 | Hap_91 | 12 | 6 | 6 |  | 1 | 1 | 2^*^ | 1 | 1 | 2^*^ | 1 | 1 | 2^*^ |  |
| 79 | Hap_93 | 11 | 5 | 6 |  | 1 | 1 | 2^*^ | 1 | 1 | 2^*^ | 1 | 1 | 2^*^ |  |
| 80 | Hap_95 | 11 | 5 | 6 |  | 1 | 1 | 2^*^ | 1 | 1 | 2^*^ | 1 | 1 | 3^#^ |  |
| 81 | Hap_98 | 13 | 7 | 6 |  | 1 | 1 | 2^*^ | 1 | 1 | 1 | 2^*^ | 1 | 2^*^ |  |
| 82 | Hap_101 | 12 | 6 | 6 |  | 1 | 1 | 2^*^ | 1 | 1 | 2^*^ | 1 | 1 | 3^#^ |  |
| 83 | Hap_102 | 15 | 9 | 6 |  | 1 | 1 | 2^*^ | 1 | 2^*^ | 1 | 1 | 1 | 2^*^ |  |
| 84 | Hap_104 | 12 | 6 | 6 |  | 1 | 1 | 2^*^ | 1 | 1 | 2^*^ | 1 | 1 | 2^*^ |  |
| 85 | Hap_106 | 13 | 7 | 6 |  | 1 | 1 | 2^*^ | 1 | 1 | 2^*^ | 1 | 1 | 2^*^ |  |
| 86 | Hap_8 | 12 | 4 | 8 | 4 | 1 | 1 | 2^*^ | 1 | 2^*^ | 1 | 2^*^ | 1 | 2^*^ |  |
| 87 | Hap_19 | 15 | 7 | 8 |  | 2^*^ | 1 | 2^*^ | 1 | 2^*^ | 1 | 1 | 1 | 2^*^ |  |
| 88 | Hap_35 | 13 | 5 | 8 |  | 1 | 1 | 2^*^ | 1 | 1 | 1 | 2^*^ | 2^*^ | 2^*^ |  |
| 89 | Hap_61 | 15 | 7 | 8 |  | 1 | 1 | 2^*^ | 1 | 1 | 1 | 2^*^ | 2^*^ | 2^*^ |  |
| 90 | Hap_28 | 15 | 5 | 10 | 5 | 2^*^ | 2^*^ | 2^*^ | 1 | 2^*^ | 1 | 1 | 1 | 2^*^ |  |
| Note: *: Multiple reversals of loci mutations to wild type; #: Loci mutation is still mutant for many times | | | | | | | | | | | | | | |  |
