# Supplementary material for: Characteristics of molecular markers associated with chloroquine resistance in Plasmodium vivax strains from vivax malaria cases in Yunnan Province, China
Source: Malar J. 2023 Jun 11;22:181. doi: 10.1186/s12936-023-04616-0 (PMC10257827; doi:10.1186/s12936-023-04616-0)
Supplement: Supplementary file 10 — Additional file 10. The predicted 3D structural diagram of P. vivax multidrug resistance protein 1 (PvMDR1). [file 12936_2023_4616_MOESM10_ESM.docx]

**Additional file 10**

**The predicted 3D structural diagram of *P. vivax* multidrug resistance protein 1 (*Pv*MDR1)**

The 3D structure of *P**. vivax* multidrug resistance protein 1 (*Pv*MDR1) was predicted using SWISS-MODEL (https://swissmodel.expasy.org/interactive) online software, with 7qks used as a template, and the variant sites were edited using PyMoL 2.3.4 software. *Pv*MDR1 as the digestive vesicle membrane protein of *P. vivax*, accounts for about 2/3 of the amino acid chain immerses in digestive vesicle; all 12 transmembrane domains are α-helical structures spanning 62^th^-84^th^ aa、99^th^-121^th^ aa、171^th^-193^th^ aa、197^th^-216^th^ aa、281^th^-303^th^ aa、323^th^-345^th^ aa、825^th^-847^th^ aa、867^th^-889^th^ aa、940^th^-962^th^ aa、966^th^-985^th^ aa、1062^th^-1084^th^ aa and 1100^th^-1121^th^ aa; the two segments of the β-sheet structure reside outside the digestive vesicle, with both extending about 4 aa in sequence length [46].

The 3D spatial structure of *Pv*MDR1 was shown as the shape of a "V", with the two arms of the "V" and the apical base being inside and outside the digestive vesicle, respectively, and the TMD of *Pv*MDR1 being between them (Fig. 1a). The peptide chain lengths of *Pv*MDR1 in the intra-vesicular, transmembrane, and extra-vesicular membrane surfaces were 53.2% (779 aa/1464 aa), 24.5% (358 aa/1464 aa), and 22.3% (327 aa/1464 aa), respectively.

Of the 52 amino acids affected by SNPs, 12 were in the TMD, namely c.516C>T（G172G），c.543C>T（Y181Y），c.553T>C（F185L），c.2499G>T（M833I），c.2524T>A（F842I），c.2533C>T（L845F），c.2873C>T（T958M），c.2927A>T（Y976F），c.3201C>T（L1067L），c.3210C>T（F1070F），c.3226T>C（F1076L）, and c.3358C>T（L1120L）；of these, three were in the 3rd TMD, three in the 7th TMD, one in the 9th TMD, and one in the 10th TMD, three in the 11th TMD and one in the 12th TMD (Fig. 1b); the SNPs immersed in the digestive vesicle included 22 synonymous mutations and 15 non-synonymous mutations, and were mainly concentrated in the middle and end of the *Pv*MDR1 peptide chain; the remaining 3 SNPs were on the surface of the outer membrane of the digestive vesicle.


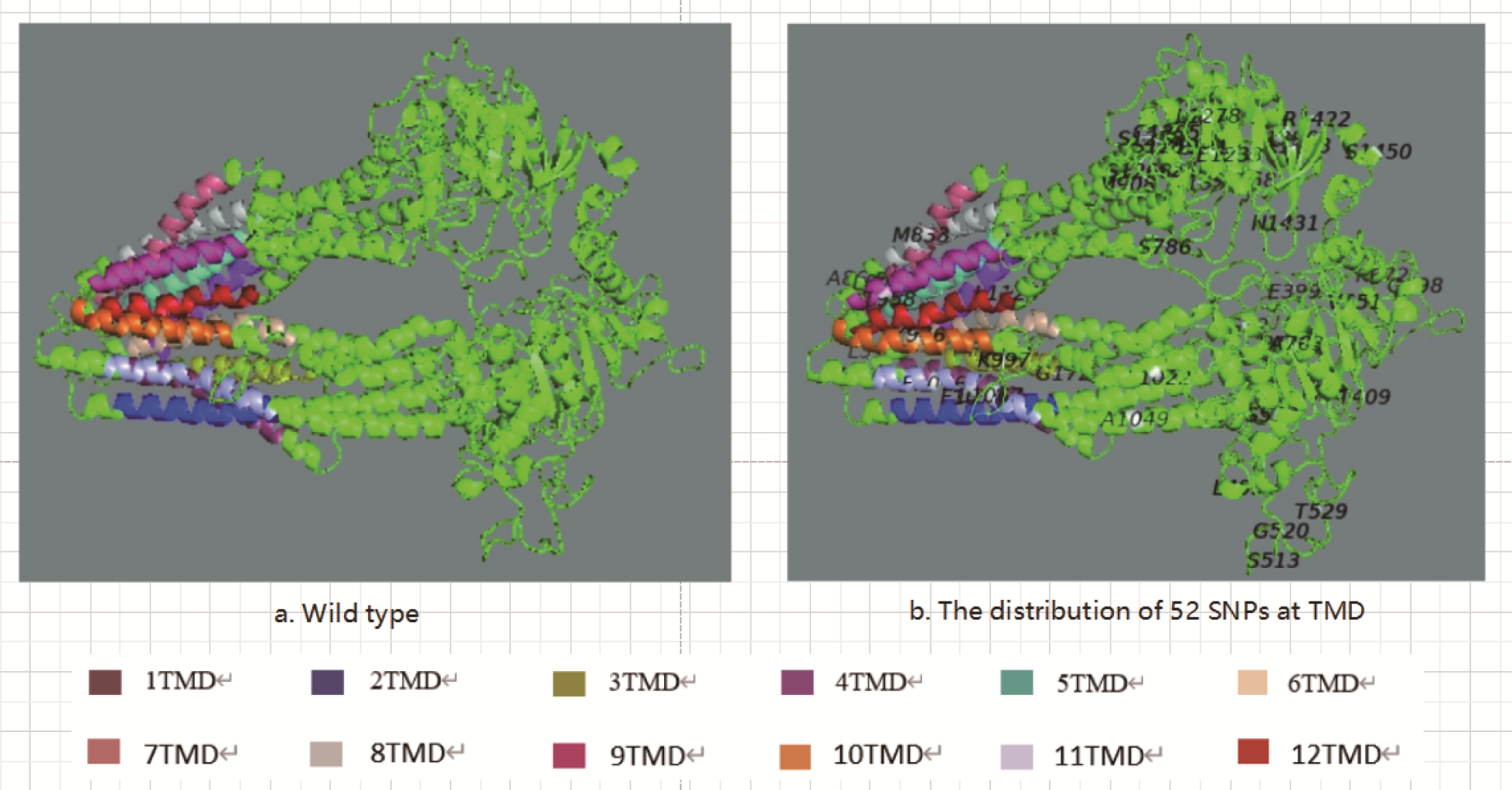


**Fig.1 The spatial distribution of 52 amino acids affected by 52 SNPs at *Pv*MDR1**
